# Tumour-associated high endothelial venules drive portal-specific immune evasion in lymph nodes via ALOX12

**DOI:** 10.1038/s41467-026-72412-w

**Published:** 2026-05-11

**Authors:** Qidong Xia, Jiayao Pan, Xiaoqi Weng, Shunrong Li, Jiaqian Li, Shijian Song, Jiang Li, Boxuan Zhou, Xinwei Liu, Dong-Ming Kuang, Nu Zhang, Jin Jin, Jinting Liu, Zhen Lin, Shubin Yu, Qionglan Tang, Lijuan Bian, Yunjie Zeng, Yu Shi, Yiqing Zheng, Jian-You Liao, Shouping Xu, Shicheng Su

**Affiliations:** 1 Guangdong Provincial Key Laboratory of Malignant Tumor Epigenetics and Gene Regulation, Medical Research Center, Sun Yat-Sen Memorial Hospital, Sun Yat-Sen University, Guangzhou, China; 2 Breast Tumor Center, Sun Yat-Sen Memorial Hospital, Sun Yat-Sen University, Guangzhou, China; 3 Department of Breast Surgery, The First Affiliated Hospital of Zhengzhou University, Zhengzhou, China; 4 School of Life Sciences, Sun Yat-Sen University, Guangzhou, China; 5 Department of Neurosurgery, The First Affiliated Hospital of Sun Yat-Sen University, Guangzhou, China; 6 Department of Neurology, Third Affiliated Hospital, Sun Yat-Sen University, Guangzhou, China; 7 Department of Breast Surgery, The Affiliated Hospital of Guizhou Medical University, Guiyang, China; 8 Cells Vision (Guangzhou) Medical Technology Inc., Guangzhou, China; 9 Department of Pathology, Sun Yat-Sen Memorial Hospital, Sun Yat-Sen University, Guangzhou, China; 10 Institute of Pathology, Southwest Hospital, Third Military Medical University (Army Medical University), Chongqing, China; 11 Department of Otolaryngology, Sun Yat-Sen Memorial Hospital, Sun Yat-Sen University, Guangzhou, China; 12 Department of Breast Surgery, Harbin Medical University Cancer Hospital, Harbin, China; 13 Biotherapy Center, Sun Yat-Sen Memorial Hospital, Sun Yat-Sen University, Guangzhou, China; 14 Department of Immunology and Microbiology, Zhongshan School of Medicine, Sun Yat-Sen University, Guangzhou, China; 15 Department of Infectious Diseases, Third Affiliated Hospital, Sun Yat-Sen University, Guangzhou, China; 16 New Cornerstone Science Laboratory, Sun Yat-Sen University, Guangzhou, China

**Keywords:** Tumour immunology, Cancer microenvironment

## Abstract

High endothelial venules (HEVs) provide another portal for tumour metastasis. However, whether HEVs and other blood vessels exert different effects on tumour escape remains unknown. Here we show that tumour involvement in HEVs is an independent prognostic marker for patients with lymph node (LN)-positive female breast cancer. Tumour cells that spread via HEVs are less immunogenic and more capable of establishing distant metastases than those that spread through non-HEV blood vessels. Mechanistically, the expression of arachidonate 12-lipoxygenase (ALOX12) in HEVs is promoted by tumour-derived semaphorin 3 C (SEMA3C). Reciprocally, ALOX12-derived metabolite 12-hydroxyeicosatetraenoic acid (12-HETE) promotes ADAR1 p150-dsRNA phase separation in tumour cells by selectively binding to ADAR1 p150. Consequently, the immune recognition of dsRNA is reduced because of the increased adenosine-to-inosine (A-to-I) RNA editing in tumour cells. Collectively, our data indicate that a unique lymphatic anatomical structure mediates specific immune evasion of migrating tumour cells.

## Abstract (web summary)

High endothelial venules (HEVs) are an underexplored metastatic route. The authors show that breast cancer cells spreading via HEVs are less immunogenic due to ALOX12-driven immune evasion.

## Introduction

Lymph node (LN) involvement is the strongest prognostic factor for most types of early-stage carcinoma^1–3^. However, the destiny of metastatic cells in LNs remains controversial. A previous thinking is that LN deposits are a dead end for metastatic cancer cells and merely represent a marker for the extent of concomitant dissemination via blood vessels from primary tumours^4,5^. Nevertheless, recent animal studies have shown that tumour cells can exit LNs via LN blood vessels into the systemic circulation^6–10^. However, whether this form of tumour spread also occurs in patients and its clinical significance are largely unclear^6,11^. Moreover, high endothelial venules (HEVs) differ from blood vessels with flat endothelial cells morphologically and functionally^12–18^ and serve as the major route for tumour cells to enter the circulation in LNs^6^. Whether HEVs and other blood vessels exert different effects on disseminating tumour cells is unknown.

Emerging evidence has shown that biomolecular condensates serve as important signal nexuses by concentrating proteins and nucleic acids in a membraneless compartment. These liquid-like droplets of biomolecules are generated by liquid-liquid phase separation (LLPS)^19–23^, in which molecular components demix from the bulk environment, resulting in one phase being highly concentrated in a given set of molecules and one phase being relatively dilute^22,24^. Recent evidence indicates that LLPS is critically involved in regulating immune responses^25^, such as involvement in T cell receptor and B cell receptor pathways^26,27^. Our recent study revealed that condensates of the cytoskeletal machinery in macrophages regulate the phagocytosis of tumour cells^28^. Therefore, the essential role of LLPS in pathophysiological conditions emphasises the need to further explore how LLPS mediates tumour immunity.

A-to-I RNA editing is an evolutionarily conserved post-transcriptional modification mediated by adenosine deaminase acting on RNA (ADAR) enzymes that convert adenosine to inosine in dsRNA^29,30^. Most editing occurs in inverted repeats that often form dsRNA, with ADAR1 serving as the predominant enzyme for these sites^31^. Endogenous dsRNA derived predominantly from endogenous retroelements (EREs) triggers the innate immune response via RNA sensors^32–36^. A-to-I editing by ADAR1 masks these self-dsRNA structures, preventing recognition by RNA sensors^32,35,37–39^. Perturbation of ADAR1 reportedly reduces A-to-I editing level, leading to accumulation of unedited dsRNA species, activation of interferon (IFN) pathway and sensitising tumour cells to T cell killing in multiple tumour cell lines, such as murine melanoma (B16F10), murine colon carcinoma (MC38, CT26), human tongue cancer (CAL27, HSC4), human breast cancer (HDQ-P1, MDA-MB-468), human pancreatic cancer (SW1990, PATU-8902) and human lung cancer (HCC366, NCI-H1650, NCI-H196, NCI-H596, SW900, HCC1438)^40–42^. In addition, deletion of both *Ifnar2* and *Ifngr1* abrogated the response of *Adar1*-knockout tumours to immunotherapy, indicating that the IFN pathway is indispensable for promoting the immunogenicity of tumour cells in *Adar1*-knockout tumours^40^. Although this frontier is still in its infancy, elucidating how these immunostimulatory nucleic acids are regulated will provide a better understanding of the immune response.

Beyond their classical roles in bioenergetics and biosynthesis, metabolites can modify proteins and thereby influence their functions in immune-related diseases, often accompanied by increased expression of enzymes involved in glucose, lipid, and amino acid metabolism^43^. Some intracellular metabolites also function as oncometabolites, promoting tumour progression through epigenetic and post-translational mechanisms^44^. Nonetheless, the contribution of endogenous signalling molecules to these processes via LLPS has been rarely reported.

LNs are one of the most important sites involved in the tumour immune response. Here, we investigated the clinical significance of tumour dissemination via HEVs in LNs and its underlying mechanisms. By deep learning algorithm analysis of sentinel LNs, multi-omics profiling and injecting photoconvertible cells into *Alox12*^*fl/fl*^; *Chst4-CreERT2* mice, we revealed that HEVs, rather than non-HEV blood vessels, shield the immune vulnerability as “Achilles’ heel” of circulating tumour escapees. Notably, the ALOX12-derived 12-HETE was responsible for ALOX12-mediated immune evasion of tumour cells, and 12-HETE promoted A-to-I RNA editing in tumour cells by selectively driving ADAR1 p150-dsRNA LLPS. Our findings indicate that the ALOX12-12-HETE signal, predominantly produced by HEVs, serves as an attractive therapeutic target for metastasis and provide insights into the mechanisms of 12-HETE as an endogenous signalling molecule in mediating immune evasion by regulating ADAR1 p150-dsRNA LLPS related to epigenetic A-to-I RNA editing.

## Results

### HEV involvement stratifies the prognosis of patients with LN-positive breast cancer

**Fig. 1:**
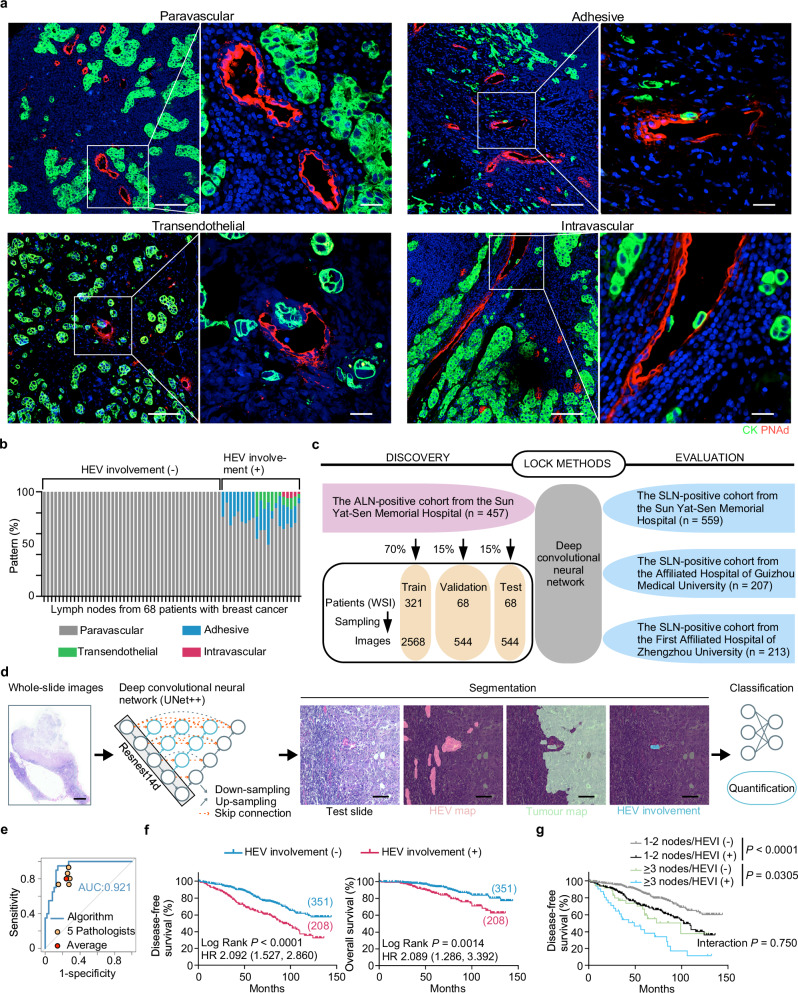
HEV involvement stratifies the prognosis of patients with LN-positive breast cancer. Representative immunofluorescence images (**a**) and quantitation (**b**) of four patterns of relationship between tumour cells and HEVs in SLNs from 68 patients with breast cancer. Scale bars, 50 μm (main panels) and 20 μm (magnified regions). **c** An overall workflow for developing a deep learning system for detecting HEV involvement. **d** Overview of the deep convolutional neural network for HEV involvement detection. The H&E whole-slide images were segmented using UNet++ networks pretrained on a breast cancer histology image online dataset and fine-tuned on our own dataset of slides of metastatic LNs from 457 patients with breast cancer. The output layer produced a tumour probability map and an HEV probability map, and was post-processed to generate HEV involvement locations and scores. Then, HEV involvement in WSIs was classified by a convolutional neural network, and tumour lesion areas were calculated to evaluate the nodal tumour burden. Scale bars, 2000 μm (left) and 200 μm (right). **e** The blue receiver operating characteristic (ROC) curve represents the deep learning algorithm’s diagnosis. The orange dots represent 5 pathologists’ diagnoses with a single sensitivity-specificity score. The red dot is the average score for all pathologists. **f** Kaplan–Meier analysis for disease-free survival and overall survival in LN-positive breast cancer patients with or without HEV involvement from the Sun Yat-Sen Memorial Hospital. **g** Kaplan–Meier analysis for disease-free survival in patients with different numbers of positive LNs with or without HEV involvement (HEVI) from the Sun Yat-Sen Memorial Hospital. The interaction *P*-values were evaluated using Cox regression with the nodes (positive LN number)-by-biomarker (HEVI) interaction term. *P*-values were calculated by the log-rank Mantel–Cox test (**f**, **g**). HR hazard ratio.

Immunofluorescence analysis of metastatic sentinel lymph nodes (SLNs) from breast cancer patients for cytokeratin (CK) and peripheral lymph node addressin (PNAd) revealed four different location relationships between tumour cells and HEVs, including paravascular, adhesive, transendothelial and intravascular patterns (Fig. 1a). We categorised the adhesive, transendothelial and intravascular patterns as HEV involvement (Fig. 1b). To investigate the clinical significance of HEV involvement, we used an automated image analysis method^45–47^ to evaluate haematoxylin and eosin (H&E) whole-slide images (WSIs) of metastatic axillary LNs (ALNs) from 457 patients who received ALN dissection without SLN biopsies (the ALN-positive cohort) (Supplementary Dataset 1). We used these samples to develop the system because we can maximise the independent SLN sample size during the evaluation stage. First, we randomly split metastatic LNs into training (70%), validation (15%) and test (15%) sets at the patient level (Fig. 1c). Our framework comprises three phases: UNet++ networks acted to segment tumour and HEV regions; a convolutional neural network was employed to classify HEV involvement; and a computational module was used to quantitate tumour areas (Fig. 1d). After full training, the classification network achieved an area under the curve (AUC) score of 92.1% on the test set, surpassing the performance of most compared pathologists (Fig. 1e).

Using this deep learning model and metastatic SLNs from 559 breast cancer patients (the SLN-positive cohort) (Supplementary Table 1), we found that HEV involvement in SLNs was associated with shorter disease-free survival (DFS) and overall survival (OS) in SLN-positive breast cancer patients (Fig. 1f). Multivariate Cox regression confirmed HEV involvement as an independent predictor for both DFS and OS (Supplementary Tables 2 and 3). Moreover, the metastatic tumour area did not differ significantly between patients with and without HEV involvement in SLNs (Supplementary Table 1). A stratified analysis revealed that HEV involvement predicted a poor outcome in patients even with the same number of positive SLNs (Fig. 1g). In addition, the *P*-value for interaction analysis between the number of positive SLNs and HEV involvement was not significant, indicating that HEV involvement was associated with a worse prognosis independent of nodal tumour burden (Fig. 1g). The prognostic value of HEV involvement was validated in two other independent external cohorts comprising 207 and 213 patients (Fig. 1c and Supplementary Fig. 1a, b). Collectively, these findings indicate that HEV involvement is an independent prognostic factor for stratifying the prognosis of patients with LN-positive breast cancer.

### HEV-emigrant tumour cells evade immune surveillance

**Fig. 2:**
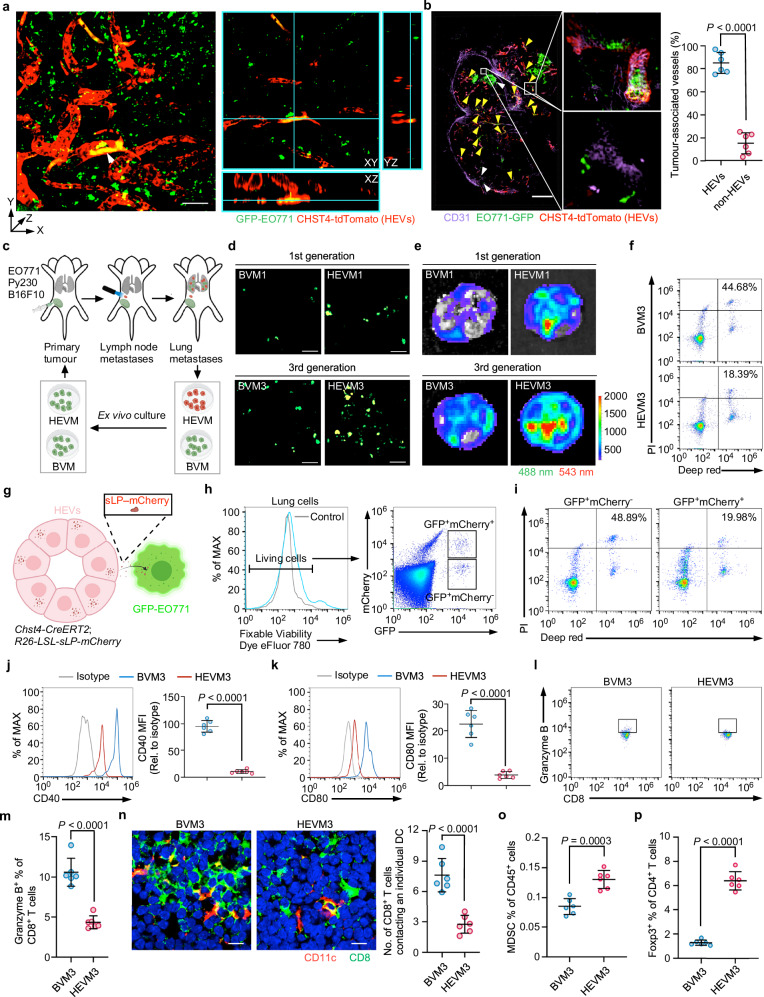
HEV-emigrant tumour cells evade immune surveillance. **a** Intravital multiphoton microscopy of GFP-EO771 cells and HEVs in the LNs of *Chst4-tdTomato* mice. Arrowheads indicate HEV-intravasating tumour cells. Orthogonal views (XY, XZ, YZ) of single EO771 cells intravasating blood vessels are shown. Scale bar, 50 μm. **b** LNs from GFP-EO771-bearing *Chst4-tdTomato* mice were stained for CD31. Confocal images were tiled to reconstruct a 3D LN vascular network. Yellow arrowheads indicate tumour-associated HEVs and white arrowheads indicate tumour-associated non-HEV blood vessels. Scale bar, 500 μm. Each dot represents data pooled from three sections from each mouse. **c** The schematic of iterative in vivo selection of Blood Vessel Metastatic (BVM) and HEV Metastatic (HEVM) tumour cells using photoconversion of TDLNs. Representative immunofluorescence images (**d**) and bioluminescent images (**e**) of lungs harvested from mice inoculated with the indicated EO771 derivatives via fat pad injection. Scale bars, 50 μm. Quantitation in Supplementary Fig. 2e and 3a. **f** Representative flow cytometry plots for CD8^+^ T cell-mediated cytotoxicity to indicated EO771 derivatives. The unstimulated T cell control and quantitation in Supplementary Fig. 3e, f. **g** The design of the sLP-mCherry labelling system. **h** Flow cytometry gating strategy for the isolation of lung metastatic tumour cells disseminating via HEVs (GFP^+^mCherry^+^) and non-HEV vessels (GFP^+^mCherry^−^). **i** Representative flow cytometry plots for CD8^+^ T cell-mediated cytotoxicity to indicated tumour cells. The unstimulated T cell control and quantitation in Supplementary Fig. 3o, p. Immune profiling in mediastinal LNs of BVM3 and HEVM3-bearing mice, including CD40 and CD80 expression in CD103^+^ DCs (**j**, **k**), Granzyme B^+^ CD8^+^ T cells (**l**, **m**), CD8^+^ T cell-DC interaction (**n**), MDSCs (**o**) and Treg cells (**p**). Scale bar, 10 μm. Gate strategy, isotype controls and fluorescence minus one (FMO) controls are provided in Supplementary Fig. 4. *n* = 6 mice per group (**a**, **b**, **j**, **k**, **m**–**p**). Mean ± SD (**b**, **j**, **k**, **m**–**p**). *P*-values were calculated by a two-tailed unpaired *t*-test (**b**, **j**, **k**, **m**–**p**).

To recapitulate human HEV involvement in vivo, we constructed a line of mice with specific expression of tdTomato in HEVs. The tdTomato construct was under the control of *Chst4*, which is an HEV-restricted sulfotransferase (Supplementary Fig. 2a). Using time-lapse multiphoton intravital microscopy, we observed that green fluorescent protein (GFP)-expressing EO771 cells moved towards and intravasated HEVs at an average speed of approximately 9 μm/h in the *Chst4-tdTomato* mice (Fig. 2a and Supplementary Fig. 2b and Supplementary Movie 1). The presence of tumour cells within the HEV lumen was further confirmed by immunofluorescence analysis (Supplementary Fig. 2c).

HEVs were reported to be the major route for tumour cells to enter the circulation^6^. We found that 84.99 ± 9.181% of the blood vessels involved by tumour cells were CD31^+^tdTomato^*+*^ HEVs, whereas only 15.01 ± 9.181% of the blood vessels involved by tumour cells were CD31^+^tdTomato^−^ non-HEV blood vessels (Fig. 2b). To visualise tumour cells that preferentially spread via LN blood vessels, we stably expressed the photoactivatable fluorescent protein Dendra2H2B, which is converted from green to red fluorescence after being exposed to 405 nm light^7^, in EO771 cells, and implanted these cells in mammary fat pads. After tumour cells colonised the LN, we exposed the draining inguinal LNs to 405 nm light every day for 7 consecutive days (Fig. 2c). We observed that tumour cells in LNs but not those in primary lesions underwent photoconversion (Supplementary Fig. 2d). Dendra2H2B-expressing cells gradually lost red fluorescence after being cultured ex vivo^7^. To enrich tumour cells that preferentially spread via LN blood vessels, we repeated the in vivo selection by harvesting green^+^red^+^ HEV metastatic (HEVM) tumour cells and green^+^red^−^ blood vessel metastatic (BVM) tumour cells from lung metastases. When all red-emitting tumour cells returned to green after 7 days of ex vivo culture, HEVM1 and BVM1 cells (generation 1) were subjected to 2 more rounds of in vivo selection yielding HEVM3 and BVM3 cell derivatives (generation 3) (Fig. 2c). After 7 consecutive days photoconversion of LN, confocal microscopy revealed more photoconverted tumour cells in the lung metastases of the mice inoculated with HEVM1 or HEVM3 cells compared with those inoculated with BVM1 or BVM3 cells (Fig. 2d and Supplementary Fig. 2e), and these results were corroborated by flow cytometric analysis (Supplementary Fig. 2f). Furthermore, HEVM1 or HEVM3 cells exhibited greater metastasis than BVM1 or BVM3 cells at humane end point, while the difference between BVM3 and HEVM3 cells was more pronounced than the difference between BVM1 and HEVM1 cells was (Fig. 2e and Supplementary Fig. 3a). No appreciable difference in cell motility or mutation status of critical genes was observed between HEVM3 and BVM3 cells (Supplementary Fig. 3b, c and Supplementary Table 4). Importantly, the proliferation rates of HEVM3 and BVM3 cells were comparable (Supplementary Fig. 3d), indicating that the different proportions of green^+^red^+^ and green^+^red^−^ tumour cells in lung metastases are not caused by different decay rates of red fluorescence, which is dependent on the rate of proliferation^48^. In comparison, HEVM3 cells were more resistant to the T cell-mediated cytotoxicity in vitro (Fig. 2f and Supplementary Fig. 3e, f). In vivo, we detected fewer infiltrating CD8^+^ T and natural killer (NK) cells in the lung metastases formed by HEVM1 or HEVM3 cells compared with the lung metastases formed by BVM1 or BVM3 cells (Supplementary Fig. 3g, h). Moreover, the differences in metastasis between HEVM3 and BVM3 cells were not detected in immunodeficient NOD/SCID mice (Supplementary Fig. 3i), indicating that the difference in metastatic capacity between these derivatives is attributed to their distinctive immunogenicity. The reduced immunogenicity of tumour cells that enter circulation via LN blood vessels compared with that of tumour cells disseminated via primary tumour blood vessels was also observed in additional models, including Py230 breast cancer cells and B16F10 melanoma cells (Supplementary Fig. 3j–m), indicating this phenomenon is not model-specific. To more specifically trace tumour cells disseminating through HEVs, we developed another labelling system by constructing a line of *R26-LSL-sLP-mCherry* mice with expression of a secreted fluorescent mCherry protein fused to a modified lipid-permeable transactivator of transcription (TATk) peptide (sLP-mCherry) under a stop codon^49^ (Fig. 2g). *Chst4-CreERT2* mice were crossed to *R26-LSL-sLP-mCherry* mice to obtain conditional *Chst4-CreERT2*;*R26-LSL-sLP-mCherry* mice, in which tamoxifen treatment induced Cre-mediated excision of the stop cassette and enabled specific expression of sLP-mCherry in HEVs. We then implanted luciferase-GFP-labelled EO771 breast cancer cells into the mammary fat pad of these mice. The sLP-mCherry protein from HEVs was taken up by passing tumour cells (Supplementary Fig. 3n). This sLP-mCherry labelling system enabled specific discrimination of lung metastatic tumour cells disseminating via HEVs (GFP^+^mCherry^+^) compared with tumour cells disseminating via other blood vessels or lymphatics (GFP^+^mCherry^−^) using fluorescence-activated cell sorting (FACS) (Fig. 2h). Functionally, GFP^+^mCherry^+^ tumour cells were also more resistant to the T cell-mediated cytotoxicity in vitro compared with GFP^+^mCherry^−^ tumour cells (Fig. 2i and Supplementary Fig. 3o, p). Moreover, when re-implanted into the mammary fat pad, GFP^+^mCherry^+^ cells exhibited greater metastatic potential than GFP^+^mCherry^−^ cells at humane end point (Supplementary Fig. 3q). Collectively, evidence from both the sLP-mCherry labelling system and the photoconversion model consistently confirmed the reduced immunogenicity and enhanced metastatic capacity of HEV-emigrant tumour cells.

To investigate whether HEVM3 cells stimulate a weaker immune response in LNs draining the lungs, we first evaluated the activation of migratory CD103^+^ dendritic cells (DCs) (CD24^+^, Ly6C^−^, CD11c^+^, MHCII^hi^ and CD103^+^), which acquire tumour material in the lungs and subsequently migrate to mediastinal LNs to initiate the immune response^50,51^. Our results revealed that CD103^+^ DCs from the mediastinal LNs of HEVM3-bearing mice expressed lower levels of costimulatory molecules, including CD40 and CD80 (Fig. 2j, k and Supplementary Fig. 4a). In parallel, the frequency of Granzyme B^+^ CD8^+^ T cells was reduced (Fig. 2l, m and Supplementary Fig. 4b, c), and smaller clusters of DCs with CD8^+^ T cells were detected in mediastinal LNs in HEVM3-bearing mice (Fig. 2n). To further delineate the immune landscape, we additionally analysed immunosuppressive cell populations. Flow cytometry showed that HEVM3-bearing mice harboured more CD11b^+^MHCII^−^Ly6C^hi/lo^ myeloid-derived suppressor cells (MDSCs) (Fig. 2o and Supplementary Fig. 4a) and more Foxp3⁺CD4⁺ regulatory T (Treg) cells in mediastinal LNs compared with BVM3-bearing mice (Fig. 2p and Supplementary Fig. 4d, e). Collectively, these results indicate that HEV-emigrant tumour cells are more likely to evade immune surveillance compared with tumour cells disseminating via non-HEV blood vessels.

### Tumour-associated HEV-derived ALOX12 suppresses the immunogenicity of tumour cells via 12-HETE

**Fig. 3:**
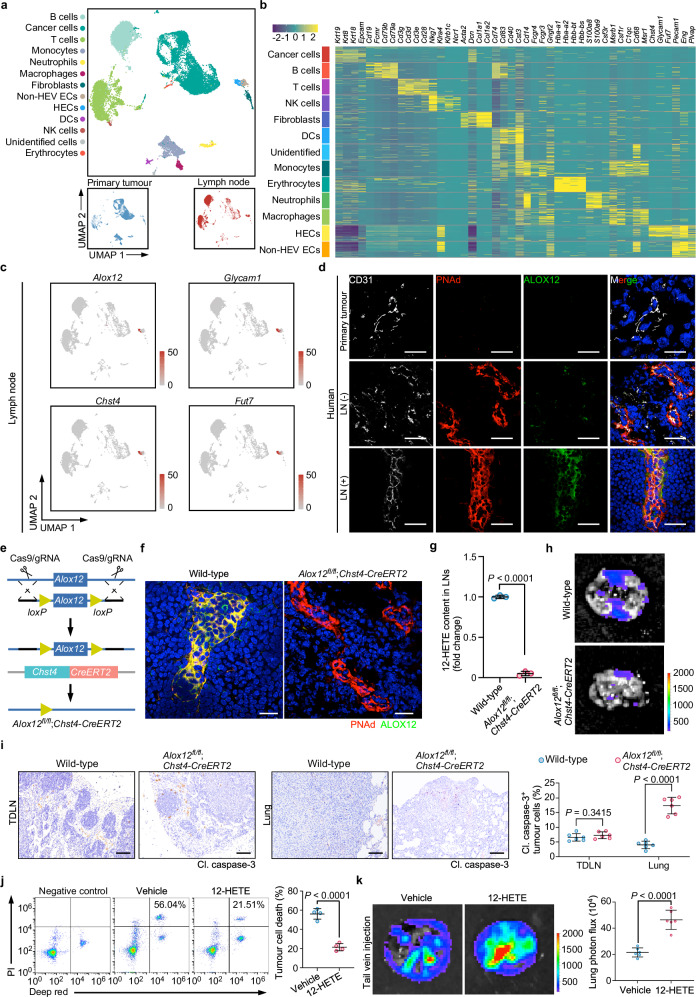
Tumour-associated HEV-derived ALOX12 suppresses the immunogenicity of tumour cells via 12-HETE. **a** UMAP plots showing cell types identified from paired EO771 tumours and metastatic LNs from 6 mice. ECs, endothelial cells. **b** Heatmap showing relative expression of marker genes across cell clusters in scRNA-seq of primary tumours and LNs. **c** UMAP plots showing the expression levels of indicated genes for HECs in LNs. HECs are identified as an endothelial cell subpopulation expressing *Glycam1*, *Chst4* and *Fut7*. **d** Representative immunofluorescence images show the colocalisation of CD31, PNAd and ALOX12 in human primary tumours, LN without (LN^−^) or with metastases (LN^+^). Scale bars, 50 μm. Isotype control staining and quantitation in Supplementary Fig. 5f, h. **e** Construction diagram of *Alox12*^*fl/fl*^;*Chst4-CreERT2* mice. **f** Representative immunofluorescence images showing ALOX12 levels in HECs in LNs from wild-type and *Alox12*^*fl/fl*^;*Chst4-CreERT2* mice. Scale bars, 20 μm. **g** Relative content of 12-HETE in LNs from wild-type and *Alox12*^*fl/fl*^;*Chst4-CreERT2* mice. **h** Representative bioluminescent images of lungs harvested from indicated mice inoculated with luciferase-expressing EO771 cells in mammary fat pads. Quantitation in Supplementary Fig. 7e. **i** Representative images (left) and quantitation (right) of immunohistochemistry staining for cleaved caspase-3 (Cl. caspase-3) in the TDLNs and lung metastases of indicated mice inoculated with EO771 cells. Scale bar, 100 μm. Each dot represents data pooled from 5 distinct areas from a slice and 3 slices per mouse. **j** Representative flow cytometry plots (left) and quantitation (right) for CD8^+^ T cell-mediated cytotoxicity to vehicle-treated and 12-HETE-treated EO771 cells. Unstimulated T cells were used as a negative control. **k** Representative bioluminescent images (left) and quantitation (right) of lungs harvested from *Alox12*^*fl/fl*^;*Chst4-CreERT2* mice inoculated with vehicle-treated and 12-HETE-treated EO771 cells via intravenous injection. *n* = 3 independent experiments (**g**); *n* = 6 mice per group (**i**, **k**); *n* = 4 independent experiments (**j**). Mean ± SD (**g**, **i**–**k**). *P*-values were calculated by a two-tailed unpaired *t*-test (**g**, **i**–**k**).

Endothelial cells have been reported to facilitate cancer progression by releasing diverse factors in addition to their metabolic support functions^52,53^. Integrating our data from clinical specimens and mouse models, we propose that HEVs may transmit molecular cues to tumour cells that reduce their immunogenicity. To explore potential HEV-derived factors mediating this effect, we compared the transcriptomes of blood vessel endothelial cells (BECs) isolated from mammary blood vessels and HEV endothelial cells (HECs) isolated from the LNs of the mice with or without tumours (Supplementary Fig. 5a). We identified 115 genes that were elevated in HECs compared with BECs and increased in tumour-bearing LNs compared with normal LNs (Supplementary Fig. 5b and Supplementary Dataset 2). We then focused on *Alox12*, because it was the top-ranked elevated gene in tumour-associated HECs (Supplementary Fig. 5b). Furthermore, ALOX12 is a lipoxygenase that converts arachidonic acid into 12-HETE, which is released to exert biological effects and potentially perceived by tumour cells^54–57^. Single-cell RNA sequencing (scRNA-seq) of primary tumours and paired metastatic LNs also revealed that *Alox12* was predominantly expressed in HECs in metastatic LNs, but was not expressed in BECs of primary tumours (Fig. 3a–c and Supplementary Fig. 5c–e). This finding was confirmed in both clinical samples and mouse tissues via immunofluorescence staining (Fig. 3d and Supplementary Fig. 5f–i) and quantitative real-time PCR with reverse transcription (qRT-PCR) (Supplementary Fig. 5j, k). To increase the resolution of endothelial cells in the cell atlas analysis, we performed scRNA-seq of sorted BECs from metastatic LNs and found that *Alox12* was predominantly expressed in HECs rather than other EC subsets in metastatic LNs (Supplementary Fig. 6a, b). We injected EO771 cells expressing the firefly luciferase *Luc2* gene into mouse fat pads. No *Luc2* signals were detected in draining LNs 11 days after inoculation, while *Luc2* signals were detected in LNs 2 weeks after inoculation as previously reported^58^. Thus, we defined these LNs as pre-metastatic LNs and metastatic LNs, respectively (Supplementary Fig. 6c). Consistently, high-resolution immunofluorescence images of the whole mouse LN section showed that basic expression of ALOX12 was mainly detected in PNAd^+^ HECs and was also moderately detected in CX40^+^EMCN^−^PNAd^−^ arterial ECs in homeostatic LNs. More importantly, ALOX12 level didn’t increase in pre-metastatic LNs but was markedly elevated in HECs rather than arterial ECs in metastatic LNs (Supplementary Fig. 6d, e). These results were corroborated by flow cytometry (Supplementary Fig. 6f–h) and qRT-PCR analysis (Supplementary Fig. 6i). Together, our data demonstrates that ALOX12 is predominantly expressed in HEVs of metastatic LNs.

Tumour cell-derived ALOX12 has been linked to the malignant behaviour in several cancers^59–61^, but its function in HEVs has not been investigated. We revealed that blocking 12-HETE production with selective ALOX12 inhibitors ML355 and CDC^62–64^ inhibited the lung metastasis and increased CD8^+^ T and NK cells infiltration in the lung metastases, and these effects could be rescued by 12-HETE replenishment (Supplementary Fig. 7a, b), indicating HEV-derived ALOX12/12-HETE may suppress the immunogenicity of tumour cells. To directly assess the role of HEV ALOX12 in tumour metastasis, we generated mice carrying *loxP*-flanked *Alox12* alleles crossed with *Chst4-CreERT2* mice to drive selective deletion of *Alox12* in HECs after tamoxifen treatment (Fig. 3e and Supplementary Fig. 7c). ALOX12 expression in HEVs was abolished in the *Alox12*^*fl/fl*^;*Chst4-CreERT2* mice after tamoxifen treatment, whereas ALOX12 expression in platelets was intact (Fig. 3f and Supplementary Fig. 7d). HEV-conditional *Alox12* knockout, which resulted in a marked reduction in its metabolite, 12-HETE, in LNs (Fig. 3g), significantly reduced tumour metastases (Fig. 3h and Supplementary Fig. 7e). Furthermore, *Alox12* knockout markedly reduced the number of green^+^red^+^ metastatic cells but not green^+^red^−^ ones in the lungs, indicating that *Alox12* knockout mainly affects metastasis through HEVs (Supplementary Fig. 7f). By comparison, primary tumour growth (Supplementary Fig. 7g), HEV density in LNs (Supplementary Fig. 7h), metastatic burden in LNs (Supplementary Fig. 7h) and tumour cells which intravasated into HEVs (Supplementary Fig. 7i) remained unchanged. Moreover, more CD8^+^ T cells and NK cells were observed in lung metastatic lesions in the *Alox12*^*fl/fl*^;*Chst4-CreERT2* mice compared with wild-type littermates (Supplementary Fig. 7j). More importantly, we detected higher proportion of tumour cells expressing apoptosis marker (cleaved caspase-3) in lung metastatic lesions in *Alox12*^*fl/fl*^;*Chst4-CreERT2* mice compared with wild-type littermates (Fig. 3i). Although we observed that a small number of tumour cells in tumour-draining LNs (TDLNs) expressed cleaved caspase-3, there was no significant difference of cleaved caspase-3 expression between the LN metastases in *Alox12*^*fl/fl*^;*Chst4-CreERT2* and wild-type mice (Fig. 3i). These data suggest that although tumour cells can be eliminated both in the TDLNs and lungs, the inhibiting effect of HEV-derived ALOX12 on immunogenicity of tumour cells occur predominantly in the lungs after tumour cells enter the circulation via HEVs.

To further evaluate the specific role of 12-HETE produced by HEV-derived ALOX12 in immunogenicity suppression, we treated tumour cells with 12-HETE in vitro. The results revealed that tumour cells treated with 12-HETE were more resistant to the cytotoxicity of CD8^+^ T cells compared with vehicle-treated cells (Fig. 3j). To determine the impact of 12-HETE on metastatic immune evasion, EO771 cells were exposed to 12-HETE or vehicle for 48 h and subsequently injected in equal numbers into *Alox12*^*fl/fl*^;*Chst4-CreERT2* mice via the tail vein. A markedly greater metastatic load was observed in the 12-HETE group compared with controls (Fig. 3k). To investigate how rapidly immune suppression is established after tumour cells intravasate through HEVs, we mimicked the exposure of tumour cells to HEV-derived 12-HETE by pretreating them with 12-HETE in vitro and injecting them into mice via the tail vein. We then performed a time-course analysis of Granzyme B⁺ CD8⁺ T cell activation in mediastinal LNs at days 1, 2, and 3 after injection. On days 1 and 2, Granzyme B⁺ CD8⁺ T cells were not significantly activated in either group (Supplementary Fig. 7k). By day 3, both vehicle-pretreated and 12-HETE-pretreated tumour cell groups showed increased Granzyme B⁺ CD8⁺ T cell levels, reflecting the onset of adaptive immune responses. However, the 12-HETE-pretreated group exhibited a markedly reduced level of Granzyme B⁺ CD8⁺ T cells compared with the vehicle-pretreated group, indicating that HEV-secreted 12-HETE diminishes tumour cell immunogenicity and attenuates early T cell activation (Supplementary Fig. 7k). Collectively, we demonstrate that ALOX12 is predominantly expressed in tumour-associated HEVs of metastatic LNs and induces immune evasion of migrating tumour cells via 12-HETE.

### Tumour-derived SEMA3C upregulates ALOX12 in tumour-associated HEVs

**Fig. 4:**
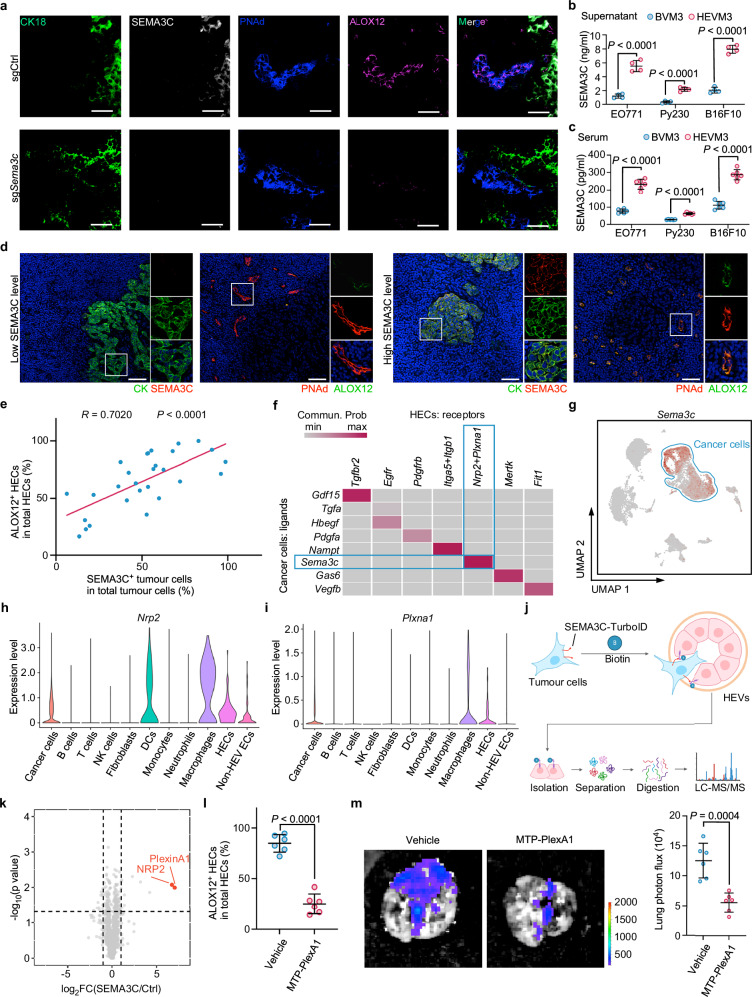
Tumour-derived SEMA3C upregulates ALOX12 in tumour-associated HEVs. **a** Representative immunofluorescence images of LNs stained with the indicated antibodies from mice injected with EO771 cells with or without *Sema3c* knockout. Scale bars, 20 μm. Quantitation in Supplementary Fig. 8d, e. SEMA3C levels in supernatant of indicated tumour cells (**b**) and in the serum of corresponding tumour-bearing mice (**c**). **d** Representative immunofluorescence staining for CK and SEMA3C (left) and ALOX12 and PNAd (right) in human metastatic LNs. Scale bars, 50 μm. Zoomed-in images demonstrate SEMA3C expression in CK^+^ tumour cells (left) and ALOX12 expression in PNAd^+^ HEVs (right). **e** The correlation between the proportion of ALOX12^+^ HECs and the proportion of SEMA3C^+^ tumour cells from human metastatic LNs (*n* = 28 patients). Each dot represents data pooled from 5 distinct areas from a slice and 3 slices per patient. **f** Cell-cell communication analysis on the basis of our scRNA-seq data using the CellChat algorithm. The scale indicated communication probability (Commun. Prob.) between tumour cells and HECs by assessing the averaged expression of ligands and receptors. **g** UMAP plots showing the expression level of *Sema3c* in cancer cells. Expression of *Nrp2* (**h**) and *Plxna1* (**i**) in different cell types was identified by our scRNA-seq. **j** Schematic representation of the TurboID-based proximity labelling strategy. **k** Volcano plot showing enriched biotinylated proteins in HECs from metastatic LNs of mice bearing SEMA3C-TurboID-overexpressing EO771 cells. HECs from metastatic LNs of mice bearing secreted TurboID-overexpressing EO771 cells were used as a control. **l** The quantitation of the proportion of ALOX12^+^ HECs in LNs of EO771-bearing mice treated with or without MTP-PlexA1. Each dot represents data pooled from 5 distinct areas from a slice and 3 slices per mouse. **m** Representative bioluminescent images (left) and quantitation (right) of lungs harvested from EO771-bearing mice treated with or without MTP-PlexA1. *n* = 4 independent experiments (**b**); *n* = 6 mice per group (**c**, **l**, **m**); *n* = 3 independent experiments (**k**). Mean ± SD (**b**, **c**, **l**, **m**). *P*-values were calculated by a two-tailed unpaired *t*-test (**b**, **c**, **k**–**m**) and two-sided Spearman correlation (**e**).

To identify the factor(s) that upregulate ALOX12, we analysed online mRNA profiling data of 90 metastatic LNs from breast cancer patients^65^. Among the top 5 secreted factors with the strongest correlation with *ALOX12* in metastatic LNs^66^ (Supplementary Fig. 8a), deleting *Sema3c* in tumour cells rather than other factors reduced the expression of ALOX12 in HEVs of the HEVM3 tumour-bearing mice (Fig. 4a and Supplementary Fig. 8b–d). Of note, we observed no appreciable effect of SEMA3C knockout on LN metastases per se (Fig. 4a and Supplementary Fig. 8e). Enzyme-linked immunosorbent assay (ELISA) confirmed the overexpression of SEMA3C in derivatives that preferentially metastasise through HEVs from 3 different cell lines (Fig. 4b) and in the serum of corresponding tumour-bearing mice (Fig. 4c). Clinically, the proportion of ALOX12^+^ HECs was significantly correlated with the proportion of SEMA3C^+^ tumour cells in human metastatic LNs (Fig. 4d, e). Consistent with a tumour-induced mechanism, immunofluorescence analysis of mouse lymph nodes harvested at an early stage of metastasis revealed that high ALOX12 expression in HEVs was spatially restricted to vessels in close proximity to sparse tumour cells (Supplementary Fig. 8f).

Neuropilin 2 (NRP2) is the receptor of SEMA3C, and PlexinA1 is the functional co-receptor of SEMA3C^67^. Interestingly, we analysed cancer cell-to-HEC signals using our scRNA-seq data via CellChat^68^, which allowed us to predict cellular interactions by linking ligand and receptor expression and found that *Sema3c*-(*Nrp2*+*Plxna1*) was one of the most significant ligand-receptor pairs (Fig. 4f). Correspondingly, scRNA-seq analysis revealed that *Sema3c* was almost exclusively expressed in cancer cells (Fig. 4g), whereas only HECs and macrophages expressed high levels of both *Nrp2* and *Plxna1* (Fig. 4h, i). In addition, the predominant expression of *ALOX12* and *PLXNA1* in HECs and *SEMA3C* in the tumour cells was further corroborated by a publicly available scRNA-seq dataset of human metastatic LNs^69^ (Supplementary Fig. 8g).

To further substantiate the role of tumour-derived SEMA3C in regulating HEV ALOX12 expression within metastatic lymph nodes, we employed a TurboID-based proximity labelling approach coupled with liquid chromatography-tandem mass spectrometry (LC-MS/MS). EO771 cells expressing an SEMA3C-TurboID fusion protein were implanted into the mammary fat pad, and upon LN metastasis formation, intraperitoneal injection of exogenous biotin was performed to biotinylate proteins in the close vicinity of SEMA3C-TurboID. Primary HECs were then isolated from metastatic LNs, and TurboID-labelled proteins interacting with tumour-derived SEMA3C were identified by LC-MS/MS (Fig. 4j). Notably, NRP2 and PlexinA1 were the most prominently enriched biotinylated proteins in HECs from metastatic LNs of mice bearing SEMA3C-TurboID-overexpressing EO771 cells, indicating that tumour-derived SEMA3C is spatially proximal to and engages NRP2/PlexinA1 signalling on HEVs in vivo (Fig. 4k and Supplementary Dataset 3). Furthermore, treatment with the peptidic antagonist MTP-PlexA1 against PlexinA1 substantially suppressed ALOX12 expression in tumour-associated HECs (Fig. 4l) and lung metastases in the HEVM3 tumour-bearing mice (Fig. 4m). Collectively, these results support that tumour-derived SEMA3C engages NRP2/PlexinA1 signalling to upregulate ALOX12 expression in tumour-associated HEVs.

### 12-HETE suppresses the interferon response by promoting A-to-I RNA editing

**Fig. 5:**
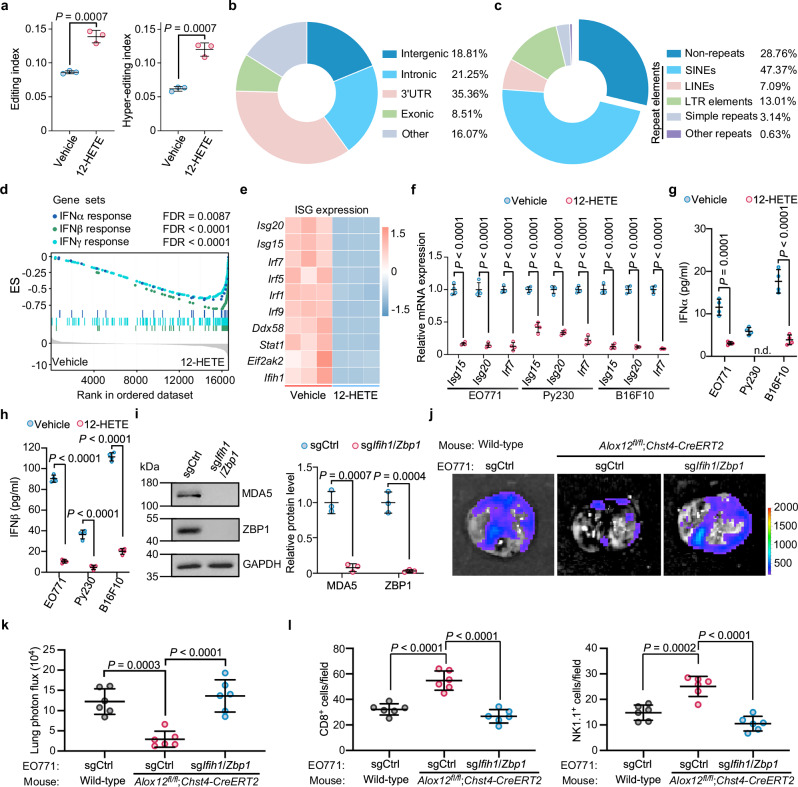
12-HETE suppresses the interferon response by promoting A-to-I RNA editing. **a**–**c** The total A-to-I editing sites were determined using total stranded RNA-sequencing data from EO771 cells treated with vehicle or 12-HETE. Quantitation of the global A-to-I editing level (left) and hyper-editing level (right) (**a**). Pie chart showing the genomic distribution of high-confidence A-to-I RNA editing sites (**b**). Pie chart showing the distributions of the high-confidence A-to-I RNA editing sites in non-repeats and indicated repetitive regions (**c**). **d** GSEA analysis of the type I and II IFN response in EO771 cells treated with vehicle or 12-HETE. ES, enrichment score. FDR, false-discovery rate. **e** The heatmap depicts differentially expressed ISGs in EO771 cells treated with vehicle or 12-HETE. Numbers are log_2_-transformed. **f** qRT-PCR analysis of indicated ISGs in indicated cells treated with vehicle or 12-HETE. IFNα (**g**) and IFNβ (**h**) levels in the media of indicated cells treated with vehicle or 12-HETE. **i** Representative Western blots (left) and quantitation (right) of indicated protein levels in EO771 cells transfected with indicated sgRNA plasmids. Representative bioluminescent images (**j**) and quantitation (**k**) of lung metastases in wild-type and *Alox12*^*fl/fl*^;*Chst4-CreERT2* mice injected with the indicated tumour cells. **l** The quantitation of immunohistochemistry staining for CD8α and NK1.1 in lung metastases of wild-type and *Alox12*^*fl/fl*^;*Chst4-CreERT2* mice inoculated with indicated cells. Each dot represents data pooled from 5 distinct areas from a slice and 3 slices per mouse. *n* = 3 independent experiments (**a**, **e**, **i**); *n* = 4 independent experiments (**f**–**h**); *n* = 6 mice per group (**k**, **l**). Mean ± SD (**a**, **f**–**i**, **k**, **l**). *P*-values were calculated by two-tailed unpaired *t*-test (**a**, **f**–**i**) and one-way ANOVA with Tukey’s multiple comparisons test (**k**, **l**). n.d. not detected.

To explore how 12-HETE promotes immune evasion of tumour cells, we performed total RNA-sequencing (RNA-seq) analysis and detected a greater abundance of A-to-I editing events in EO771 cells after 12-HETE treatment (Fig. 5a). Consistent with the results of previous studies^40,70^, the vast majority of editing events were detected in 3′ untranslated regions (UTRs), intergenic regions and intronic regions (Fig. 5b). Importantly, about 70% of these sites were mapped to repeat elements, particularly short interspersed nuclear elements (SINEs), long interspersed nuclear elements (LINEs) and long-terminal repeats (LTRs) (Fig. 5c), which often form dsRNA^29^. In addition, gene set enrichment analysis (GSEA) revealed that 12-HETE inhibited the IFN response pathway (Fig. 5d, e). qRT-PCR and ELISA confirmed that 12-HETE suppressed the expression of IFN-stimulated genes (ISGs) (Fig. 5f) and the secretion of IFNα and IFNβ in multiple cell lines (Fig. 5g, h). A-to-I editing of endogenous dsRNA prevents activation of RNA sensors MDA5/ZBP1-dependent IFN response^32,35,37–39^. To investigate whether A-to-I editing induced by 12-HETE suppresses the activation of the MDA5/ZBP1-dependent IFN response pathway and tumour immunogenicity by reducing the recognition of dsRNA, we knocked out *Ifih1* (MDA5) and *Zbp1* (ZBP1) (Fig. 5i). We observed that the deletion of both MDA5 and ZBP1 abolished the metastasis-suppressing effect in the HEV-conditional *Alox12* knockout mice (Fig. 5j, k) and reversed the elevated CD8⁺ T and NK cell infiltration in the lung metastases of HEV-conditional *Alox12* knockout mice (Fig. 5l). It is well documented that both MDA5 and ZBP1 are IFN-stimulated genes (ISGs), with their expression regulated by IFNs^71,72^. To investigate how 12-HETE modulates the RNA sensor pathways mediated by MDA5 and ZBP1, we examined their protein levels and subcellular localisation in EO771 cells following 12-HETE treatment. Immunofluorescence analysis revealed reduced colocalisation of MDA5 or ZBP1 with dsRNA at 24 h, suggesting impaired dsRNA recognition (Supplementary Fig. 9a, b). By 72 h, total MDA5 and ZBP1 protein levels were also decreased (Supplementary Fig. 9a, b). These results indicate that 12-HETE initially promotes RNA editing to disrupt dsRNA integrity and prevent activation of MDA5/ZBP1-mediated IFN response. The resulting decline in IFN production subsequently suppresses MDA5 and ZBP1 expression, ultimately reducing tumour cell immunogenicity.

### 12-HETE enhances ADAR1 p150-dsRNA liquid-liquid phase separation

It has been reported that 12-HETE may act via a putative G_i/o_-coupled G-protein-coupled receptor (GPCR)^73^. However, ISG suppression was not affected by the G_i/o_ protein inhibitor pertussis toxin (PTX), indicating that this effect is G_i/o_-coupled GPCR-independent (Supplementary Fig. 10a). ADAR1, which is the predominant enzyme mediating the editing of these repeats, prevents the recognition of dsRNA by the dsRNA sensor-ISG pathway^31,74^. Our major finding is that 12-HETE promotes immune evasion of tumour cells by inhibiting the ISG expression and the IFN response pathway in tumour cells. Importantly, a genome-wide CRISPR screen in diverse mouse cancer cell lines co-cultured with cytotoxic T lymphocytes (CTLs) identified *Ptpn2*, *Socs1* and *Adar1* as key negative regulators of the interferon response that enable CTL evasion^75^. To investigate whether ADAR1 accounts for our observation, we evaluated the effect of 12-HETE on cancer cells with or without *Ptpn2*, *Socs1* and *Adar1* knockout, which contributes to the immune evasion of tumour cells by inhibiting the IFN response pathway in tumour cells. Consistently, deletion of *Adar1*, rather than other regulators, rescued ISG expression (Supplementary Fig. 10b–d), confirming that the effect of 12-HETE on immune evasion is dependent on ADAR1.

**Fig. 6:**
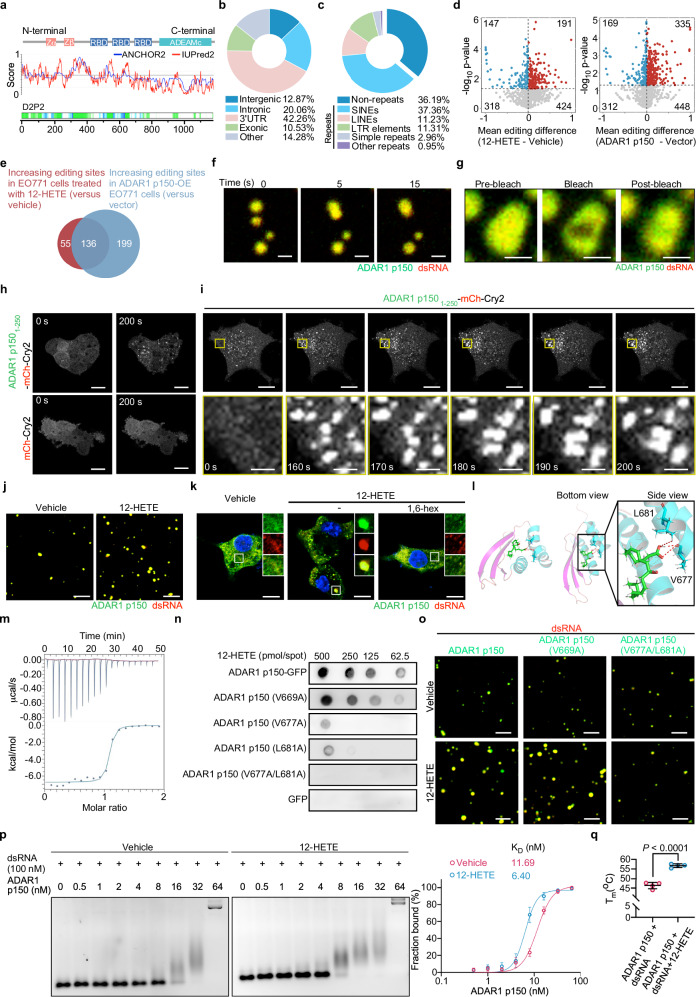
12-HETE enhances ADAR1 p150-dsRNA liquid-liquid phase separation. **a** Top, domain structure of ADAR1 p150. Bottom, predictions of IDRs by ANCHOR2, IUPred2 algorithms (https://iupred2a.elte.hu/) and D2P2 algorithms (https://d2p2.pro/). **b**, **c** Pie chart showing the genomic distribution of A-to-I RNA editing sites from EO771 cells with or without ADAR1 p150 overexpression. **d** Volcano plot showing differential A-to-I editing in EO771 cells with or without 12-HETE treatment and with or without ADAR1 p150 overexpression. **e** Venn diagram showing overlap of increased editing sites between 12-HETE-treated and ADAR p150-overexpressing EO771 cells. **f** Real-time fluorescence imaging of ADAR1 p150-dsRNA droplets fusion. Scale bars, 2 μm. **g** Representative images of ADAR1 p150-dsRNA droplets from a FRAP experiment. Scale bars, 1 μm. Quantitation in Supplementary Fig. 11f. **h**, **i**, HEK293T cells expressing an optoIDR construct (ADAR1 p150_1-250_-mCherry-Cry2) were subjected to 488 nm laser excitation every 2 s. Cells expressing mCherry-Cry2 were used as controls. Scale bars, 10 μm (main panels) and 2 μm (magnified regions). Quantitation in Supplementary Fig. 11g. **j** Representative images of droplets formed by 100 nM ADAR1 p150-GFP and 100 nM Cy5-dsRNA with or without 100 nM 12-HETE. Scale bars, 5 μm. Quantitation in Supplementary Fig. 11h. **k** Representative images of GFP-tagged ADAR1 p150 and dsRNA in EO771 cells with the indicated treatment. Scale bars, 10 μm. Quantitation in Supplementary Fig. 11j. Structural and biochemical analysis of ADAR1 p150 interaction with 12-HETE, including docking prediction (**l**), ITC binding analysis (**m**) and lipid-protein overlay assay (**n**). Quantitation in Supplementary Fig. 11l. **o** Representative images of droplets formed by 100 nM indicated recombinant proteins and 100 nM Cy5-dsRNA in the presence or absence of 100 nM 12-HETE. Scale bars, 5 μm. Quantitation in Supplementary Fig. 11m. EMSA analysis (**p**) and thermal shift assay (**q**) of the binding of ADAR1 p150 to dsRNA in the absence or presence of 12-HETE. *T*_m_, melting temperature. *n* = 2 independent experiments (**d**); *n* = 3 independent experiments (**f**, **p**); *n* = 4 independent experiments (**q**). Mean ± SD (**p**, **q**). *P*-values were calculated by two-sided Welch two-sample *t*-test (**d**) and two-tailed unpaired *t*-test (**q**).

However, no appreciable difference in the protein levels of either isoform of ADAR1 (p150 and p110) was observed in tumour cells after 12-HETE treatment (Supplementary Fig. 11a). Emerging evidence shows that LLPS can concentrate local reactants and enzymes, which enhances post-transcriptional modification of RNA by changing thermodynamics without total protein level changes^19,76,77^. Although whether ADAR1 can undergo LLPS with dsRNA is elusive, we identified intrinsically disordered regions (IDRs) enriched in the N-terminal section of ADAR1 p150 (Fig. 6a), which may drive LLPS. Interestingly, the editing activity of ADAR1 p150 was reported to be greater than that of ADAR1 p110 due to the absence of the N-terminal section of ADAR1 p150 in the ADAR1 p110 protein^78^. Furthermore, we observed that the vast majority of editing events occurred in the same locations of dsRNA in EO771 cells treated with 12-HETE as in those with ADAR1 p150 overexpression (Figs. 5b, c and 6b, c). Moreover, 71.2% of the editing sites with increased editing levels in the EO771 cells treated with 12-HETE overlapped with those in the EO771 cells with ADAR1 p150 overexpression (Fig. 6d, e). These data suggested that 12-HETE may promote the A-to-I editing of tumour cells by regulating the LLPS of ADAR1 p150 rather than adjusting the expression level of ADAR1 p150. To determine whether ADAR1 p150 undergoes LLPS with dsRNA, we engineered three ADAR1 p150-GFP constructs (Supplementary Fig. 11b). The full-length ADAR1 p150 and the truncated construct (p150_1-250_) containing the N-terminal section had similar ability to form liquid droplets. In contrast, the truncated construct (p150_251-1178_), which contains only the C-terminal section and is similar to ADAR1 p110, failed to generate liquid droplets (Supplementary Fig. 11c, d). Moreover, we found that ADAR1 p150 and dsRNA underwent LLPS in a concentration-dependent manner (Supplementary Fig. 11e). The droplets formed by ADAR1 p150 and dsRNA fused into large ones over time (Fig. 6f). Fluorescence recovery after photobleaching (FRAP) experiments showed that the fluorescence of ADAR1 p150 and dsRNA was efficiently recovered after photobleaching (Fig. 6g and Supplementary Fig. 11f). In addition, we applied an optogenetic platform that can dynamically modulate phase separation by using Cry2, a self-associating protein, after exposure to blue light^79^. Upon expressing mCherry-labelled Cry2 alone (mCh-Cry2) or mCherry-labelled Cry2 fused with IDR (ADAR1 p150_1-250_-mCh-Cry2) in HEK293T cells, we found that ADAR1 p150_1-250_-mCh-Cry2 rather than mCh-Cry2 showed apparent clustering, indicating that the N-terminal IDR is required for phase separation of ADAR1 p150 in cells (Fig. 6h, i and Supplementary Fig. 11g). Collectively, our data indicate that ADAR1 p150 and dsRNA can undergo LLPS.

To determine the effect of 12-HETE on ADAR1 p150-dsRNA LLPS, we incubated ADAR1 p150-GFP and Cy5-dsRNA with 100 nM 12-HETE. 12-HETE significantly enhanced ADAR1 p150-dsRNA LLPS in vitro at low concentrations of ADAR1 p150 and dsRNA (Fig. 6j and Supplementary Fig. 11h, i). In cells, GFP-tagged ADAR1 p150 formed more puncta with dsRNA detected by J2 antibody upon 12-HETE treatment, and this effect was abolished by the addition of 1,6-hexanediol, a LLPS inhibitor (Fig. 6k and Supplementary Fig. 11j). Collectively, these results indicate that 12-HETE promotes ADAR1 p150-dsRNA LLPS.

We next investigated how 12-HETE promotes the LLPS of ADAR1 p150 and dsRNA. Analysis of previously published data^80^ showed that a probe of arachidonic acid, the precursor of 12-HETE, can bind to YLNTNPVGGLLEYAR, which locates in the third double-stranded RNA-binding domain (dsRBD3) of ADAR1 p150 and is 100% conserved between mouse and human (Supplementary Fig. 11k). Molecular dynamics simulations using GROMACS-2020 showed that 12-HETE can be docked to the helices of dsRBD3 of ADAR1 p150 and the acidic headgroup formed intramolecular hydrogen bonds with residues V677 and L681 in the YLNTNPVGGLLEYAR domain (Fig. 6l), raising the possibility that 12-HETE promotes LLPS of ADAR1 p150 and dsRNA by binding to ADAR1 p150. To validate that 12-HETE directly interacts with ADAR1 p150, we used isothermal titration calorimetry (ITC) to measure the affinity and stoichiometry of 12-HETE binding to ADAR1 p150. The results revealed that 12-HETE bound to ADAR1 p150 with a stoichiometry of 1:1 and a *K*_D_ of 72.6 ± 24.9 nM (Fig. 6m). The lipid-protein overlay assay further confirmed that 12-HETE bound to ADAR1 p150 (Fig. 6n and Supplementary Fig. 11l). In consistence with the results of the molecular dynamics simulations, alanine mutations at V677 (V677A) and L681 (L681A) rather than the alanine mutation at V669 (V669A) disrupted the binding of 12-HETE to ADAR1 p150 (Fig. 6n and Supplementary Fig. 11l). Furthermore, an in vitro phase separation assay showed that the V677A/L681A mutation had no appreciable effect on the LLPS of ADAR1 p150 and dsRNA, whereas the promoting effect of 12-HETE on ADAR1 p150-dsRNA LLPS was abolished (Fig. 6o and Supplementary Fig. 11m). Ligands modulate the driving forces for LLPS through a mechanism known as polyphasic linkage^81^. Consistent with previous studies^82^, there is a dose-dependent enhancement in the driving forces for ADAR1 p150-dsRNA LLPS in the presence of 12-HETE (Supplementary Fig. 11i), which indicates that 12-HETE may promote the interaction between dsRNA and ADAR1 p150. Indeed, the results of electrophoretic mobility shift assay (EMSA) revealed that 12-HETE increased the RNA-binding affinity of ADAR1 p150 (Fig. 6p). Moreover, a thermal shift assay showed that 12-HETE increased the melting temperature of the ADAR1 p150-dsRNA complex, suggesting that 12-HETE stabilised the ADAR1 p150-dsRNA complex (Fig. 6q). Collectively, we propose that 12-HETE promotes ADAR1 p150-dsRNA LLPS through polyphasic linkage^81^, in which ligand enrichment within condensates decreases the saturation threshold of ADAR1 p150.

### ADAR1 p150-dsRNA LLPS induced by 12-HETE promotes A-to-I RNA editing

**Fig. 7:**
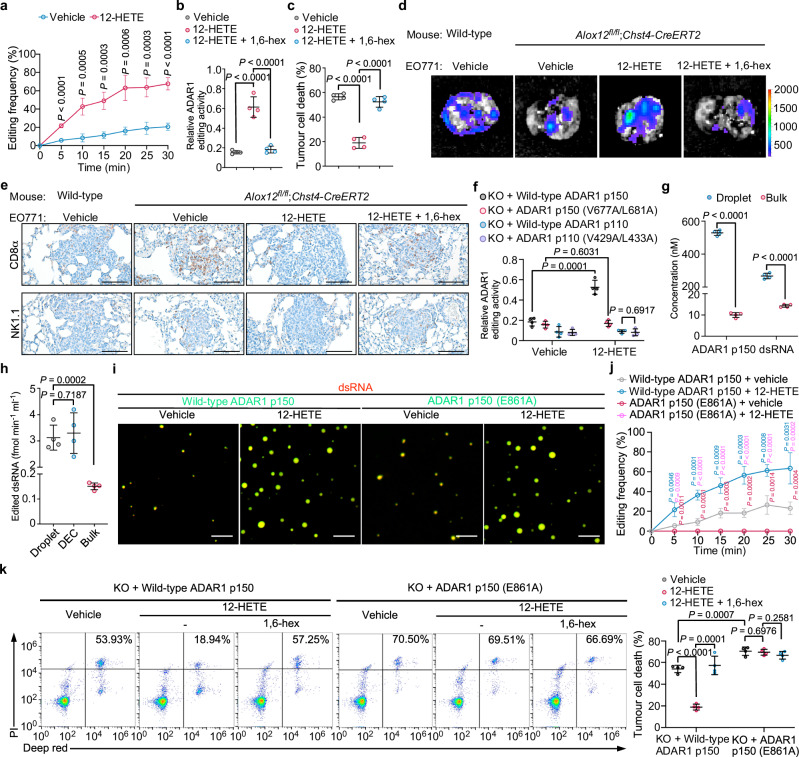
ADAR1 p150-dsRNA liquid-liquid phase separation induced by 12-HETE promotes A-to-I RNA editing. **a** Recombinant ADAR1 p150 was mixed with dsRNA in the presence or absence of 12-HETE, and the fraction of edited dsRNA was quantified. **b** ADAR1 editing activity in EO771 cells with indicated treatment was measured as the ratio between luminescence from Nano luciferase/Firefly luciferase. **c** Quantitation for CD8^+^ T cell-mediated cytotoxicity to EO771 cells with indicated treatment. Representative bioluminescent images of lungs (**d**) and immunohistochemistry staining for CD8α and NK1.1 in the lung metastases (**e**) from the indicated EO771-bearing mouse with the indicated treatment. Scale bars, 100 μm. Quantitation in the Supplementary Fig. 12a, b. **f** ADAR1 editing activity in *Adar1*-knockout EO771 cells expressing indicated ADAR1 variants with or without 12-HETE treatment was measured as the ratio between luminescence from Nano luciferase/Firefly luciferase. Quantitation of ADAR1 p150 and dsRNA concentrations in indicated phases (**g**) and volume-normalised editing rate in indicated phases and droplets equivalent concentration (DEC) (**h**). **i** Representative images of droplets formed by 100 nM Cy5-dsRNA with 100 nM wild-type ADAR1 p150-GFP or mutant ADAR1 p150-GFP (E861A) with or without 100 nM 12-HETE. Scale bars, 5 μm. Quantitation in the Supplementary Fig. 12j. **j** Indicated recombinant ADAR1 p150 proteins were mixed with dsRNA in the presence or absence of 12-HETE, and the fraction of edited dsRNA was quantified. The blue *P*-values for (wild-type ADAR1 p150 + 12-HETE) versus (wild-type ADAR1 p150 + vehicle), the violet *P*-values for (ADAR1 p150 (E861A) + 12-HETE) versus (wild-type ADAR1 p150 + 12-HETE) and the red *P*-values for (ADAR1 p150 (E861A) + vehicle) versus (wild-type ADAR1 p150 + vehicle). **k** Representative flow cytometry plots (left) and quantitation (right) for CD8^+^ T cell-mediated cytotoxicity to indicated EO771 cells with indicated treatment. Unstimulated T cell control in Supplementary Fig. 12k. *n* = 4 independent experiments (**a**–**c**, **f**–**h**, **j**, **k**). Mean ± SD (**a**–**c**, **f**–**h**, **j**, **k**). *P*-values were calculated by two-tailed unpaired *t*-test (**a**, **f**, **g**, **j**, **k**) and one-way ANOVA with Tukey’s multiple comparisons test (**b**, **c**, **h**).

As 12-HETE enhances both A-to-I RNA editing (Fig. 5) and ADAR1 p150-dsRNA LLPS (Fig. 6), we investigated whether ADAR1 p150-dsRNA LLPS is indispensable for A-to-I RNA editing induced by 12-HETE. In vitro editing assay showed that 12-HETE markedly increased A-to-I editing of dsRNA in the presence of ADAR1 p150 (Fig. 7a). Corroborating our in vitro results, a dual-luciferase reporter assay^83^ showed that addition of 12-HETE enhanced dsRNA editing activity within tumour cells (Fig. 7b). Moreover, 12-HETE pretreatment increased resistance of tumour cells to the T cell-mediated cytotoxicity (Fig. 7c). In vivo, 12-HETE treatment of tumour cells diminished the inhibition of metastases (Fig. 7d and Supplementary Fig. 12a) and the increase in CD8^+^ T cells and NK cells infiltration in the lung metastases in the *Alox12*^*fl/fl*^;*Chst4-CreERT2* mice compared with their wild-type littermates (Fig. 7e and Supplementary Fig. 12b). Importantly, these effects were abolished by the addition of 1,6-hexanediol, a LLPS inhibitor, during 12-HETE treatment (Fig. 7b–e and Supplementary Fig. 12a, b), which suggests that 12-HETE increases A-to-I RNA editing and reduces immunogenicity of tumour cells by promoting ADAR1 p150-dsRNA LLPS.

To further specifically validate that 12-HETE enhances ADAR1 p150-RNA LLPS to promote A-to-I RNA editing and reduce immunogenicity of tumour cells, we knocked out endogenous ADAR1 in EO771 cells, followed by reintroduction of wild-type ADAR1 p150, mutant ADAR1 p150 (V677A/L681A), wild-type ADAR1 p110 and mutant ADAR1 p110 (V429A/L433A). The promoting effect of 12-HETE on A-to-I editing was dramatically reduced in the KO + ADAR1 p150 (V677A/L681A) EO771 cells compared with KO + WT ADAR1 p150 EO771 cells and was not observed in either the KO + WT ADAR1 p110 or KO + ADAR1 p110 (V429A/L433A) EO771 cells (Fig. 7f). Consistently, 12-HETE increased A-to-I editing of dsRNA by recombinant ADAR1 p150 protein but not other ADAR1 protein variants in vitro (Supplementary Fig. 12c). In addition, we evaluated the type-I IFN expression and T cell cytotoxicity in KO + WT ADAR1 p150, KO + ADAR1 p150 (V677A/L681A), KO + WT ADAR1 p110 and KO + ADAR1 p110 (V429A/L433A) EO771 cells treated with or without 12-HETE. The results revealed that the inhibiting effect of 12-HETE on type-I IFN expression and immunogenicity of tumour cells was dramatically reduced in KO + ADAR1 p150 (V677A/L681A) EO771 cells compared with KO + WT ADAR1 p150 EO771 cells and was not observed in both KO + WT ADAR1 p110 and KO + ADAR1 p110 (V429A/L433A) EO771 cells (Supplementary Fig. 12d, e). In vivo, HEV-conditional *Alox12* knockout significantly suppressed lung metastases (Supplementary Fig. 12f) and enhanced CD8^+^ T and NK cells infiltration in the lung (Supplementary Fig. 12g) of KO + WT ADAR1 p150 EO771-bearing mice. Strikingly, these effects were not observed in KO + ADAR1 p150 (V677A/L681A) EO771-bearing mice (Supplementary Fig. 12f, g), indicating that disrupting 12-HETE-induced LLPS effectively abolishes its pro-metastatic and immunosuppressive effects. In contrast, there was no significant difference in metastatic burden or immune cell infiltration between the KO + WT ADAR1 p110 and KO + ADAR1 p110 (V429A/L433A) groups (Supplementary Fig. 12f, g), further supporting the isoform-specific role of ADAR1 p150 in LLPS. Collectively, these results indicate that although both ADAR1 p150 and ADAR1 p110 contain the binding site of 12-HETE in the dsRBD3, the promoting effect of HEV-derived 12-HETE on A-to-I editing and the inhibiting effect of 12-HETE on type-I IFN expression and immunogenicity of tumour cells are dependent on the LLPS driven by the N-terminal section of ADAR1 p150.

We next investigate how ADAR1-dsRNA LLPS promotes A-to-I RNA editing. Condensates can accelerate enzymatic reactions by elevating local enzyme and substrate concentrations without an encapsulating membrane^84,85^. Consistently, we found higher concentrations of ADAR1 p150 and dsRNA in the droplets compared with the bulk phases (Fig. 7g and Supplementary Fig. 12h). Moreover, we carried out reactions at the concentrations measured within the droplets with dsRNA (270 nM) and truncated ADAR1 p150 (residues 251–1178) (530 nM), which lost the ability to undergo LLPS. In these droplet equivalent concentration (DEC) reactions without phase separation^84,86^, the dsRNA editing rate was not significantly different from that in the droplets (Fig. 7h and Supplementary Fig. 12i). Collectively, these data indicate that LLPS promotes A-to-I editing by increasing the concentrations of ADAR1 p150 and dsRNA.

To further specifically validate that the increased A-to-I RNA editing mediated by ADAR1 p150 is required for the reduced immunogenicity by 12-HETE, we constructed an editing-deficient, dsRNA-binding-intact ADAR1 p150 mutant (E861A)^32^. No appreciable difference in the formation of wild-type ADAR1 p150-dsRNA condensates and ADAR1 p150 (E861A)-dsRNA condensates in the absence or presence of 12-HETE was observed (Fig. 7i and Supplementary Fig. 12j). In vitro editing assays showed that ADAR1 p150 (E861A) had no A-to-I editing activity, and 12-HETE had no effect on editing activity of ADAR1 p150 (E861A) (Fig. 7j). These data indicated that the E861A mutation abrogates the A-to-I editing capacity of ADAR1 p150, whereas it had no effect on ADAR1 p150-dsRNA LLPS. Next, we knocked out ADAR1 p150 in EO771 cells, following by reintroduction of the wild-type construct (KO + WT ADAR1 p150) or the ADAR1 p150 (E861A) (KO + ADAR1 p150 (E861A)). The KO + ADAR1 p150 (E861A) EO771 cells were more sensitive to T cell-mediated cytotoxicity compared with KO + WT ADAR1 p150 EO771 cells (Fig. 7k and Supplementary Fig. 12k). 12-HETE pretreatment increased resistance of KO + WT ADAR1 p150 EO771 cells to T cell-mediated cytotoxicity, and this effect was abolished by the addition of 1,6-hexanediol during 12-HETE treatment (Fig. 7k and Supplementary Fig. 12k). Importantly, these effects were abrogated in KO + ADAR1 p150 (E861A) EO771 cells (Fig. 7k and Supplementary Fig. 12k). In vivo, HEV-specific *Alox12* deletion significantly suppressed lung metastasis (Supplementary Fig. 12l) and markedly enhanced infiltration of CD8⁺ T cells and NK cells in the lung (Supplementary Fig. 12m) of KO + WT ADAR1 p150 tumours bearing mice. In contrast, these effects were abrogated in mice bearing KO + ADAR1 p150 (E861A) tumours, despite intact LLPS capability (Supplementary Fig. 12l, m). These findings demonstrate that 12-HETE-induced LLPS of ADAR1 p150 is not sufficient by itself to drive immune evasion. Instead, A-to-I RNA editing mediated by the catalytic deaminase domain is indispensable, serving as a prerequisite for suppression of MDA5/ZBP1-driven interferon signalling. In summary, these results suggest that 12-HETE facilitates ADAR1 p150-dsRNA LLPS and subsequently promotes A-to-I editing, leading to reduced immunogenicity of tumour cells by suppressing the MDA5/ZBP1-ISG pathway.

## Discussion

In breast cancer, the dissemination route involving the LNs was associated with a worse prognosis^87^. However, there remains considerable debate regarding the clinical significance of LN metastases^6,11^. Although evidence from human and mouse studies indicates that LN metastases could seed distant metastases^88–91^, LN removal does little to improve long-term outcomes in early-stage breast cancer^92^. However, this does not necessarily mean that LN metastases do not have clinical significance, as breast cancer is considered a systemic disease, in which disseminated cancer cells from LN metastases may enter the bloodstream prior to their clinical detection^93^. More importantly, cancer cells from LN metastases exhibit higher survival ability and immune evasion properties^8,9,94^. Recent evidence has shown that HEVs offer an additional portal for circulating cancer cells in mice^6,7^. We provide clear evidence that HEV involvement is an independent prognostic marker to further stratify the prognosis of patients with LN-positive breast cancer. HEVs are anatomically unique venules in lymphoid tissues^12–18^. Mechanistically, we highlight a functional difference between HEVs and non-HEV blood vessels in shaping the future fates of migrating tumour cells. The theory that different routes have distinctive impacts on metastatic cells was corroborated by a previous study showing that lymph fluid in LNs can protect tumour cells from ferroptosis via an immunity-independent mechanism^8^. This report and our study demonstrate different mechanisms at different stages of LN metastases dissemination, which are triggered by different fatty acids. Interestingly, HEVs can be found not only in LNs, but also in tertiary lymphoid structures (TLSs) in tumours. Given the unexplained association of TLSs with poor prognosis^95–97^, it will be important to determine whether HEVs in TLSs play a role in tumour dissemination or modulate therapeutic responses. Previous studies indicate that mature TLSs containing PNAd⁺ HEVs are more frequently observed in cancer patients after chemotherapy^98–100^, especially in patients with complete remission after chemotherapy^101^. Thus, the potential roles of HEVs within TLSs, particularly their ALOX12 expression, in tumour progression remain unclear and warrant detailed mechanistic investigation in future studies.

HEVs exhibit remarkable phenotypic diversity, heterogeneity, and plasticity, undergoing extensive remodelling in inflamed LNs and tumour-draining LNs^13,14,102–104^. However, the direct interactions between HECs and tumour cells in metastatic LNs remain poorly understood. In this study, we identified that ALOX12 expression in tumour-associated HEVs is dynamically induced by tumour-derived SEMA3C in metastatic LNs. Although HECs are challenging to isolate and thus underrepresented in scRNA-seq datasets, a common limitation in lymph node endothelial studies^103^, our findings are supported by multiple complementary approaches. Notably, ALOX12 upregulation is not uniform across all HEVs but is selectively enriched in tumour-associated HEVs, which represent the functionally relevant subset. While a small fraction of non-HEV BECs also exhibit low-level ALOX12 expression^105^, HEVs are the major route for tumour cells to enter circulation in LNs^6^ and represent the predominant and functionally indispensable source of ALOX12 in metastatic LNs. Mechanistically, the preferential induction of ALOX12 in tumour-associated HEVs is consistent with the selective expression of the SEMA3C co-receptor PlexinA1 in HEVs and higher levels of NRP2 in HECs compared with non-HEV BECs. Moreover, we used HEV-conditional knockout mice to confirm the essential role of HEV-derived ALOX12 in the immune evasion of LN tumour escape in vivo. Together, these findings reveal a spatially restricted yet functionally critical feedback loop between metastatic tumour cells and the LN HECs. However, mechanistic dissection of the downstream signalling pathways remains challenging, as primary HECs cannot be stably cultured or maintained ex vivo^106^. This technical limitation precludes conventional in vitro manipulation and signalling assays. Therefore, future studies employing conditional genetic mouse models will be essential to further elucidate the molecular cascade by which tumour-derived SEMA3C activates the NRP2/PlexinA1-ALOX12 axis in tumour-associated HEVs within metastatic lymph nodes.

Despite the existence of small-molecule ALOX12 inhibitors such as Baicalein and ML355, their clinical translation has been limited^63,107,108^. Our study identifies SEMA3C-NRP2/PlexinA1 as an upstream regulatory axis that drives ALOX12 expression in HEVs within metastatic LNs. This finding suggests that targeting this ligand-receptor pair, for example, through neutralising antibodies against SEMA3C or PlexinA1, may represent a more feasible and therapeutically actionable approach to modulate ALOX12 activity in a tissue- and context-specific manner.

Of note, ALOX15, another lipoxygenase family member, was reported to be expressed by some human breast cancer cells and propagate lymphatic metastasis via 12-HETE^61^. However, the mouse model used in the previous study is an immunocompromised SCID mouse, which may not show the immune regulatory function of 12-HETE described in our study. By comparison, we did not capture the circular chemorepellent-induced defect effects reported in the previous study, probably because we used a much lower dose (100 nM) of 12-HETE compared with the dose (1 μM) used in the previous study. 12-HETE may have pleiotropic effects across multiple cell types (tumour cells vs. HECs) and in different tumour progression stage- and organ-contexts (metastasis from primary tumours to LNs vs. metastasis from LNs to distant organs), which is very common in tumour cell biology^53,109–112^. We believe that these two studies are complementary, examining different, but equally important, roles of ALOX12/12-HETE in different cell types. The circular chemorepellent-induced defect effects of cancer cell-derived 12-HETE facilitate the entry of tumour cells into lymphatic vessels at primary sites and their spread to LNs. Whereas HEV-derived ALOX12/12-HETE acts downstream of this process, which empowers tumour cells to settle in distant organs by eliciting a weaker immune response after entering the circulation via HEVs in LNs. ALOX12 has also been reported to be essential for p53-mediated tumour suppression through a distinct ferroptosis pathway^113^. Our findings provide an additional level of understanding of ALOX12 biology by demonstrating that elevated ALOX12 expression in HEVs contributes to immune evasion and promotes metastatic progression. Importantly, the mechanism that we identified depends on its enzymatic product, 12-HETE. Previous studies have shown that 12-HETE is involved in broader tumour-promoting mechanisms^60,114,115^. Thus, our results do not contradict existing literature but instead highlight the substrate- and context-dependent roles of ALOX12.

Metastasis is an inefficient process. Leaving the immunosuppressive primary tumour microenvironment, most circulating tumour cells are annihilated by host factors, such as immune cells in distant organs^116–120^. Endogenous IFNs upregulate many immune-interacting molecules, such as MHCI and stress ligands, in tumour cells, making them more sensitive to tumoricidal effects of immune cells^121,122^. In addition, IFNs act directly on effector cells as a third signal to increase their cytotoxic functions^123,124^. Our data suggest that HEV-emigrant cancer cells evade the killing of effector cells in distant organs because less tumour-derived IFNs directly reduce their tumoricidal ability. In addition, HEV-emigrant cells in the lungs also initiate a weaker immune response in LNs draining the lungs. Therefore, HEVs shield the “Achilles heel” immune vulnerability of circulating tumour escapees, because disseminated tumour cells exposed to 12-HETE produce less endogenous IFNs and therefore elicit a weaker anti-tumour immune response in distant organs.

It has been well documented that A-to-I editing reduces the recognition of dsRNA, which suppresses the IFN response pathway and tumour immunogenicity^29,31,32,35,37–40,74^. ADAR1 is the primary enzyme that catalyses the chemical conversion of adenosines to inosines in dsRNA^125–129^. Numerous studies using independent genetic evidence from various cancer cell types have demonstrated that perturbation of ADAR1 reduces cancer cell viability in vitro and sensitises tumours to immunotherapy in vivo by restoring the dsRNA sensing pathway^31,40–42^, highlighting ADAR1 as a promising therapeutic target. In addition, a novel small-molecule inhibitor, ZYS-1, has been developed, demonstrating strong anti-tumour activity together with an acceptable safety profile. Collectively, these findings indicate that ADAR1 is druggable and that ADAR1 inhibitors may hold broad therapeutic potential across diverse cancers^130^. Our study adds mechanistic depth to this concept by elucidating the role of LLPS in modulating ADAR1 activity. The enzymatic reaction rates depend on the concentrations of the reactants. LLPS speeds up enzymatic reaction rates by enriching the reaction contents. For instance, LLPS of cyclic GMP-AMP synthase (cGAS)-DNA promotes cGAMP synthesis by locally enriching ATP, GTP, and cGAS^131^. Although IDR can be identified in ADAR1 p150, whether ADAR1 p150 can undergo LLPS with dsRNA and its biological significance is elusive. Here, we uncovered that ADAR1 p150-dsRNA condensates enhanced by 12-HETE dramatically increase the concentration of compartmentalised ADAR1 p150 and dsRNA, leading to highly efficient A-to-I editing of dsRNA. Consistently, A-to-I editing levels rather than ADAR1 levels correlate with patient survival in breast cancer, liver cancer and head and neck cancer^132–134^. Our preliminary data suggest that 12-HETE binds to ADAR1 p150 and may promote ADAR1 p150-dsRNA phase separation through a mechanism known as polyphasic linkage^81^. This observation raises the possibility that 12-HETE facilitates LLPS of ADAR1 p150 in a ligand-dependent manner. To elucidate the molecular basis and mode of interaction between 12-HETE and ADAR1 p150, the future use of tools such as cryo-electron microscopy (cryo-EM) would be highly valuable.

Oncometabolites produced by upregulated metabolic enzymes could lead to tumour progression by altering genome-wide histone and DNA methylation beyond the conventional roles in metabolic processes^135^. Palmitic acid has prolonged effects on tumour cells by promoting deposition of histone H3 lysine 4 trimethylation, even when this oncometabolite no longer exists^136^. Herein, the immunogenicity of HEV-emigrant cancer cells was reduced by 12-HETE produced by HECs highly expressing ALOX12. Mechanistically, 12-HETE directly interacts with ADAR1 p150 and promotes the LLPS of ADAR1 p150-dsRNA, leading to the enhancement of epigenetic A-to-I RNA editing. Irreversibly edited RNAs can persist in cells for up to 30 days^137,138^. Tumour cells can arrive in the lungs and trigger an immune response in mediastinal LNs within 72 h after they enter circulation^50^. Therefore, this study, together with others^136^, demonstrated that epigenetic reprogramming induced by short-term exposure to oncometabolites in special niches can provide a long-term survival advantage for disseminated tumour cells in distant organs.

Overall, we identified tumour involvement in HEVs as a biomarker to stratify the prognosis of LN-positive breast cancer patients and revealed a previously undefined role of ALOX12-derived metabolite 12-HETE in mediating epigenetic A-to-I RNA editing by acting as an endogenous inducer of ADAR1 p150-dsRNA LLPS, which is an attractive therapeutic target. Therefore, the route-endowed immune evasion that we uncover here can be leveraged to devise novel immunotherapies against tumour metastasis (Supplementary Fig. 13). Considering the critical involvement of HEVs in diverse diseases, including cancer, infection and autoimmune diseases, this finding serves as a foundation that other studies can build upon.

## Methods

### Human samples

Paraffin-embedded LN samples were collected from the Sun Yat-Sen Memorial Hospital, Sun Yat-Sen University (Guangzhou, China), the Affiliated Hospital of Guizhou Medical University (Guiyang, China) and the First Affiliated Hospital of Zhengzhou University (Zhengzhou, China). From 2008 to 2015, the records of all female patients with primary invasive breast cancer and at least one positive SLN obtained by SLN biopsy or at least one positive ALN obtained by ALN dissection without SLN biopsy were retrospectively reviewed at the Sun Yat-Sen Memorial Hospital. From 2010 to 2015, the records of all female patients with primary invasive breast cancer and at least one positive SLN were retrospectively reviewed at the Affiliated Hospital of Guizhou Medical University and the First Affiliated Hospital of Zhengzhou University. Patients with incomplete clinical information, unavailable pathological slides, another concurrent cancer, or recurrence/metastasis at diagnosis were excluded. In total, 559 SLN-positive (the SLN-positive cohort from the Sun Yat-Sen Memorial Hospital) and 457 ALN-positive (the ALN-positive cohort from the Sun Yat-Sen Memorial Hospital) patients from the Sun Yat-Sen Memorial Hospital, 207 SLN-positive patients from the Affiliated Hospital of Guizhou Medical University and 213 SLN-positive patients from the First Affiliated Hospital of Zhengzhou University were included.

Fresh tissues of breast tumour and LN were collected in the sterile surgery room from Sun Yat-Sen Memorial Hospital and transferred to sterile 15 ml conical tubes containing DMEM (Gibco, #11905065). Tissues were subsequently used for immunofluorescence analysis and primary endothelial cell isolation.

Informed consent was obtained from all patients, and all procedures were approved by the internal review and ethics boards of Sun Yat-Sen Memorial Hospital, the Affiliated Hospital of Guizhou Medical University and the First Affiliated Hospital of Zhengzhou University.

### Mice

All animal studies were approved by the Institutional Review Boards and Animal Care and Use Committees of Sun Yat-Sen University. Female mice were randomly selected and maintained under SPF conditions in individually ventilated cages. Humane endpoints were defined by animal discomfort and tumour size, with no mouse having a tumour size >2 cm in any dimension and abdominal distension (≥10% original body weight increase).

C57BL/6 and NOD/SCID mice (5-6 weeks old) were obtained from the Laboratory Animal Resource Center of Sun Yat-Sen University. To label HEV endothelial cells (HECs) by a genetic approach, we generated *Chst4-tdTomato* mice in which tdTomato expression is controlled by the HEV-restricted sulfotransferase gene, *Chst4*. To specifically trace tumour cells disseminating through HEVs, we generated a line of *R26-LSL-sLP-mCherry* mice (C57BL/6J-Gt (ROSA)26Sor^em1(CAG^^-^^loxp-STOP-loxp-Kozak-SP-TATK-mCherry-NLS-WPRE-pA)^) with expression of a secreted mCherry protein fused to a modified lipid-permeable TATk peptide (sLP-mCherry) under a stop codon. *Chst4-tdTomato*, *Chst4-CreERT2 R26-LSL-sLP-mCherry*, and *Alox12*^*fl/fl*^ strains were constructed using CRISPR/Cas9 technology at Shanghai Model Organisms Centre, Inc (Shanghai, China). *Chst4-CreERT2* mice were crossed to *R26-LSL-sLP-mCherry* mice to obtain conditional *Chst4-CreERT2*;*R26-LSL-sLP-mCherry* mice. *Chst4-CreERT2* mice were crossed to *Alox12*^*fl/fl*^ mice to obtain conditional knockout *Alox12*^*fl/fl*^;*Chst4-CreERT2* mice. Tail DNA was genotyped via PCR using designated primers (Supplementary Dataset 4) with Mighty Amp Genotyping Kit (TAKARA, #R074A), and PCR products were verified by sequencing. HEC-specific tdTomato and mCherry expression in *Chst4-tdTomato* mice was validated by immunofluorescence. Specific deletion of *Alox12* in HECs of *Alox12*^*fl/fl*^;*Chst4-CreERT2* mice was validated by immunofluorescence and qRT-PCR. *Chst4-tdTomato* and *Chst4-CreERT2* mice, maintained as hemizygotes, were healthy, fertile, and without obvious abnormalities. In addition, no appreciable difference in the size between *Chst4-tdTomato*, *Chst4-CreERT2* mice and wild-type littermates.

### Cell lines

The mouse breast cancer cell line EO771 was obtained from CH3 Biosystems. The mouse breast cancer cell line Py230, the murine melanoma cell line B16F10 and HEK293T were purchased from American Type Culture Collection (ATCC). Py230 cells were cultured in F12K medium (Gibco, #21-127-022) containing 5% foetal bovine serum (FBS) (Gibco, #10099-141), 0.1% serum extender (Corning, #355006) and 100 IU/ml penicillin/streptomycin (Thermo Fisher Scientific, #15140122). Other cell lines were maintained in DMEM (Gibco, #11905065) containing 10% FBS, 2 mM L-glutamine (Thermo Fisher Scientific, #A2916801) and 100 IU/ml penicillin/streptomycin. In some experiments, EO771 cells were treated for 48 h with vehicle (2% DMSO in saline), 100 nM 12-HETE (Cayman, #34570), 12-HETE with 3% 1,6-hexanediol (Sigma Aldrich, #240117) or 12-HETE with 150 ng/ml PTX (Cayman, #23221). All cell lines were cultured at 37 °C in a humidified incubator with 5% CO₂ and routinely tested negative for mycoplasma.

Stable GFP-EO771, Dendra2H2B-EO771, Dendra2H2B-Py230 and Dendra2H2B-B16F10 cell lines were transduced in the presence of 8 μg/ml polybrene (Beyotime, #C0351), using lentiviruses packaged by GenePharma Inc (Shanghai, China). GFP and Dendra2H2B lentivirus supernatant were added at a multiplicity of infection (MOI) of 10 for 6 h before replacement with fresh complete medium. Stable populations of GFP and Dendra2H2B-expressing tumour cells were selected by 1 μg/ml puromycin (Beyotime, #ST551), and subsequently enriched by FACS (BD Biosciences), with the top 20% cells expressing the highest level of GFP and Dendra2H2B collected for culture.

To generate the endogenously GFP-tagged ADAR1 p150 cell line, a monomeric enhanced GFP (mEGFP) sequence was cloned into the N-terminus of ADAR1 p150 using CRISPR/Cas9 technology in EO771 cells. The sequence that was targeted was 5′-TTGCCGGCACTATGTCTCAA-3′. Cas9-sgRNA plasmids were obtained from Genechem Company (Shanghai, China). EO771 cells were transfected with Lipofectamine 3000 (Thermo Fisher Scientific, #L3000001) following the manufacturer’s protocol, and the medium was replaced after 24 h. GFP-positive cells were isolated by FACS and confirmed by sequencing. The primer pairs that were used for genotyping were provided in Supplementary Dataset 4.

Cas9-sgRNA plasmids were obtained from ShangHai GeneBio Co., Ltd. *Sema3c*, *Nrg1*, *Tff2*, *Apoc4*, *Rbp4*, *Zbp1*, *Ifih1, Adar1, Socs1* or *Ptpn2* were deleted in EO771 cells by transient transfection with Cas9-sgRNA plasmids using Lipofectamine 3000. Puromycin (1 μg/ml) was added 48 h post-transfection. Knockout efficiency was confirmed by Western blotting after five days. Details of sgRNA sequences are listed in Supplementary Dataset 4.

To generated the KO + WT ADAR1 p150, KO + ADAR1 p150 (V677A/L681A), KO + WT ADAR1 p110, KO + ADAR1 p110 (V429A/L433A) and KO + ADAR1 p150 (E861A) EO771 cells, wild-type ADAR1 p150, wild-type ADAR1 p110, ADAR1 p150 (V677A/L681A), ADAR1 p110 (V429A/L433A) and ADAR1 p150 (E861A) were synthesised as gene fragments (Genechem), assembled into CMV-MCS-SV40-Neomycin vector (Genechem) digested with XhoI and KpnI and confirmed by DNA sequencing. Then wild-type ADAR1 p150, wild-type ADAR1 p110, ADAR1 p150 (V677A/L681A), ADAR1 p110 (V429A/L433A) and ADAR1 p150 (E861A) expression vectors were reintroduced to ADAR1-KO EO771 cell lines. Briefly, EO771 cells were transfected with Cas9-sgRNA plasmids using Lipofectamine 3000 to delete *Adar1*. Medium was changed after 24 h, and 1 μg/ml puromycin was added at 48 h after transfection. Following a 3–5 day recovery, knockout efficiency was validated by Western blot. Then wild-type ADAR1 p150, wild-type ADAR1 p110, ADAR1 p150 (V677A/L681A), ADAR1 p110 (V429A/L433A) and ADAR1 p150 (E861A) expression vectors were reintroduced to ADAR1-KO EO771 cell lines using Lipofectamine 3000. Selection was carried out using 400 µg/ml Geneticin (Thermo Fisher Scientific, #10131035) at 48 h after transfection. The re-expression of wild-type ADAR1 p150, wild-type ADAR1 p110, ADAR1 p150 (V677A/L681A), ADAR1 p110 (V429A/L433A) and ADAR1 p150 (E861A) was validated by Western blot.

### Survival data and deep learning analysis

The patient’s clinicopathological variables, including age, menopausal status, tumour size, histological grade, hormone receptor status, human epidermal growth factor receptor 2 (HER2) status, nodal status, lymphovascular invasion (LVI) and KI67 were analysed^139^. HEV involvement status was defined as positive if metastatic tumour cells were located on the HEV wall with adhesive, transendothelial and intravascular patterns in LNs. To improve diagnostic accuracy and efficiency, a diagnostic deep learning algorithm was developed to detect HEV involvement in the whole-slide images (WSIs)^140^. The WSIs were acquired by Axio Scan.Z1 (Zeiss) automatically with a 20× objective lens. A system of deep neural networks to detect the HEV involvement locations was developed using WSIs. The workflow is as follows:

#### Preprocessing

This step was taken to separate the HEVs and tumour lesions in the slides from the non-relevant background and standardise the tissue appearance. The ALN-positive cohort from Sun Yat-Sen Memorial Hospital (457 patients) was randomly split into training (*n* = 321), validation (*n* = 68), and testing (*n* = 68) sets. HEV and tumour regions were manually annotated in QuPath by two pathology-trained operators (X.W. and B.Z.), blinded to clinical data, with a strict verification process applied. Due to the large size of WSIs, only partial regions were annotated. At least eight representative regions per slide containing both tumour cells and HEVs were outlined. Within the selected regions, tumour cells and HEVs were manually annotated exhaustively with different colours. We selected a random subset of images with 1024 × 1024 resolution around the representative regions. In total, we obtained 3656 images from the training, validation and testing sets. Each image had a tumour region binary mask and an HEV region binary mask assigned with respective labels. Subsequently, the annotations were checked by two experienced pathologists (Y.Z. and Q.T.), and discrepancies were resolved by consensus discussion. Prior to algorithm implementation, we applied data augmentation to improve generalisability, including random rotation, scaling, flipping, modification of saturation, brightness and contrast. This strategy generates new training samples to enhance the diversity of data available for training models.

#### Deep learning framework

This step was taken to train the network to learn the features of HEVs and tumour lesions automatically. UNet++ is adopted to extract the tumour and HEV regions from the WSIs as our image segmentation network. The architecture includes an encoder backbone, a decoder module, and nested dense convolutions connecting the encoder and decoder feature maps^141^. The ResNeSt14d architecture was implemented as a backbone in the encoder and pretrained on a breast cancer histology image dataset online (https://www.kaggle.com/paultimothymooney/predict-idc-in-breast-cancer-histology-images). The UNet+ + networks were trained on the training set (*n* = 321) using the open-source Pytorch platforms. During training, images were cropped to 1024×1024 resolution and rotated randomly to augment our dataset. Our segmentation network was trained using a backpropagation algorithm that optimised the parameters across layers to get the most desired output when an input was given. All layers of the segmentation network were fine-tuned using cosine annealing warm restarts with the initial learning rate of 0.000001. The models restarted the learning rate after 5 epochs. The loss was calculated using a Tversky loss function and minimised using the RAdam optimiser. The batch size was set to 16, and the training process was iterated 200,000 iterations. The output layer produced a tumour probability map and an HEV probability map by classifying each pixel as tumour cells, HEVs or backgrounds. A pixel was identified as a tumour or HEV pixel if its probability of being tumour cells or HEVs was greater than 0.99. Connected components were extracted, and components smaller than 20 pixels were removed. Subsequently, based on the tumour and HEV detection result, the probability maps were post-processed to produce HEV involvement locations and scores. In addition, tumour lesion areas were calculated for evaluating the nodal tumour burden^140^.

Afterwards, we trained a classification model with a selection strategy to predict the presence of HEV involvement in WSIs with corresponding probability scores. The model was constructed using the ResNeSt14d architecture, which was pretrained as described above. During training, we applied the Cosine Annealing Warmup strategy with warmup steps of 5, a cycle step of 100, gamma of 0.9, a minimum learning rate of 0.0001, and an initial maximum learning rate of 0.01. The RAdam optimiser was employed with a weight decay of 0.0001, and training was run for 1000 epochs. To prevent overfitting during training, we set a drop rate of 0.5. All input images were resized to 512 × 512 × 3, with padding applied to maintain aspect ratio. During validation and testing, the location of candidate areas in WSIs was determined using the segmentation-based method as described above. The classification model was then used to provide confidence scores for each area. The confidence for each WSI was calculated by taking the top 1 areas’ confidence score. Finally, WSIs with confidence values greater than 0.65 were classified as the target WSIs.

At the end of training, the validation set (*n* = 68) was used to assess the segmentation and classification networks. For segmentation, 1024 × 1024 patches were generated as inputs, and performance was measured using the Jaccard index^142^ with a 0.99 threshold. Classification accuracy was evaluated at the patch level using ROC curves. To minimise bias from slide defects such as tissue damage or thickness, we resliced or manually identified high-quality slides for training. Moreover, staining bias is technically inevitable and one of the main sources of bias in deep learning for digital pathology, especially caused by tissue processing and staining procedures from different laboratories. To reduce staining bias in different datasets, we applied data augmentation to improve generalisability, including random rotation, scaling, flipping, modification of saturation, brightness and contrast. Insufficient training leads to underfitting, while excessive training causes overfitting and poor test performance. To balance this, training is stopped once validation performance declines, a strategy known as early stopping^143^.

### HEV involvement detection

The WSIs in the testing set (*n* = 68) were tested by our deep learning model to evaluate their ability to identify HEV involvement. The algorithm predicted slide HEV involvement labels, which were compared with the 5 pathologists' performance using a measure derived from the ROC curve. All participating pathologists had at least five years of experience in breast cancer slide diagnosis, with some practising for over 30 years, and were blinded to the patients’ clinical background. Immunofluorescence, the most accurate method for HEV involvement detection, was used to generate a reference standard with little interpretation variability.

Next, we adopted the trained networks to analyse the three independent SLN-positive cohorts: 559 WSIs from the Sun Yat-Sen Memorial Hospital (SYS) cohort, 207 WSIs from the Affiliated Hospital of Guizhou Medical University (GZ) cohort and 213 WSIs from the First Affiliated Hospital of Zhengzhou University (ZZ) cohort. The framework performed robustly across cohorts without transfer learning. Kaplan–Meier analysis was used to assess survival based on HEV involvement, with log-rank tests for DFS and OS. Cox regression models were used to evaluate the magnitude of differences in DFS rate, adjusting for covariates. We made an assessment for the interaction between the number of positive SLNs and HEV involvement by adding an interaction term into a Cox model, with the likelihood ratio test for significance.

### Animal models

For intravital multiphoton microscopy, 5 × 10^5^ GFP-expressing EO771 cells were injected into the fourth mammary fat pad of *Chst4-tdTomato* mice. After 20 days^7^, skin was cleared of hair, and a custom-built lymph node window (CLNW)^144^ was implanted. *Chst4-tdTomato* mice were anaesthetised with 1.5–2% (v/v) isoflurane in O₂ and maintained on a 37 °C heating pad. An Olympus laser-scanning microscope (FVMPE-RS) was used to capture the cancer cell migration through the CLNW with 0–200 μm depths using a 25×, 0.95 NA water immersion objective lens.

For photoconversion experiments, 5 × 10^5^ Dendra2H2B-EO771 or Dendra2H2B-B16F10 cells and 2 × 10^6^ Dendra2H2B-Py230 cells were implanted into C57BL/6 mice (EO771 and Py230 in the fourth mammary fat pad; B16F10 intradermally in the flank). To block efferent lymphatic drainage, the efferent vessel was surgically ligated^6^. The ligation of lymphatics was only performed in the experiments to enrich tumour cells that preferentially spread via LNs, but was not conducted in other animal experiments. Each tumour was measured every 3 days after tumours were palpable (about 7 days after injection). Tumour volumes were estimated using the formula: volume = large diameter × short diameter^2^ × 0.5. CLNW were installed 20 days after the implantation of Dendra2H2B-EO771 cells, 12 days after the implantation of Dendra2H2B-B16F10 cells or 35 days after the implantation of Dendra2H2B-Py230 cells, when tumour cells have colonised the LNs^7^. Briefly, mice were anaesthetised with 100 mg/kg ketamine (Sigma Aldrich, #1356009) and 10 mg/kg xylazine (Sigma Aldrich, #X1126). The area of skin over the one side of the flank, thigh and abdomen was depilated. The inguinal LN was visible and identified through the skin between the anatomical surface markers (thigh, the lowest nipples and the lateral axillo-inguinal skin fold). A custom-built 405 nm diode at 11 mW was used to photoconvert the LN metastases for 3 min everyday afterwards until sacrificing the mice.

To enrich tumour cells that preferentially spread via LNs, lungs were harvested 7 days after photoconversion of Dendra2H2B-EO771 cells, Dendra2H2B-B16F10 cells and Dendra2H2B-Py230 cells. Then, lung tissues were minced using surgical scissors and digested in DMEM medium containing 5% FBS (Gibco, #10099-141), 2 mg/ml collagenase I (Worthington, #LS004196), 2 mg/ml collagenase Ⅳ (Worthington, #LS004188) and 100 μg/ml DNase I (Sigma Aldrich, #DN25) for 1 h at 37 °C with gentle rocking. FBS (final concentration 30%) was used to halt the digestion. After filtering through 100 μm and 70 μm strainers (BIOLOGIX, #15-1100, #15-1070), cells were pelleted at 250 × *g* for 5 min, and resuspended in PBS (Thermo Fisher Scientific, #10010072) with 1% FBS. Cells were isolated by FACS using 488 nm and 561 nm lasers. Native green^+^red^−^ tumour cells were collected and designated as generation 1 “Blood Vessel Metastatic (BVM1)” cells, while photoconverted green^+^red^+^ tumour cells were designated as generation 1 “HEV Metastatic (HEVM1)” cells. After about 7 days of ex vivo culture, all red-emitting tumour cells returned to green in our experiments. Afterwards, BVM1 and HEVM1 cells suspended in 100 μl PBS were injected into C57BL/6 mice again. After three rounds of this selection, the derivative cells were harvested and designated as “BVM3” and “HEVM3”, respectively.

To evaluate the difference in metastasis between 1st generation (BVM1 and HEVM1) and 3rd generation (BVM3 and HEVM3), luciferase-expressing 1st generation (BVM1 and HEVM1) and 3rd generation (BVM3 and HEVM3) cells were generated by lentiviral transduction in the presence of polybrene as aforementioned. Then, 5 × 10^5^ luciferase-expressing indicated 1st generation (BVM1 and HEVM1) and 3rd generation (BVM3 and HEVM3) cells were injected into the mammary fat pad or implanted intradermally in C57BL/6 mice. To evaluate whether the difference between BVM3 and HEVM3 in metastasis is attributed to immunogenicity, 5 × 10^5^ luciferase-expressing EO771-BVM3 and EO771-HEVM3 cells were injected into the fourth mammary fat pad of NOD/SCID mice.

To specifically trace tumour cells disseminating through HEVs, 5 × 10^5^ luciferase-GFP-EO771 cells were injected into the fourth mammary fat pad of *Chst4-CreERT2*;*R26-LSL-sLP-mCherry* mice to induce LN metastases. When tumours were palpable (about 7 days after tumour inoculation), we injected tamoxifen (Sigma Aldrich, #T5648) intraperitoneally (200 μg/g body weight) for five consecutive days to excise the floxed stop cassette upstream of the sLP-mCherry gene and induce specific expression and secretion of sLP-mCherry in HEVs. Lungs were harvested 25 days after implantation of luciferase-GFP-EO771 cells. Then, GFP⁺mCherry⁺ and GFP⁺mCherry⁻ tumour cells were isolated and sorted by FACS as described above. GFP⁺mCherry⁺ tumour cells were identified as HEV-emigrant, while GFP⁺mCherry⁻ cells represented tumour cells disseminating through lymphatics or blood vessels. After about 5 days of ex vivo culture, GFP⁺mCherry⁺ tumour cells lost the sLP-mCherry signal. Afterwards, 5 × 10^5^ GFP⁺mCherry⁺ and GFP⁺mCherry⁻ tumour cells were injected into the mammary fat pad of syngeneic recipient mice to evaluate the metastatic potential. Metastatic burden was assessed by bioluminescence imaging at humane endpoint.

To investigate the functions of ALOX12 in vivo, we injected intravenously ML355 or CDC (3 mg/kg body weight) with or without 12-HETE every day for 20 consecutive days when the tumours were palpable. To specifically investigate the functions of ALOX12 in HEVs, 5 × 10^5^ luciferase-expressing EO771 cells, EO771-HEVM1, EO771-HEVM3 cells and 2 × 10^6^ luciferase-expressing Py230 cells were injected into the fourth mammary fat pad of wild-type C57BL/6 mice or *Alox12*^*fl/fl*^;*Chst4-CreERT2* mice. 5 × 10^5^ luciferase-expressing B16F10 cells were implanted intradermally in C57BL/6 mice or *Alox12*^*fl/fl*^;*Chst4-CreERT2* mice. When tumours were palpable (about 7 days after tumour inoculation), we injected tamoxifen (Sigma Aldrich, #T5648) intraperitoneally (200 μg/g body weight) for five consecutive days to knock out *Alox12* in HEVs.

To evaluate the effect of 12-HETE (Cayman, #34570) on metastasis, 5 × 10^5^ EO771 cells pretreated with vehicle (2% DMSO in saline) or 100 nM 12-HETE were intravenously injected into *Alox12*^*fl/fl*^;*Chst4-CreERT2* mice via the tail vein. In some experiments, 5 × 10^5^ EO771 cells were treated with vehicle (2% DMSO in saline), 100 nM 12-HETE or 12-HETE with 3% 1,6-hexanediol (Sigma Aldrich, #240117) for 48 h. Then the cells were injected into the fourth mammary fat pad of wild-type C57BL/6 mice or *Alox12*^*fl/fl*^;*Chst4-CreERT2* mice. Vehicle, 100 nM 12-HETE or 12-HETE with 3% 1,6-hexanediol was injected into the grafts every 2 days afterwards.

In some experiments, we employed SEMA3C-KO, NRG1-KO, TFF2-KO, APOC4-KO, RBP4-KO, ZBP1/MDA5-KO, KO + WT ADAR1 p150, KO + ADAR1 p150 (V677A/L681A), KO + WT ADAR1 p110, KO + ADAR1 p110 (V429A/L433A) and KO + ADAR1 p150 (E861A) EO771 tumour cells constructed as aforementioned. 5 × 10^5^ luciferase-expressing EO771 cells with indicated genetic modifications were injected into the fourth mammary fat pad of C57BL/6 mice or *Alox12*^*fl/fl*^;*Chst4-CreERT2* mice.

To investigate the functions of PlexinA1, we injected 5 × 10^5^ luciferase-expressing EO771 into the fourth mammary fat pad of wild-type C57BL/6 mice. We used a previously described specific PlexinA1 antagonist peptide MTP-PlexA1^145^, which was synthesised by Labgene (China) and solubilised in LDS (lithium dodecyl sulphate). Intraperitoneal administration of 1 μg/kg LDS (72 µM, Sigma Aldrich, #L4632) or 1 μg/kg MTP-PlexA1 was performed every day for 20 consecutive days^146^ when the tumours were palpable.

For in vivo bioluminescence imaging (BLI), mice were anaesthetised with ketamine (100 mg/kg, Sigma Aldrich, #1356009) and xylazine (10 mg/kg, Sigma Aldrich, #X1126) at 30 days after implantation of luciferase-EO771 cells, 20 days for B16F10, or 45 days for Py230. D-luciferin (150 mg/kg, Thermo Fisher Scientific, #L2916) was injected intraperitoneally 10 min before sacrifice. Imaging was conducted on an IVIS Spectrum Xenogen system (Caliper Life Sciences). In some experiments, lungs were then harvested for ex vivo BLI.

### Immunohistochemistry and immunofluorescence

For immunohistochemistry, mouse lung tissues were fixed and cut by vibratome (Leica). After antigen retrieval in EDTA buffer (pH 8.0) for 3 min, sections were incubated overnight at 4 °C with rabbit anti-mouse CD8α (1:2000, Abcam, #ab217344), mouse anti-mouse NK1.1 (1:100, Thermo Fisher Scientific, #MA1-70100) and rabbit anti-human/mouse cleaved caspase-3 (1:100, Cell Signaling Technology, #9661). Following PBS washes, staining was performed with a mouse/rabbit IHC Secondary Antibody Kit (GTVision, #GK500705). Images were obtained with an Olympus BX63 microscope and analysed in QuPath in a blinded manner. Positive cell detection was applied to quantify DAB-positive cells, with haematoxylin-stained nuclei used as total cell counts. For a given stain, all slides used identical settings and thresholds. The counting was performed in five randomly selected fields of the lung metastases^147^.

For immunofluorescence analysis, primary tumours and LNs were embedded in OCT (Sakura, #4583) and sectioned at 10 μm thickness using Thermo NX50. The slices were incubated with mouse anti-human/mouse pan CK (1:100, Sigma Aldrich, #c2562), rat anti-human/mouse PNAd (1:50, Biolegend, #120802), rabbit anti-mouse CD11c (1:100, Cell Signaling Technology, #97585), rat anti-mouse CD8α (1:100, Abcam, #ab308264), rabbit anti-human/mouse CD31 (1:2000, Abcam, #ab182981), mouse anti-human/mouse ALOX12 (1:150, Novus Biologicals, #NBP2-46512), goat anti-human/mouse CK18 (1:100, Abcam, #ab219271), rabbit anti-human/mouse SEMA3C (1:100, Abcam, #ab214309), rabbit anti-human/mouse CX40 (1:50, Thermo Fisher Scientific, #36-4900) and goat anti-mouse EMCN (1:200, Thermo Fisher Scientific, #PA5-47648) at 4 °C overnight. Alexa Fluor secondary antibodies (1:300, Thermo Fisher Scientific, #A21202, #A21434, #A31573, #A48261, #A48259 and #A21432) were applied according to the experiment design. Isotype-matched control antibodies were used in parallel as negative controls, and the MFI from isotype controls was used to establish background levels and set thresholds for quantitative immunofluorescence analysis. Immunofluorescence was imaged using a laser-scanning confocal microscopy (LSM800, Zeiss).

For whole LN staining, LNs were fixed overnight in 4% paraformaldehyde and embedded in 4% agarose (Sigma Aldrich, #A9539) at 4 °C for polymerisation. Agarose-embedded nodes were cut into 200 μm slices. Slices were incubated with rabbit anti-human/mouse CD31 (1:2000, Abcam, #ab182981) overnight at 4 °C, followed by incubation with Alexa Fluor secondary antibodies (1:300, Thermo Fisher Scientific, #A31573). The whole LN immunofluorescence images were captured using an Andor Dragonfly CR-DFLY confocal microscope (20× objective), and images were reconstructed with IMARIS software (v8.2).

To capture photoconverted cells in the lung tissues, lungs were harvested 7 days after photoconversion of Dendra2H2B-EO771 cells, Dendra2H2B-B16F10 cells and Dendra2H2B-Py230 cells and then embedded in OCT and three sections per mouse were cut into 70 μm thick using Thermo NX50. Native and photoconverted Dendra2H2B-expressing tumour cells were detected at 488 nm and 543 nm wavelengths, respectively, in sections using a laser-scanning confocal microscopy (LSM800, Zeiss). To capture GFP^+^mCherry^+^ tumour cells in the LN tissues, LNs were harvested 20 days after implantation of GFP-EO771 cells and then embedded in OCT and three sections per mouse were cut into 70 μm thick using Thermo NX50. PNAd staining was performed as described above for the identification and delineation of HEVs. GFP^+^mCherry^+^ tumour cells were detected at 488 nm and 543 nm wavelengths, respectively, in sections using a laser-scanning confocal microscopy (LSM800, Zeiss).

To investigate the protein levels and localisation of MDA5/ZBP1, EO771 cells were grown on a 24-well high-content screening microplate with glass bottom (Cellvis, #P24-0-N) and treated with vehicle (2% DMSO in saline) or 100 nM 12-HETE (Cayman, #34570) for 48 h. After being washed, the cells were fixed, blocked in 5% normal goat serum for 1 h and stained with the primary antibody: rabbit anti-human/mouse MDA5 (1:100, Abcam, #ab79055), rabbit anti-human/mouse ZBP1 (1:100, Thermo Fisher Scientific, #PA5-20455) and mouse anti-J2 (1:100, Sigma Aldrich, #MABE1134) overnight at 4 °C. The Alexa Fluor secondary antibodies (1:300, Thermo Fisher Scientific, #A31570, #A-11034) were incubated for 1 h at room temperature.

For quantitative analysis of confocal microscopy images, we used CellProfiler software (www.cellprofiler.org). For all the quantification except for whole sections, the counting was performed in five randomly selected fields of one slice and three slices from one mouse. To quantify the proportion of ALOX12^+^ HECs in total HECs, we defined HEC ‘primary objects’ on the PNAd channel of images and ALOX12 ‘primary objects’ on the ALOX12 channel of images based upon user-defined parameters. Then, the ALOX12^+^ HECs were detected and counted using “RelateObjects” and “FilterObjects” modules in the CellProfiler, respectively. We measured the proportion of SEMA3C^+^ tumour cells, ALOX12^+^ artery and ALOX12^+^ capillary with the same pipeline. To quantify the HEV density and metastatic burden in the whole section of LNs, we defined HECs and tumour cells as described above. Then, the total area of HECs, tumour cells and whole LN sections was measured using “MeasureImageAreaOccupied” module in the CellProfiler, respectively. HEV density was defined as the total area of HECs divided by the area of the whole LN sections. Metastatic burden was defined as the total area of tumour cells divided by the area of whole LN sections. To quantify the number of intravasating tumour cells in the whole section of LNs, tumour cells and HECs were defined as described above. The tumour cell objects were defined as “intravasating” if any overlapping pixels were identified between tumour cell objects and HECs objects by “RelateObjects” module in the CellProfiler. The number of intravasating tumour cells and total tumour cells was quantified to calculate the relative number of intravasating tumour cells. To calculate the native and photoconverted Dendra2H2B-expressing tumour cells in lungs, CellProfiler was used to count all objects in the green channel and identify these objects as native tumour cells. Also, CellProfiler was used to count all objects in the red channel and identify these objects as photoconverted tumour cells. The number of HEVs and non-HEV LN blood vessels intravasated by tumour cells was quantified manually. Briefly, HEVs were identified as CD31^+^tdTomato^+^ and non-HEV blood vessels were identified as CD31^+^tdTomato. The HEVs and non-HEV blood vessels contacting tumour cells were identified as tumour-associated ones. Then, the proportion of tumour-associated vessels was calculated as the number of tumour-associated vessels divided by the total number of vessels. The size of the DC cluster with CD8^+^ T cells was identified as the average number of CD8^+^ T cells contacting an individual DC. Briefly, CD11c and CD8α were used to identify DCs and CD8^+^ T cells. Then the number of CD8^+^ T cells contacting an individual DC was counted manually. To quantify the proportion of J2^+^ dsRNA colocalised with MDA5 or ZBP1 in total dsRNA, we defined dsRNA ‘primary objects’ on the J2 channel of images and MDA5 or ZBP1 ‘primary objects’ on the MDA5 or ZBP1 channel of images based upon user-defined parameters. Then the dsRNA colocalised with MDA5 or ZBP1 was detected and counted using “RelateObjects” and “MeasureImageAreaOccupied” modules in the CellProfiler, respectively. To quantify the MDA5 or ZBP1 protein levels in the cells, we identified MDA5 or ZBP1 ‘primary objects’ as described above. Then the intensities of MDA5 or ZBP1 were measured using the “MeasureObjectIntensity” module in the CellProfiler.

### Cell migration and invasion assays

Assays were conducted using 24-well Boyden chambers (Corning, #354480) with 8 μm pore size filters. Filters were pre-coated with 10 μg/ml fibronectin (Sigma Aldrich, #F0635) for migration assays and with both 10 μg/ml fibronectin and 10 μg/ml Matrigel (Corning, #356234) for invasion assays^148^. EO771-BVM3 and EO771-HEVM3 cells (1 × 10^5^ per insert) were suspended in 0.5 ml serum-free DMEM (Gibco, #11905065) and added to the upper chambers; lower wells contained DMEM supplemented with 10% FBS (Gibco, #10099-141) as chemoattractant. Cells on the underside of the membrane were fixed with methanol for 5 min at room temperature and stained with crystal violet for 5 min after 12 h incubation. Imaging was performed using a Leica DMI4000B microscope, and quantification was based on the mean cell counts from five randomly selected fields per insert.

### MTT assay

EO771-BVM3 and EO771-HEVM3 cells were plated in 96-well plates at 2000 cells per well. Cell proliferation was assessed using an MTT assay after 24 h. Briefly, 20 µl of MTT solution (5 mg/ml; Thermo Fisher Scientific, #M6494) was added to each well and incubated at 37 °C for 1 h. After removing the medium, 100 µl of DMSO was added to dissolve the formazan crystals. Absorbance was read at 550 nm, and the relative proliferation rates of the two cell lines were calculated.

### Whole-exome sequencing

DNA from EO771, Py230 and B16F10 cell lines was isolated using the iPure tissue DNA kit (IGE Biotechnology, #K316). Libraries were constructed using the Agilent SureSelectXT Mouse All Exon kit (Agilent Technologies, #5190-4643). Sequencing (2 × 150 bp) was performed on an Illumina Novaseq 6000. Raw sequencing reads were filtered using FastQC (https://www.bioinformatics.babraham.ac.uk) under default settings. Clean paired-end reads were aligned to the mouse reference genome (GRCm39.91) using the Burrows-Wheeler-Alignment Tool^149^. SNPs and InDels (total count ≥ 10) were identified and filtered using the Genome Analysis Toolkit (GATK, Broad Institute). Somatic SNPs and InDels in tumour cells were detected by muTect^150^ and Strelka^151^, respectively. Variant annotation was performed with ANNOVAR^152^.

### In vitro T cell cytotoxicity assays

Cytotoxicity assays were performed as we previously described^153,154^. Briefly, to isolate mouse T cells from C57BL/6 mice, splenocytes were mechanically dissociated and passed through 40 μm strainers (BIOLOGIX, #15-1040). CD8^+^ T cells were then isolated using a CD8^+^ T Cell Isolation Kit (Miltenyi Biotec, #130-117-044). These cells were maintained in RPMI-1640 medium (GIBCO, #11875093) supplemented with 10% FBS (Gibco, #10099-141) and 25 U/ml IL-2 (PeproTech, #212-12), with media refreshed every other day. To generate mouse DCs, bone marrow was flushed from femurs and tibias into RPMI-1640 containing 10% FBS and 1% penicillin-streptomycin (Thermo Fisher Scientific, #15140122). After filtering through 40 μm strainers, cells were cultured with 20 ng/ml GM-CSF (PeproTech, #315-03) and 10 ng/ml IL-4 (PeproTech, #214-14), with medium changes every 2 days. Five freeze (liquid nitrogen) and thaw (37 °C water bath) cycles were subjected to 2 × 10^7^ tumour cells to obtain tumour lysates. After centrifugation (2000 × *g* for 10 min at 4 °C), the supernatant was collected and stored at -80 °C until use. On day 7, DCs were activated for 48 h using 10 ng/ml LPS (Sigma Aldrich, #L2880) and 500 U/ml IFN-γ (PeproTech, #315-05), and then pulsed with tumour lysates (200 mg protein/1 × 10^6^ cells/ml) for 24 h, followed by co-culture with CD8⁺ T cells (at a 1:5 DC:T ratio) in IL-2-supplemented RPMI-1640 for 5 days to generate tumour-reactive cytotoxic T lymphocytes. CD8^+^ T cells were purified using CD8 Microbeads. Tumour cells were pre-stained with 1 μM CellTracker Deep Red Dye (Thermo Fisher Scientific, #C34565) for 15 min at 37 °C. Cytotoxicity assays for HEVM3 derivatives and GFP^+^mCherry^+^ tumour cells were performed after their red fluorescence had completely decayed. Effector CD8^+^ T cells (2 × 10^4^) were co-incubated with target cells at a 10:1 ratio for 12 h. Cells were then stained with propidium iodide (1:20, eBioscience, #00-6990-50) and immediately analysed by flow cytometry (CytoFLEX, Beckman Coulter). Cells positive for both Deep Red and propidium iodide were identified as killed tumour cells.

### Isolation of endothelial cells from mice

To isolate BECs in mammary fat pads and primary tumours and HECs and non-HEV BECs in LNs, the mammary fat pads, primary tumours and inguinal LNs were carefully dissected from wild-type C57BL/6 mice inoculated with or without tumour cells. After light mincing, LN tissues were enzymatically dissociated in RPMI-1640 medium (GIBCO, #11875093) containing 0.2 mg/ml collagenase P (Sigma Aldrich, #11213857001), 0.8 mg/ml dispase Ⅱ (Sigma Aldrich, #D4693) and 0.01 mg/ml DNase I (Sigma Aldrich, #DN25) for 1 h at 37 °C under constant shaking. The fat pad tissues were digested with RPMI-1640 medium containing 2 mg/ml collagenase I (Worthington, #LS004196) for 30 min at 37 °C. The tumour tissues were digested with DMEM medium (Gibco, #11905065) containing 2 mg/ml collagenase I (Worthington, #LS004196), 2 mg/ml collagenase III (Worthington, #LS004182) and 2 mg/mL hyaluronidase (Sigma Aldrich, #H3506) for 2 h at 37 °C. FBS (final concentration 30%) was used to halt the digestion. After filtering through 100 and 40 μm cell strainers (BIOLOGIX, #15-1100 and #15-1040), cells were stained with Pacific Blue anti-mouse CD45 (1:25, Thermo Fisher Scientific, #MCD4528), APC anti-mouse/human CD31 (1:25, Thermo Fisher Scientific, #17-0311-82) and AF488 anti-mouse/human PNAd (1:25, Thermo Fisher Scientific, #53-6036-82) for 30 min at 4 °C for FACS to purify the BECs and HECs using a flow cytometer (BD Biosciences)^103,155^. Healthy singlet populations were identified by FSC/SSC gating, while doublets were removed using FSC height versus area criteria. Endothelial cells were identified as CD45^−^CD31^high^, and the CD31^high^ subset was subsequently divided into PNAd^+^ HECs and PNAd^−^ non-HEV BECs.

### Isolation of primary cells from patients

Tumour-BECs from primary tumours, HECs from LNs of LN-negative or LN-positive breast cancer patients were also isolated for qRT-PCR. After light mincing, LN tissues were digested with RPMI-1640 medium (GIBCO, #11875093) containing 0.2 mg/ml collagenase P (Sigma Aldrich, #11213857001), 0.8 mg/ml dispase Ⅱ (Sigma Aldrich, #D4693) and 0.01 mg/ml DNase I (Sigma Aldrich, #DN25) for 1 h at 37 °C under constant shaking. Tumour tissues were digested with DMEM medium (Gibco, #11905065) supplemented with 2 mg/ml collagenase type I (Worthington, #LS004196), 2 mg/ml collagenase type III (Worthington, #LS004182) and 2 mg/ml hyaluronidase (Sigma Aldrich, #H3506) for 3 h at 37 °C. After filtering through 100 μm strainers (BIOLOGIX, #15-1100), cells were subsequently stained with Pacific Blue anti-human CD45 (1:25, Thermo Fisher Scientific, #MHCD4528), APC anti-human CD31 (1:25, Thermo Fisher Scientific, #17-0319-42) and AF488 anti-mouse/human PNAd (1:25, Thermo Fisher Scientific, #53-6036-82) for 30 min at 4 °C for FACS to purify the BECs and HECs as described above.

### Flow cytometry

To detect photoconverted cells in lungs, dissociated lung suspensions were prepared as described above and then analysed by the CytoFLEX flow cytometer (Beckman Coulter). Cells were stained with Fixable Viability Dye eFluor 780 (Thermo Fisher Scientific, #65-0865-14) to exclude dead cells. Native cells were identified by green fluorescence alone, whereas photoconverted cells displayed both green and red signals upon excitation with 488 nm and 543 nm lasers. Similarly, GFP⁺mCherry⁺ and GFP⁺mCherry⁻ tumour cells were sorted by FACS using the same gating strategy.

To delineate the immune landscape in mouse mediastinal LNs, the mediastinal LNs were carefully dissected from wild-type C57BL/6 mice bearing the indicated tumour cells. LN suspensions were prepared as described above and then were sequentially filtered through 40 μm cell strainers (BIOLOGIX #15-1040). For analysis of CD103^+^ DCs and MDSCs in mediastinal LNs, cells were stained with BV510 anti-mouse CD45 (1:25, BD Biosciences, #563891), FITC anti-mouse CD90.2 (1:25, BD Biosciences, #553003), FITC anti-mouse CD19 (1:25, BD Biosciences, #557398), APC-Cy7 anti-mouse Ly6C (1:25, BD Biosciences, #560596), PE-Cy7 anti-mouse CD24 (1:25, Thermo Fisher Scientific, #25-0242-82), PerCP-Cy5.5 anti-mouse CD11b (1:25, Biolegend, #101228), PE anti-mouse CD11c (1:25, Biolegend, #117308), APC anti-mouse MHCⅡ (1:25, Biolegend, #116418), BV421 anti-mouse CD103 (1:25, BD Biosciences, #562771), BV605 anti-mouse CD40 (1:25, BD Biosciences, #745218) and BV711 anti-mouse CD80 (1:25, BD Biosciences #740698) for 30 min at 4 °C. Migratory CD103^+^ DCs were gated as CD45^+^, CD90.2^−^, CD19^−^, Ly6C^−^, CD24^+^, CD11c^+^, MHCII^hi^ and CD103^+^. To analyse CD8^+^ and CD4^+^ T cells in mediastinal LNs, the single-cell suspensions were incubated with BV510 anti-mouse CD45 (1:25, BD Biosciences, #563891), PE anti-mouse CD3 (1:25, Biolegend, #100205) and either APC anti-mouse CD8a (1:25, Biolegend, #100711) or APC anti-mouse CD4 (1:25, Biolegend, #100411). Cells were then fixed and permeabilised using Fixation/Permeabilisation buffer (eBiosciences, #88-8824) for 45 min, and subjected to intracellular staining with the FITC anti-human/mouse Granzyme B (1:25, Biolegend, #515403) or Alexa Fluor 488 anti- human/mouse FOXP3 (1:25, Biolegend, #320011).

In FACS experiments, the dissociated mouse samples were incubated with Pacific Blue anti-mouse CD45 (1:25, Thermo Fisher Scientific, #MCD4528), APC anti-mouse/human CD31 (1:25, Thermo Fisher Scientific, #17-0311-82) and AF488 anti-mouse/human PNAd (1:25, Thermo Fisher Scientific, #53-6036-82). The dissociated human samples were incubated with Pacific Blue anti-human CD45 (1:25, Thermo Fisher Scientific, #MHCD4528), APC anti-human CD31 (1:25, Thermo Fisher Scientific, #17-0319-42) and AF488 anti-mouse/human PNAd (1:25, Thermo Fisher Scientific, #53-6036-82).

In some experiments, the mouse samples were incubated with Pacific Blue anti-mouse CD45 (1:25, Thermo Fisher Scientific, #MCD4528), APC anti-mouse/human CD31 (1:25, Thermo Fisher Scientific, #17-0311-82), AF488 anti-mouse/human PNAd (1:25, Thermo Fisher Scientific, #53-6036-82) and PE anti-mouse ALOX12 (1:25, LSBio, #LS-C212708) for 30 min at 4 °C.

### RNA-seq and bioinformatics data analysis

Total RNA was extracted with Trizol (Invitrogen, #15596026), and RNA quality was assessed using an Agilent 2100 Bioanalyzer. The cDNA library was then constructed using NEB Next Ultra Ⅱ RNA Library Prep kit (NEB, #E7775) and then sequenced on an Illumina Novaseq 6000 instrument. Sequencing reads were mapped to the NCBI reference genome (GRCm38.91) using STAR (v.2.6.0c)^156^. All fragment quantifications were computed using featureCounts (v.1.6.2)^157^. In the count matrix, rows with an average count number of less than 10 were removed, then the R package DESeq2 (v1.34.0) was run with default parameters. The differentially expressed mRNAs were selected with absolute fold change >2 or fold change <0.5 and FDR-adjusted values of *P* < 0.05 by DESeq2^158^. Heatmap plots were generated using z-score-transformed log_2_(1 + normalised count). GSEA was performed with ClusterProfiler using FDR *P*-value adjustment with default parameters^159^. The raw data from RNA-seq is deposited in the Gene Expression Omnibus (GEO) repository (GSE217454).

### scRNA-seq

scRNA-seq was performed as we previously described^153^. Briefly, single-cell suspension of primary tumours and paired LNs were prepared as described above from six C57BL/6 mice. After treating with 2 ml of 1× RBC lysis buffer (Biolegend, #420301) on ice for 10 min, cells were washed, resuspended and counted with TC20 automated cell counter. In parallel, BECs from metastatic LNs were sorted as described above and processed for scRNA-seq. The single-cell suspensions were adjusted to 1 × 10^5^ cells/ml in PBS and used to generate barcoded libraries with the Chromium Single-Cell 3′ Library, Gel Bead & Multiplex Kit and Chip Kit (10× Genomics). Briefly, cells were loaded into each reaction, where RNA transcripts were barcoded and reverse-transcribed. Complementary DNA (cDNA) was generated, amplified, fragmented, end-repaired, and ligated with adaptors to generate sequencing libraries, which were sequenced on an Illumina Novaseq 6000 and aligned to the mouse genome (GRCm38.91) using CellRanger software (10× Genomics).

The raw data was processed and analysed by the Seurat package in R (v. 4.1.1). Genes detected in <50 cells or with a row mean <0.01, cells with <201 or > 6000 detected genes or >5% mitochondrial unique molecular identifiers (UMIs) were excluded. Undetected genes in any cell were also removed. Doublets were identified and filtered using DoubletFinder. The data were normalised with the Seurat package (v. 4.1.1), and cell cycle effects were computed with the CellCycleScoring function and regressed out using ScaleData. After scaling, dimensionality reduction was carried out using principal component analysis (PCA) and Uniform Manifold Approximation and Projection (UMAP). Harmony integration was applied to reduce batch effects. Clustering was conducted with FindClusters, and marker genes for each cluster were identified with FindAllMarkers. Cell clusters were subsequently manually annotated based on a combination of canonical markers from the literature and gene ontology using Enrichr. To identify the malignant cells, the inferCNV algorithm was adopted to analyse the copy-number variants (CNVs) of a single cell. The analysis of cell-cell communication was performed using CellChat^68^. The raw data from scRNA-seq is deposited in the GEO repository under accession code GSE217454.

Processed scRNA-seq data of human metastatic LNs (GEO: GSE103322) was downloaded and analysed by the Seurat package in R (v. 4.1.1) as described above.

### qRT-PCR

Total RNA was extracted using Trizol (Invitrogen, #15596026) according to the manual instructions. In some experiments, platelet-rich plasma (PRP) was prepared by centrifugation at 200  × *g* for 10 min and treated with prostaglandin E1 (1 mM; Sigma Aldrich, #P5515). Platelets were collected by centrifugation at 800 × *g* for 10 min at room temperature, and platelet RNA was extracted using RNA STAT-60 (Thermo Fisher Scientific, #NC9884083). RNA quality was assessed with a NanoDrop ND-1000. qRT-PCR was conducted with the SYBR Premix Ex Taq kit (TaKaRa, #RR039A) on a LightCycler 480 (Roche), and primers are provided in Supplementary Dataset 4.

### Metabolite quantification by mass spectrometry

Frozen LN samples were mixed with 80% methanol (10 μl per mg). After being vortexed for 1 min at maximum speed, the lysates were incubated at 4 °C for 20 min and centrifuged at 12,000 × *g* for 10 min at 4 °C to collect the supernatant. After being dried under nitrogen gas, the extracts were reconstituted in 200 μl of mobile phase (B) acetonitrile/isopropanol (10/90, V/V) for LC-MS/MS analysis. Meanwhile, graded standards (Cayman) were dissolved in acetonitrile to manufacture a standard curve. Lipid extracts were analysed in an Ultra Performance Liquid Chromatography (Shim-pack UFLC SHIMADZU CBM30A system, SHIMADZU) coupled to Tandem mass spectrometry (QTRAP, SCIEX). Samples were injected onto a 2.6-μm particle 100 × 2.1 mm ID Thermo C30 column (5 μl per sample). The mobile phase included (A) acetonitrile/water (60/40, V/V) supplemented with 0.04% ethanoic acid or 5 mmol/L ammonium formate, and (B) acetonitrile/isopropanol (10/90, V/V) supplemented with 0.04% ethanoic acid or 5 mmol/L ammonium formate. The gradient elution programme was as follows: 0–2.0 min 20% B, 2.0–4.0 min 30% B, 4.0–9.0 min 65% B, 9.0–14.0 min 85% B, 14.0–15.5 min 90% B, 15.5–17.5 min 95% B and 17.5–20 min 20% B. The column temperature was 45 °C, and the flow rate was 350 μl/min. Heated electrospray ionisation (H-ESI) parameters were set as follows: 5.5 kV positive mode of capillary voltage, curtain gas 35 psi, temperature 550 °C and medium collision-activated dissociation. Based on our targeted standard metware database, targeted components’ retention time, precursor ion, product ion scan and second mass analyser scan data were used for qualitative analysis with multiple reaction monitoring (MRM) mode and peak areas extracted in Analyst 1.6.3 and normalised to internal standards^160^.

### Correlation analysis

In order to select the genes associated with *ALOX12* expression in LN metastases, we downloaded the GSE32489 dataset from the GEO datasets. The cohort includes 90 metastatic LN samples from breast cancer patients. Spearman’s rank correlation coefficients were computed in R (v. 4.1.1) with the cor.test function.

### Western blotting

Protein was extracted by RIPA buffer (Beyotime, #p0013b) supplemented with proteinase inhibitor cocktail (1:100, Thermo Fisher Scientific, #78446), followed by centrifugation at 16,000 × *g* for 20 min at 4 °C. Protein concentration was measured with the Pierce BCA Protein Assay Kit (Thermo Fisher Scientific, #23225). Lysates were mixed with 4× Laemmli SDS sample buffer (Thermo Fisher Scientific, #J63615) and denatured at 95 °C for 5 min. Equal amounts of protein (15 µg) were separated on 10% SDS-PAGE gels and transferred to nitrocellulose membranes (Merck, #WHA10402506). After blocking with 5% skim milk (Asegene, #AS430756) in TBS-T at room temperature for 90 min, the membranes were incubated with following primary antibodies: rabbit anti-human/mouse SEMA3C (1:1000, Thermo Fisher Scientific, #PA5-24997), rabbit anti-human/mouse NRG1 (1:1000, Abcam, #ab217805), rabbit anti-human/mouse TFF2 (1:1000, Thermo Fisher Scientific, #PA5-75670), rabbit anti-human/mouse APOC4 (1:1000, Thermo Fisher Scientific, #PA5-75699), rabbit anti-human/mouse RBP4 (1:1000, Abcam, #ab109193), rabbit anti-human/mouse MDA5 (1:1000, Abcam, #79055), rabbit anti-human/mouse ZBP1 (1:2000, Thermo Fisher Scientific, #PA5-20455), anti-GADPH (1:10,000, Proteintech, #HRP-60004), mouse anti-human/mouse ADAR1 (1:1000, Santa Cruz Biotechnology, #sc-73408), rabbit anti-human/mouse SOCS1 (1:500, Thermo Fisher Scientific, #38-5200), rabbit anti-human/mouse PTPN2 (1:500, Thermo Fisher Scientific, #PA5-78138) and anti-β-tubulin (1:5000, Proteintech, #10068-1-AP) at 4 °C overnight. The membranes were washed three times with 0.05% Tween 20 in TBS (TBS-T) and incubated for 1 h at room temperature with HRP-conjugated secondary antibodies: anti-mouse antibody (1:3000, Cell Signaling Technology, #7076) and anti-rabbit antibody (1:3000, Cell Signaling Technology, #7074). Signals were detected using enhanced chemiluminescence (ECL, Thermo Fisher Scientific, #32106) on a Syngene G:BOX imaging system.

### ELISA

ELISA assays were performed according to the manufacturer’s instructions. The supernatants of cell culture and mouse serum were harvested and stored at −80 °C. The levels of IFNα, IFNβ and SEMA3C were measured by the ELISA kit (PBL, #42115-1), (PBL, #42410-1) and (LSBio, #LS-F19645), respectively.

### In vivo proximity labelling

PCDH lentiviral vectors carrying a secreted p65-Sema3C-TurboID fusion gene or a signal peptide*-*TurboID control were produced by Shanghai GeneBiogist. Lentivirus were transduced into EO771 cells as described above to obtain secreted Sema3C-TurboID- and TurboID-overexpressing tumour cells. A total of 5 × 10^5^ secreted Sema3C-TurboID- and TurboID-overexpressing EO771 cells were injected into the fourth mammary fat pad of C57BL/6 mice. Twenty days after tumour implantation, when tumour cells had colonised the lymph nodes, 24 mg/kg of solubilised biotin (Beyotime, #ST2051) was injected intraperitoneally once daily for 3 consecutive days^161^. Mice were then euthanised, and lymph nodes were harvested for isolation of HECs. Primary HECs from metastatic LNs of mice bearing secreted SEMA3C-TurboID- or TurboID-overexpressing EO771 were isolated as described above and lysed by RIPA buffer (Beyotime, #p0013b) with 1× protease inhibitor cocktail (1:100, Thermo Fisher Scientific, #78446). Lysates were clarified by centrifugation at 20,000 × *g* at 4 °C for 10 min to collect the supernatant. Biotinylated proteins were isolated with streptavidin beads (Invitrogen, #11205D) according to the manual instructions. After washing with PBS containing 0.1% BSA, the beads were subjected to trypsin proteolysis at 37 °C overnight. The eluate was collected, and beads were washed twice in 100 μl of 50 mM ammonium bicarbonate. The combined eluate was lyophilised, then resuspended in 0.1% formic acid for mass spectrometry analysis^162^. For LC-MS/MS analysis, 200 ng of peptides from each sample were analysed using a Vanquish Neo UHPLC system coupled to an Orbitrap Astral mass spectrometer (Thermo Fisher Scientific). Briefly, the sample was first trapped on a PepMap Neo Trap Cartridge trapping column (300 μm × 5 mm, 5 μm) and subsequently separated on an Easy-Spray™ PepMap™ Neo UHPLC column (150 μm × 15 cm, 2 μm) at a flow rate of 2.5 μl/min with a column temperature of 55 °C. Mobile phase A was 0.1% formic acid, and mobile phase B was formic acid/acetonitrile (0.1/99.9, v/v). Data were acquired in data-independent acquisition (DIA) mode with a resolution of 240,000 at m/z 200, covering an MS scan range of m/z 380-980 and a target AGC value of 500%. Raw data were searched against the UniProt mouse reference proteome (uniprotkb_proteome_UP000000589_mouse_54910_20240528.fasta) using DIA-NN (v1.8.1) in library-free mode, followed by filtering at a 1% false-discovery rate (FDR).

### RNA editing analysis of tumour cells

Using Trizol (Invitrogen, #15596026) according to the manual instructions, total RNA was extracted from EO771 cells treated with vehicle (2% DMSO in saline) or 100 nM 12-HETE (Cayman, #34570) for 48 h, EO771 cells transduced with empty vectors or ADAR1 p150 overexpression vectors. Libraries were prepared with the NEB RNA Ultra Directional Kit (NEB, #E7760) and sequenced (paired-end) on the MGISEQ-2000 platform (BGI-Shenzhen, China). Read quality was checked with FastQC, duplicates removed by prinseq^163^, and sequences aligned to the mouse genome (GRCm38.91) using STAR^156^. Global RNA editing levels were measured using a published Alu-editing detection algorithm^164^. Specifically, ADAR editing converts adenosine (A) into an inosine (I), which is read by the cellular machinery as a guanosine (G)^165^. Thus, sequencing of inosine-containing RNAs results in G, where the corresponding genomic DNA reads A, known as A-to-G mismatches. The A-to-I editing index was defined as the ratio of A-to-G mismatches to the total number of nucleotides. Hyper-editing analysis, a complementary method for estimating global RNA editing levels, was applied to identify heavily edited reads that fail to align with standard genome alignment tools. To achieve this, all adenosines in both unmapped reads and the reference genome were converted to guanosines to enable alignment^166^. After realignment, the reads were reverted, and mismatches were assessed. Sites overlapping known SNPs were excluded, and repetitive regions were annotated using RepeatMasker (v4.09). For differential A-to-I editing analysis, only sites detected in at least two samples were retained to generate a high-confidence set of editing events. For mRNA expression analysis, the standard process was performed as aforementioned and then GSEA was performed using ClusterProfiler. RNA-seq data are deposited in GEO under accession GSE217454.

### Synthesis of duplex RNAs

We used a SINE repeat derived from the *Oip5* 3′UTR identified by our RNA-seq as an endogenous dsRNA substrate of ADAR1 p150. The sequence was: CCTTTAATCCCAACACTCTGGAGGCAGAAGTGGGCAGATAGATCAGTTCATGACCAGCCTGATCTACAGAGTGAGTTCTAGGACAGACAAGTCTACACAG. The dsRNAs fluorescently labelled with or without Cy5 were synthesised by Shanghai GeneBio Co., Ltd.

### Expression and purification of recombinant ADAR1 p150

His_6_-MBP-3C tagged full-length wild-type ADAR1 p150 (RefSeq NP_001139768.1), ADAR1 p150 (V669A), ADAR1 p150 (V677A), ADAR1 p150 (L681A), ADAR1 p150 (V677A/L681A), and ADAR1 p150 (E861A) were synthesised as gene fragments (Genechem), assembled into CMV-MCS-EGFP-SV40-Neomycin vector (Genechem) digested with XhoI and KpnI and confirmed by DNA sequencing. The 3C sequence serves as a PreScission protease cleavage site, while the maltose-binding protein (MBP) tag was incorporated to facilitate higher protein expression. His_6_-3C tagged truncated ADAR1 p150 (residues 1–250), truncated ADAR1 p150 (residues 251–1178), wild-type ADAR1 p110 (RefSeq NP_001033676.2) and ADAR1 p110 (V429A/L433A) fused with a C-terminal EGFP sequence were synthesised as gene fragments (Genechem), assembled into CMV-T7 promoter-MCS vector (Genechem), digested with NcoI and HindIII and confirmed by DNA sequencing. A linker of tobacco etch virus (TEV) protease cleavable site was inserted after the ADAR1 p150 sequence. PCR primers are provided in Supplementary Dataset 4.

His_6_-3C tagged ADAR1 p150 (residues 1–250), ADAR1 p150 (residues 251–1178), wild-type ADAR1 p110 and ADAR1 p110 (V429A/L433A) were expressed in *E. coli* strain BL21 (DE3) (Takara, #9126). Bacteria were grown in LB supplemented with kanamycin (Sigma Aldrich, #BP861) at 37 °C and induced with 1 mM IPTG (Thermo Fisher Scientific, #15529019) at OD600 0.8 for 16 h at 18 °C. His_6_-MBP-3C tagged full-length wild-type and mutant ADAR1 p150 were expressed in HEK293T cells transfected with Lipofectamine 3000 (Thermo Fisher Scientific, #L3000001). After 48 h, bacterial and mammalian cells were collected by centrifugation and lysed in xTractor Buffer (Takara, #635656). Lysates were clarified at 50,000 × *g* for 30 min at 4 °C, and supernatants were purified using the Capturem His-Tagged Purification Maxiprep Kit (Takara, #635713). Buffer exchange and concentration were performed with Slide-A-Lyzer MINI Dialysis Devices (Thermo Fisher Scientific, #88401) against 50 mM Tris (pH 7.5, Thermo Fisher Scientific, #15567027), 500 mM NaCl, 1% glycerol (Thermo Fisher Scientific, #17904), 0.1% CHAPS (Sigma Aldrich, #C3023) and 0.5 mM DTT (Thermo Fisher Scientific, #R0861). Protein concentration was determined using an ND-2000C, and the purified proteins were then stored at −80 °C.

### In vitro phase separation assay

An in vitro phase separation assay was performed as we previously described with slight modifications^28^. Prior to experiments, the recombinant protein stock was centrifuged at 8000 × *g* for 5 min to eliminate residual aggregates. Proteins and Cy5-dsRNA were mixed with the indicated concentration in the buffer of 25 mM HEPES (pH 7.4, Thermo Fisher Scientific, #15630106), 0.5 mM DTT (Thermo Fisher Scientific, #R0861), 150 mM NaCl for 10 min at 37 °C. PreScission protease (Sigma Aldrich, #GE27-0843-01) was always present in the samples at a ratio of 1:50 to cleave off the His_6_ or His_6_-MBP tag. In some experiments, 100 nM 12-HETE (Cayman, #34570) was added to the reaction buffer. After being transferred to a 96-well high-content screening microplate (Cellvis, #P96-0-N) with a glass bottom, the reaction samples were centrifuged for 5 min at 4000 × *g* to sediment droplets.

### In vitro fluorescence recovery after photobleaching

In vitro fluorescence recovery after photobleaching was performed as we previously described^28^. ADAR1 p150-dsRNA droplets were formed in a physiological buffer containing 25 mM HEPES (pH 7.4), 0.5 mM DTT (Thermo Fisher Scientific, #R0861), 150 mM NaCl. FRAP experiments were performed on a laser-scanning confocal microscopy (LSM880, Zeiss) at 25 °C. Three regions of interest (ROIs) were defined for these experiments. ROI-1 corresponded to the circular region subjected to photobleaching, ROI-2 was a similarly sized unbleached region, and ROI-3 was a circular region outside the droplets that was defined as background. The droplets that were ~2 μm in diameter were photobleached with 100% laser power of 488-nm and 5% laser power of 561 nm lasers for 1 s. Time-lapse imaging was performed for 1 min following bleaching, with images acquired every 3 s. Images were analysed by ImageJ. The frame immediately after photobleaching was defined as time 0. The intensities within ROI-1 during recovery were corrected by ROI-3 and normalised to pre-bleached intensities by ROI-2. The corrected and normalised values were fit to the single exponential growth curve in GraphPad Prism 10 to calculate the half-time and recovery ratio according to the equation: *I*_*t*_ *= I*_min_ + *(I*_max_*-I*_min_*)(1-e*^−^^*kt*^*)* where *I*_max_ was the plateau intensity, *I*_min_ was the intensity at *t* = 0. *k* was the exponential constant. For the recovery curve, points are shown as mean ± SD^131^.

### OptoIDR assay

The optoIDR assay was performed as previously described^79^. Briefly, the mCherry-Cry2 and IDR (ADAR1 p150_1-250_)-mCherry-Cry2 expression lentivirus were constructed by Genechem Company (Shanghai, China) and transduced into HEK293T cells at an MOI of 10 as described above. Cells were transferred to a 96-well high-content screening microplate with a glass bottom (Cellvis, #P96-0-N) for another 24 h. Confocal images were acquired on a Zeiss LSM880. The droplet formation was induced with 488 nm light pulses every 2 s, while mCherry was excited with 561 nm light. During the imaging, a series of images was captured every 2 s.

### Colocalisation of ADAR1 p150 and dsRNA in EO771 cells

Endogenously GFP-tagged ADAR1 p150 EO771 cells were grown on a 24-well high-content screening microplate with a glass bottom (Cellvis, #P24-0-N) and treated with 100 nM 12-HETE (Cayman, #34570) or 12-HETE with 3% 1,6-hexanediol (Sigma Aldrich, #240117) for 48 h. After being washed, cells were fixed, permeabilised, and stained with J2, the primary antibody (1:100, Sigma Aldrich, #MABE1134), overnight at 4 °C, and then, the anti-mouse Alexa-555 secondary antibody (1:300, Thermo Fisher Scientific, #A31570) for 1 h at room temperature. Confocal images were acquired on a Zeiss LSM880. Colocalised puncta were counted using the FIJI 3D Objects Counter plugin. Puncta from 10 cells of each independent experiment were analysed.

### Molecular dynamics simulations

Classical molecular dynamics simulations were performed using GROMACS-2020 on local GPU resources^167^. The dsRBD3 of mouse ADAR1 structure was generated with AlphaFold^168^. The topologies of 12-HETE were prepared using Acpype^169^. Simulations employed the AMBER99SB-ILDN forcefield^170^ with TIP3P waters and a chloride ion for neutralisation. Energy minimisation was achieved via the steepest-descent gradient method. Temperature (310 K) and pressure (1.0 bar) were controlled with the modified Berendsen thermostat^171^ and semi-isotropic Parrinello-Rahman barostat^172^, respectively. Bond lengths were constrained by the LINCS algorithm^173^ and long-range electrostatic forces were calculated using the particle-mesh Ewald method (PME)^174^. Short-range electrostatic and van der Waals interactions were truncated at 1.4 nm. The simulations were integrated with a leap-frog algorithm using a 2 fs time step. Structures were saved every 0.1 ns for all simulations. The final structure was generated and visualised using PyMOL 1.7.0.0 (https://pymol.org).

### ITC

The ITC experiments were carried out using the Malvern ITC200 instrument at 25 °C. The ADAR1 p150 protein and 12-HETE were diluted into the buffer of 20 mM sodium phosphate buffer (pH 7.0) and 100 mM NaCl. The titrations of 12-HETE into ADAR1 p150 protein were measured with nineteen 2 µL injections of 500 µM 12-HETE into 50 µM of ADAR1 p150 protein with 150 s intervals. The ITC thermograms were analysed and fit using the Microcal Analysis software (Origin 7.0).

### The lipid-protein overlay assays

The lipid-protein overlay assays were carried out as previously described^175^. In brief, 1 µl 12-HETE (Cayman, #34570) of the selected dilutions (62.5, 125, 250, 500 pmol) solubilised in chloroform (Sigma Aldrich, #C2432) were spotted onto nitrocellulose membranes (neoLab, #VL-0462). After air-drying for 1 h at room temperature, membranes were blocked in 50 mM Tris-HCL (pH 7.5), 150 mM NaCl, 2 mg/ml fatty acid-free BSA (Sigma Aldrich, #10775835001) and 0.1% Tween 20 (Sigma Aldrich, #9005-64-65) for 1 h at room temperature, followed by incubation with 2 mg/ml indicated ADAR1 p150 protein constructs fused with GFP expression in fresh blocking buffer overnight at 4 °C. Pure GFP was used as a negative control. After extensive washing with TBS-T (10 cycles over 50 min), bound proteins were incubated with anti-GFP antibody (1:10,000, Abcam, #ab183734) overnight, followed by HRP-conjugated anti-rabbit IgG (1:3000, CST, #7074) for 1 h. Signals were visualised with ECL (Thermo Fisher Scientific, #32106) on a Syngene G:BOX imaging system.

### EMSA

ADAR1 p150 protein and dsRNA with the indicated concentration were mixed with or without 12-HETE in 20 mM Tris-HCl (pH 7.5) and 100 mM NaCl on ice for 20 min. The mixtures were loaded onto the 1% agarose gel containing 1× SYBR Gold (Thermo Fisher Scientific, #S11494) and electrophoresed in TBE buffer at 5 V/cm for 40 min. Gels were visualised with a gel imaging system (Syngene G:BOX). The quantification of free and ADAR1 p150-bound dsRNA was carried out with ImageJ, and the K_D_ values were calculated by fitting the binding curves to a Hill equation in GraphPad Prism 10 software.

### ADAR1 p150 thermal shift assay

The thermal shift assay was conducted using a 7500 Real-Time PCR System (Applied Biosystems) with its Melt Curve option. Recombinant ADAR1 p150 was mixed with or without dsRNA or 100 nM 12-HETE (Cayman, #34570) in the Protein Thermal Shift Buffer supplemented with a Protein Thermal Shift Dye (Thermo Fisher Scientific, #4461146). Samples were heated from 25 °C to 99 °C at a 1% Ramp rate. The *T*_m_ values were determined by fitting the melting curves to a Boltzmann sigmoidal equation in GraphPad Prism 10 software.

### In vitro ADAR1 editing activity assays

Briefly, 20 nM indicated recombinant ADAR1 proteins loaded with 20 nM dsRNA were diluted into the buffer of 20 mM HEPES (pH 7.9), 0.1 M KCl, 20% Glycerol and 0.2 mM EDTA (Sigma Aldrich, #E9884). Phase separation was induced by adding an equal volume of 100 nM 12-HETE (Cayman, #34570) at 37 °C. Reactions were quenched at 5 min intervals with 0.5 μl of 0.5 M EDTA. Proteins were digested with Proteinase K (Thermo Fisher Scientific, #25530049) for 1 h at 37 °C, and edited dsRNAs were purified by phenol/chloroform extraction and precipitation. Purified RNAs were reverse-transcribed (TaKaRa, #RR037B) and cDNA was analysed by Sanger sequencing (Sangon Biotech).

### ADAR1 editing dual reporter assay

Three reporters were synthesised by Genechem Company (Shanghai, China). The R/G editing and Nanoluciferase (Nluc) sequences were inserted into the vector following the firefly luciferase gene by homologous recombination. A positive control was generated by mutating the editing site to G to mimic complete editing, while an 18-nt deletion in the complementary sequence produced a negative control^83^. EO771 cells were transfected with negative (0% editing), positive (100% editing), and ADAR1 editing reporter lentivirus as described above. Reporter activity was measured using Nano-Glo Dual-luciferase Reporter Assay System (Promega, #N1610). Relative response ratio (RRR) was calculated as: (experimental editing assay ratio-negative control ratio) / (positive control ratio-negative control ratio).

### Droplet and bulk concentration measurement

To measure the concentrations of ADAR1 p150 and dsRNA in the drop and bulk phase, fluorescence images of ADAR1 p150-dsRNA droplets were acquired with a Zeiss LSM880 confocal microscope (63× objective). For each of six samples, five droplets were measured to obtain fluorescence intensities. Bulk intensities were determined by centrifuging phase-separated samples at 22,000 × *g* for 30 min at 22 °C. Then the resulting supernatants were transferred to adjacent wells for imaging. A dilution series of protein and dsRNA was used to yield a standard curve. A custom script was used to analyse the fluorescence images in MATLAB as previously reported^176^. Briefly, droplets and bulk were segmented, and their mean intensity was extracted and converted into concentrations using the corresponding standard curves.

### Volume-normalised editing rate measurement

To quantify the volume-normalised editing rates, both editing activity and phase volumes were measured. Briefly, the droplet and bulk fraction were separated by centrifugation at 22,000 × *g* for 30 min at 22 °C. The total ADAR1 p150 editing activity in the total mixture and bulk editing activity in the bulk phase were measured by Sanger sequencing (Sangon Biotech) as described above. Droplet activity in the droplet was determined by total editing activity and bulk editing activity:

$${{{\rm{droplet}}}}\; {{{\rm{activity}}}}={{{\rm{total}}}}\; {{{\rm{activity}}}}-{{{\rm{bulk}}}}\; {{{\rm{activity}}}}$$

Assuming that droplet formation doesn’t change total volume, we calculated the droplet volume by measuring the concentration in total, droplet and bulk as described above. Based on conservation of mass, droplet volume fraction was determined by the total, droplet and bulk concentrations:

$${{{\rm{droplet}}}}\; {{{\rm{volume}}}}\; {{{\rm{fraction}}}}=\frac{{{{\rm{total}}}}\; {{{\rm{concentration}}}}-{{{\rm{bulk}}}}\; {{{\rm{concentration}}}}}{{{{\rm{droplet}}}}\,{{{\rm{concentration}}}}-{{{\rm{bulk}}}}\; {{{\rm{concentration}}}}}$$

The total reaction volume is 20 µl. Therefore, bulk volume fraction was determined by total reaction volume and droplet volume:

$${{{\rm{bulk}}}}\; {{{\rm{volume}}}}\; {{{\rm{fraction}}}}={{{\rm{total}}}}\; {{{\rm{volume}}}}-{{{\rm{droplet}}}}\; {{{\rm{volume}}}}\; {{{\rm{fraction}}}}$$

Additionally, we carried out reactions at the concentrations measured within the droplets with 270 nM dsRNA and 530 nM truncated ADAR1 p150 (residues 251–1178), which ADAR1 p150 and dsRNA that lost the ability to undergo LLPS and measured the activity in these droplets equivalent concentration (DEC) reactions. Finally, we calculated the volume-normalised editing rates of different reactions:

$${{{\rm{volume}}}}-{{{\rm{normalized}}}}\; {{{\rm{editing}}}}\; {{{\rm{rates}}}}=\frac{{{{\rm{reaction}}}}\; {{{\rm{activity}}}}}{{{{\rm{reaction}}}}\; {{{\rm{time}}}}*{{{\rm{reaction}}}}\; {{{\rm{volume}}}}}$$

### Statistical analysis

Statistical analyses were performed with Prism 10 (GraphPad Software). Normality and variance were tested before applying appropriate statistical tests, as noted in figure legends and “Methods” section. Data are presented as mean ± SD, with significance defined as *P* < 0.05. For multiple comparisons, *P*-values were corrected using the Benjamini-Hochberg FDR method. n.d., not detected. Sample sizes were not predetermined but were consistent with prior studies. All data represent at least three independent experiments. For all in vivo experiments, animals were randomly assigned to experimental groups and age-matched. All experiments were blinded, where possible.

### Reporting summary

Further information on research design is available in the Nature Portfolio Reporting Summary linked to this article.

## Supplementary information

Supplementary information

Description of Additional Supplementary Files

Supplementary Dataset 1

Supplementary Dataset 2

Supplementary Dataset 3

Supplementary Dataset 4

Supplementary Movie 1

Reporting Summary

Transparent Peer Review file

## Source data

Source data

## Data Availability

RNA-seq and scRNA-seq datasets generated in this study have been deposited in GEO under accession number GSE217454, and whole-exome sequencing data are available in SRA under accession number PRJNA1024076. The mass spectrometry proteomics data have been deposited to the ProteomeXchange Consortium (https://proteomecentral.proteomexchange.org) via the iProX partner repository with the dataset identifier PXD074282. All experimental materials and protocols are detailed in the “Methods” section, with relevant references cited. Restrictions apply to the availability of the medical training/validation data in accordance with hospital requirements governing human subject privacy protection, and so are not publicly available. Any additional data can be provided by the corresponding author upon request (sushch@mail.sysu.edu.cn), subject to approval from Sun Yat-Sen Memorial Hospital. The raw numbers for charts and graphs are available in the Source Data file. Source data are provided with this paper.

## Code availability

All original code related to deep learning models has been deposited at https://github.com/xiaqd/HEVI-detection. and archived at Zenodo: 10.5281/zenodo.18824122.
