# Supplementary material for: Tumour-associated high endothelial venules drive portal-specific immune evasion in lymph nodes via ALOX12
Source: Nat Commun. 2026 May 11;17:6364. doi: 10.1038/s41467-026-72412-w (PMC13376640; doi:10.1038/s41467-026-72412-w)
Supplement: Supplementary file 1 — Supplementary information [file 41467_2026_72412_MOESM1_ESM.pdf]

Supplementary Information

Tumour-associated high endothelial venule drive portal-specific immune evasion in lymph nodes via ALOX12

Author: Qidong Xia, Jiayao Pan, Xiaoqi Weng, Shunrong Li, Jiaqian Li, Shijian Song, Jiang Li, Boxuan Zhou, Xinwei Liu, Dong-Ming Kuang, Nu Zhang, Jin Jin, Jinting Liu, Zhen Lin, Shubin Yu, Qionglan Tang, Lijuan Bian, Yunjie Zeng, Yu Shi, Yiqing Zheng, Jian-You Liao*, Shouping Xu*, Shicheng Su*

Contents

Supplementary Fig. 1 | HEV involvement stratifies the prognosis of patients with LN-positive breast cancer.
Supplementary Fig. 2 | Selection of derivatives that preferentially spread via HEVs.
Supplementary Fig. 3 | HEV-emigrant tumour cells evade immune surveillance.
Supplementary Fig. 4 | HEV-emigrant tumour cells stimulate a weaker immune response in mediastinal LNs.
Supplementary Fig. 5 | ALOX12 is mainly expressed in tumour-associated HECs.
Supplementary Fig. 6 | ALOX12 is predominantly expressed in HECs rather than non-HEV LN BECs in LNs.
Supplementary Fig. 7 | Tumour-associated HEV-derived ALOX12 suppresses the immunogenicity of tumour cells via 12-HETE.
Supplementary Fig. 8 | Tumour-derived SEMA3C upregulates ALOX12 in tumour-associated HEVs.
Supplementary Fig. 9 | 12-HETE-induced RNA editing appears to be the primary mechanism underlying immune suppression.
Supplementary Fig. 10 | The effect of 12-HETE on immune evasion is dependent on ADAR1.
Supplementary Fig. 11 | 12-HETE enhances ADAR1 p150-dsRNA liquid-liquid phase separation.
Supplementary Fig. 12 | ADAR1 p150-dsRNA liquid-liquid phase separation induced by 12-HETE promotes A-to-I RNA editing.
Supplementary Fig. 13 | Schematic highlighting the primary finding of this study.
Supplementary Table 1. Association between HEV involvement and clinical features in the SLN-positive cohort from the Sun Yat-Sen Memorial Hospital (n = 559).
Supplementary Table 2. Cox regression analysis of DFS in the SLN-positive cohort from the Sun Yat-Sen Memorial Hospital (n = 559).
Supplementary Table 3. Cox regression analysis of OS in the SLN-positive cohort from the Sun Yat-Sen Memorial Hospital (n = 559).
Supplementary Table 4. Mutation status of EO771, Py230 and B16F10 cell lines.

Supplementary Fig. 1

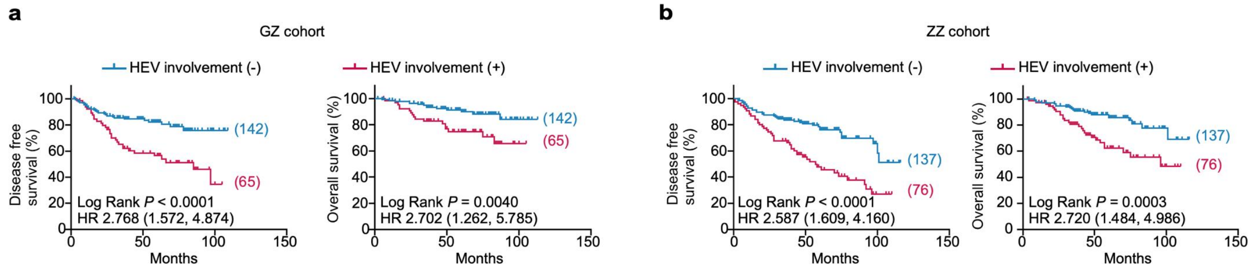

Supplementary Fig. 1 | HEV involvement stratifies the prognosis of patients with LN-positive breast cancer. a, **b**, Kaplan-Meier analysis for disease-free survival and overall survival in patients with LN-positive breast cancer with or without HEV involvement from the Affiliated Hospital of Guizhou Medical University (GZ cohort) (a) and the First Affiliated Hospital of Zhengzhou University (ZZ cohort) (b). *P* values were calculated by the log-rank Mantel-Cox test. HR, hazard ratio.

Supplementary Fig. 2

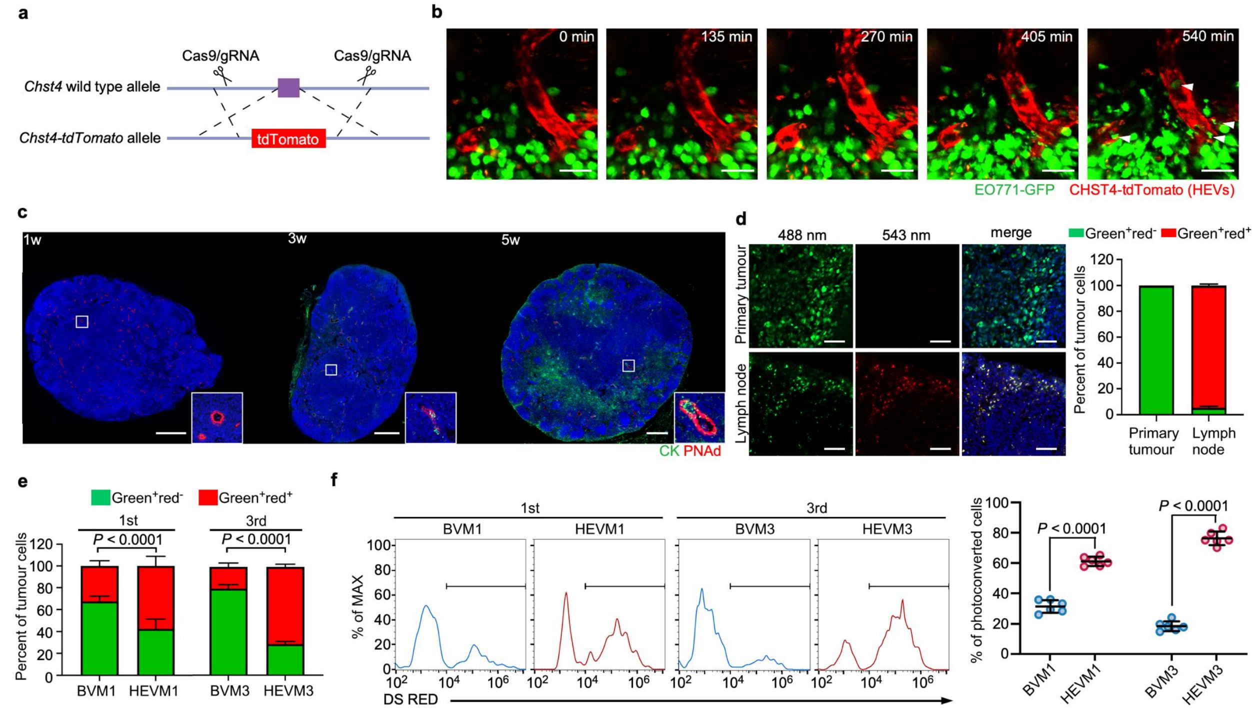

Supplementary Fig. 2 | Selection of derivatives that preferentially spread via HEVs. a, Construction diagram of *Chst4-tdTomato* mice. **b**, Time-lapse intravital multiphoton images of GFP-expressing EO771 cells (green) and HEVs (red) over the course of 540 min. Images were showed at 0, 135, 270, 405, 540 min. Arrowheads indicate HEV-intravasating tumour cells. Scale bars, 50 µm. **c**, Representative immunofluorescence staining for CK and PNAd in draining LNs of mice at indicated time points after EO771 cells inoculation in mammary fat pads. Scale bars, 500 µm. **d**, Mice inoculated with Dendra2H2B-EO771 cells in mammary fat pads had their LNs photoconverted with a 405-nm diode. Representative images (left) and quantitation (right) of green^{+}red^{-} and green^{+}red^{+} tumour cells are shown in primary tumours and LNs. Scale bars, 50 µm. **e**, The quantitation of green^{+}red^{-} tumour cells and green^{+}red^{+} tumour cells in Fig. 2d. **f**, Representative flow cytometry plots (left) and quantitation (right) of photoconverted cells in the lung single-cell suspensions from mice inoculated with 1st generation cells (EO771-BVM1 and EO771-HEVM1) or 3rd generation cells (EO771-BVM3 and EO771-HEVM3).

n = 6 mice per group (d-f). Mean ± SD (d-f). *P* values were calculated by two-tailed unpaired *t*-test (e, f).

Supplementary Fig. 3

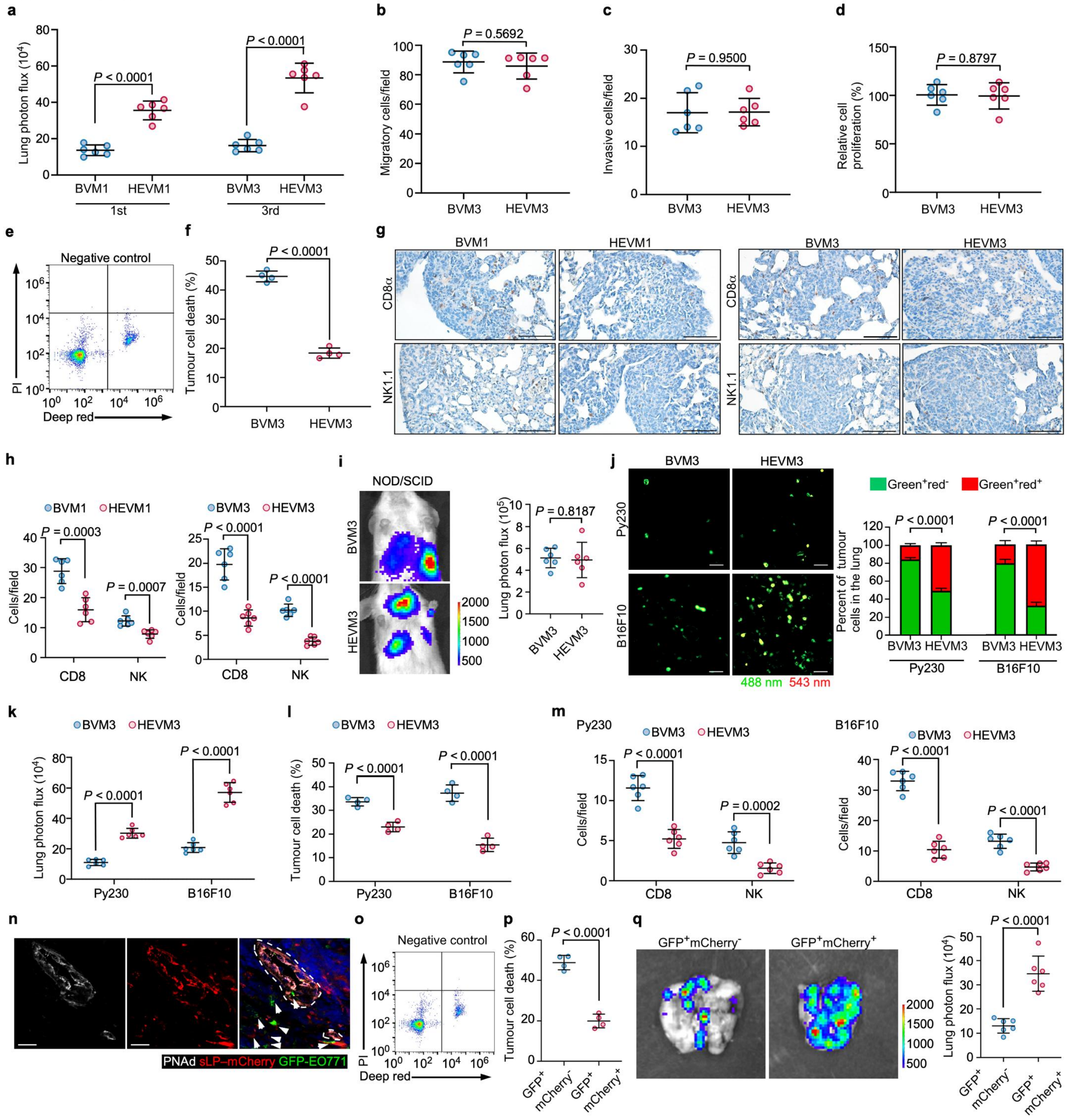

Supplementary Fig. 3 | HEV-emigrant tumour cells evade immune surveillance. a, The quantitation of Fig. 2e. **b**, **c**, The quantitation of EO771-BVM3 cells and EO771-HEVM3 cells in the migration assay (b) and the invasion assay (c). Each dot represents data pooled from 5 microscopic fields of each independent experiment. **d**, Cell proliferation rates were analyzed using the MTT assay comparing the EO771-BVM3 cells and EO771-HEVM3 cells. **e**, Unstimulated T cells were used as a negative control of Fig. 2f. **f**, The quantitation of Fig. 2f. **g**, **h**, Representative images (g) and the quantitation (h) of immunohistochemistry staining for CD8α and NK1.1 in the lung metastases of mice inoculated with indicated EO771 derivatives. Scale bars, 100 μm. Each dot represents data pooled from 5 distinct areas from a slice and 3 slices per mouse. **i**, Representative bio-luminescent images (left) and quantitation (right) of

NOD/SCID mice inoculated with luciferase-expressing EO771-BVM3 cells or EO771-HEVM3 cells. **j**, We inoculated BVM3 or HEVM3 derivatives from Py230 and B16F10 cells in mammary fat pads and flank skin into syngeneic mice respectively. We had the draining LNs photoconverted with a 405-nm diode. Representative immunofluorescence images of lungs (left) and the quantitation (right) showing photoconverted tumour cells with both green and red fluorescence. Scale bar, 50 μm. **k**, The quantitation of lung photon flux of C57BL/6 mice inoculated with indicated luciferase-expressing Py230 cells via fat pad injection and indicated luciferase-expressing B16F10 cells via intradermal injection. **l**, The quantitation of $CD8^+$ T cell-mediated cytotoxicity to indicated cells. Cell tracker Deep Red-labeled tumour cell death was determined by PI staining and detected by flow cytometry *in vitro*. Numerical values denote the percentage of dead tumour cells. **m**, The quantitation of immunohistochemistry staining for CD8α and NK1.1 in the lung metastases of mice inoculated with indicated cells. Each dot represents data pooled from 5 distinct areas from a slice and 3 slices per mouse. **n**, Representative immunofluorescence images show labelled luciferase-GFP-EO771 cells ($GFP^+mCherry^+$) in close proximity to HEVs in lymph nodes of *Chst4-CreERT2*;*R26-LSL-sLP-mCherry* mice inoculated with luciferase-GFP-EO771 cells. HEVs were further delineated by PNAd antibody staining (white dashed lines). Scale bar, 20 μm. **o**, Unstimulated T cells were used as a negative control of Fig. 2i. **p**, The quantitation of Fig. 2i. **q**, Representative bio-luminescent images (left) and quantitation (right) of lungs harvested from mice inoculated with indicated luciferase-expressing EO771 cells via fat pad injection.

n = 6 mice per group (a, h-k, m, q); n = 6 independent experiments (b-d); n = 4 independent experiments (f, l, p). Mean ± SD (a-d, f, h-m, p, q). *P* values were calculated by two-tailed unpaired *t*-test (a-d, f, h-m, p, q).

Supplementary Fig. 4

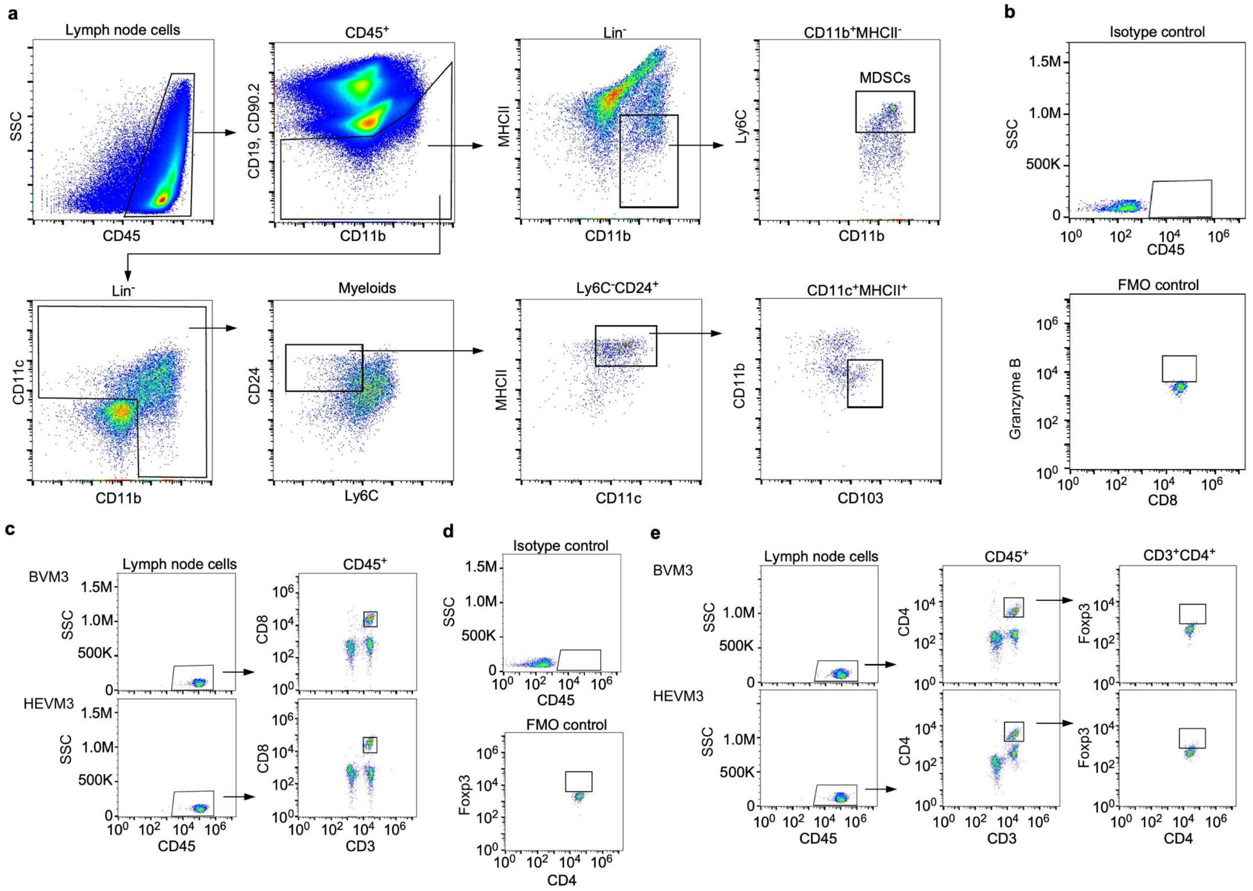

Supplementary Fig. 4 | HEV-emigrant tumour cells stimulate a weaker immune response in mediastinal LNs. **a**, Flow cytometry gating strategy of migratory CD103$^+$ DCs and MDSCs from mediastinal LNs of BVM3 and HEVM3-bearing mice. Lin$^-$, lineage negative. **b**, An isotype-matched IgG antibody was used as a negative control for CD45 gating (top). A FMO control was used to define the threshold for Granzyme B staining in CD45$^+$CD3$^+$CD8$^+$ T cells (bottom). **c**, Flow cytometry gating strategy of Granzyme B$^+$ CD8$^+$ T cells in mediastinal LNs of BVM3 and HEVM3-bearing mice. **d**, An isotype-matched IgG antibody was used as a negative control for CD45 gating (top). A FMO control was used to define the threshold for Foxp3 staining in CD45$^+$CD3$^+$CD4$^+$ T cells (bottom). **e**, Gating strategy and representative flow cytometry plots of Treg cells (Foxp3$^+$CD4$^+$) in mediastinal LNs of BVM3 and HEVM3-bearing mice.

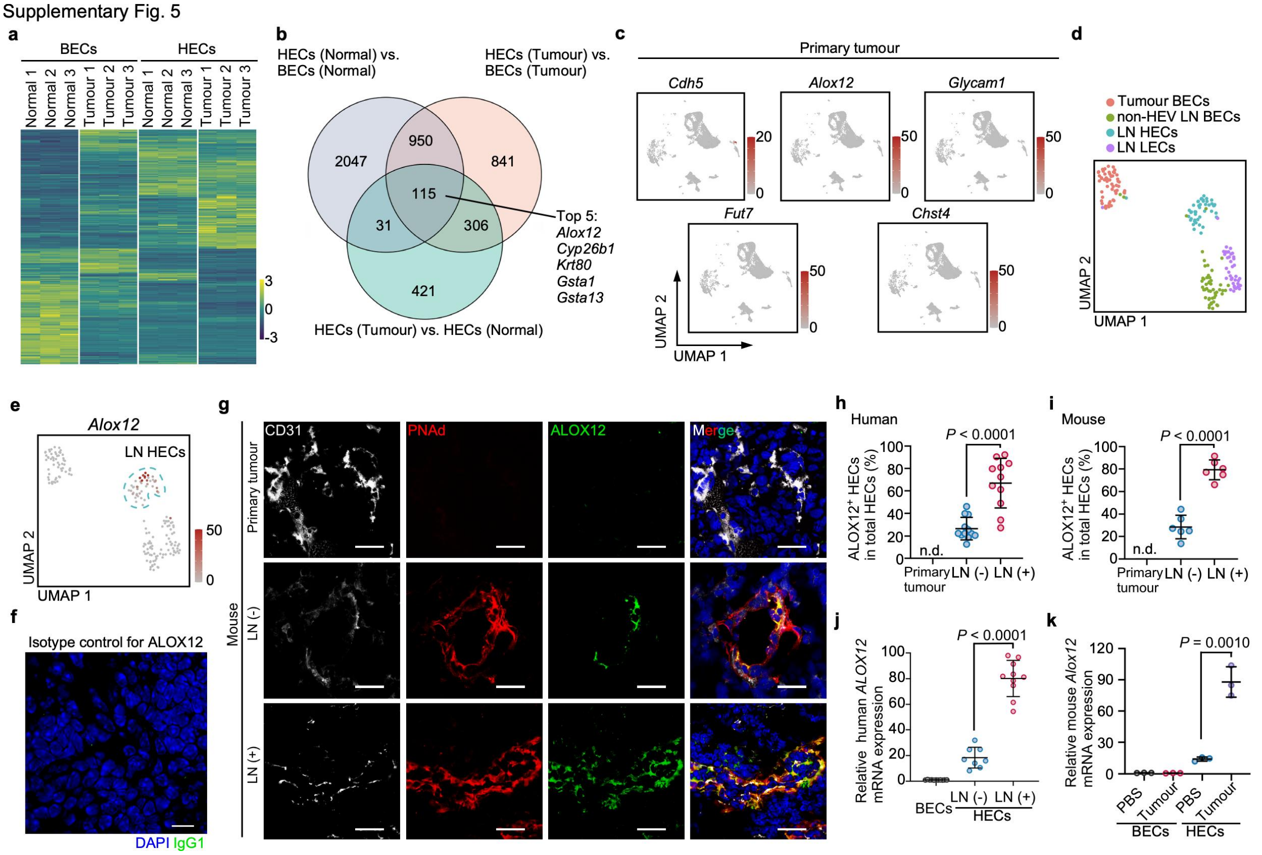

Supplementary Fig. 5 | ALOX12 is mainly expressed in HECs in tumour-associated HEVs. **a**, **b**, BECs were isolated from mammary fat pads (normal) or EO771 tumours (tumour). HECs were isolated from LNs of mice without (normal) or with (tumour) EO771 tumour inoculation in mammary fat pads. The heatmap of the RNA-seq data. Numbers are log2 transformed. The color scale is shown (a). Venn diagram shows overlap of upregulated genes in indicated comparisons. Top 5 genes ranked using Fisher's combined probability test are shown (b). **c**, UMAP plots showing the expression levels of indicated genes in EO771 primary tumours. Data pooled from 6 mice. **d**, The UMAP plot showing sub-clusters of ECs in our scRNA-seq data of primary tumour and LNs. **e**, The UMAP plot showing the expression levels of *Alox12*. **f**, An isotype-matched IgG antibody was used as a negative control for ALOX12. Scale bar, 20 µm. **g**, Representative immunofluorescence images show the colocalization of CD31, PNAd and ALOX12 in EO771 tumours, mouse LNs without (LN^-) or with (LN^+) metastases. Scale bars, 20 µm. **h**, The quantitation of the proportion of ALOX12$^+$ HECs in Fig. 3d in primary tumours (n = 16 patients), LNs without (LN^-) (n = 12 patients) or LNs with metastases (LN^+) (n = 11 patients). Each dot represents data pooled from 5 distinct areas from a slice and 3 slices per patient. **i**, The quantitation of the proportion of ALOX12$^+$ HECs in Supplementary Fig. 5g in EO771 tumours, mouse LNs without (LN^-) or with metastases (LN^+). Each dot represents data pooled from 5 distinct areas from a slice and 3 slices per mouse. **j**, The levels of ALOX12 mRNA expression in human BECs from primary tumours (n = 11 patients) and HECs in LNs without (LN^-) (n = 8 patients) or with metastases (LN^+) (n = 10 patients) were assessed by qRT-PCR. **k**, Mice were injected with PBS or EO771 cells. The levels of Alox12 mRNA expression in BECs in mammary fat pads or primary tumours and HECs in LNs were assessed by qRT-PCR.

n = 3 independent experiments (a, b, k); n = 6 mice per group (i). Mean ± SD (h-k). *P* values were calculated by two-tailed unpaired *t*-test (h, i) and one-way ANOVA with Tukey's multiple comparisons test (j, k). n.d., not detected.

Supplementary Fig. 6

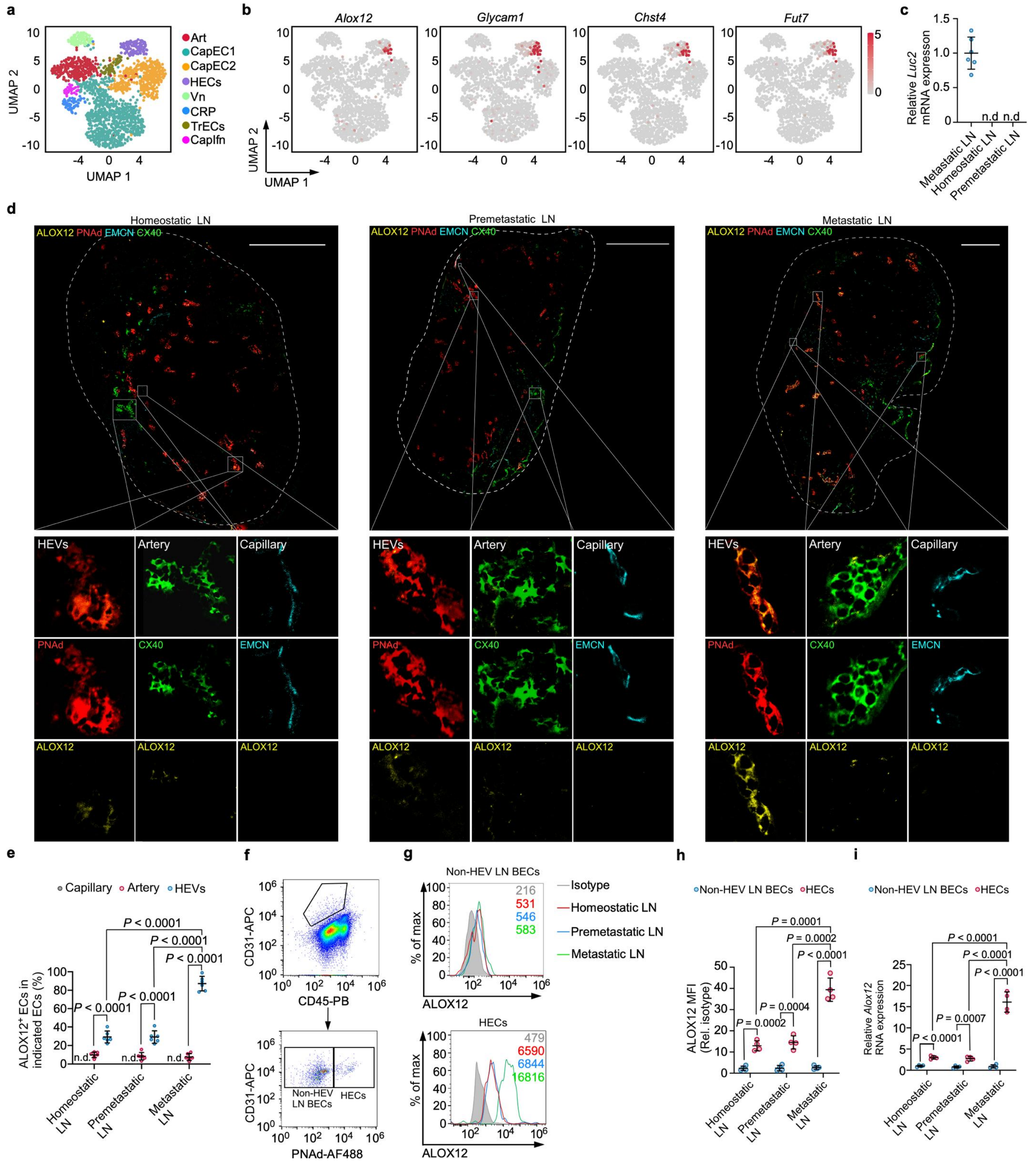

Supplementary Fig. 6 | ALOX12 is predominantly expressed in HECs rather than non-HEV LN BECs in LNs. a, The UMAP plot showing eight major subsets of the 1870 sorted BECs from metastatic LNs of 6 mice. Arterial ECs (Art), high endothelial cells (HECs), non-HEV veins (Vn), and five capillary phenotype ECs (CapEC1, CapEC2, capillary resident progenitors (CRP), transitional ECs (TrECs), interferon stimulated gene-enriched CapECs (CapIfn)). **b**, UMAP plots showing the expression levels of indicated genes in sorted BECs. HECs are identified as an endothelial cell subset expressing *Glycam1*, *Chst4* and *Fut7*. **c**, No

Luc2 signals were detected in LNs of normal mice (homeostatic) or mice 11 days after being inoculated with *Luc2*-expressing tumour cells in fat pads (pre-metastatic). *Luc2* signals were detected in LNs 2 weeks after inoculation (metastatic). **d**, **e**, Representative images (d) and the quantitation (e) of immunofluorescence staining for CX40, EMCN, PNAd and ALOX12 in homeostatic LNs, pre-metastatic LNs and metastatic LNs. Scale bars, 500 μm. Each dot represents data pooled from 3 slices of one mouse. **f**, Flow cytometry gating strategy to isolate HECs and non-HEV LN BECs from mouse LNs. **g**, **h**, Representative flow cytometry plots (g) and quantitation (h) of the level of ALOX12 in HECs and non-HEV LN BECs from mouse homeostatic LNs, pre-metastatic LNs and metastatic LNs. **i**, The expression of *Alox12* in HECs and non-HEV LN BECs isolated from mouse homeostatic LNs, pre-metastatic LNs and metastatic LNs was assessed by qRT-PCR.
n = 6 mice per group (c, e); n = 4 independent experiments with five mice per experiment (h, i). Mean ± SD (c, e, h, i). *P* values were calculated by two-tailed unpaired *t*-test (e, h, i). n.d., not detected.

Supplementary Fig. 7

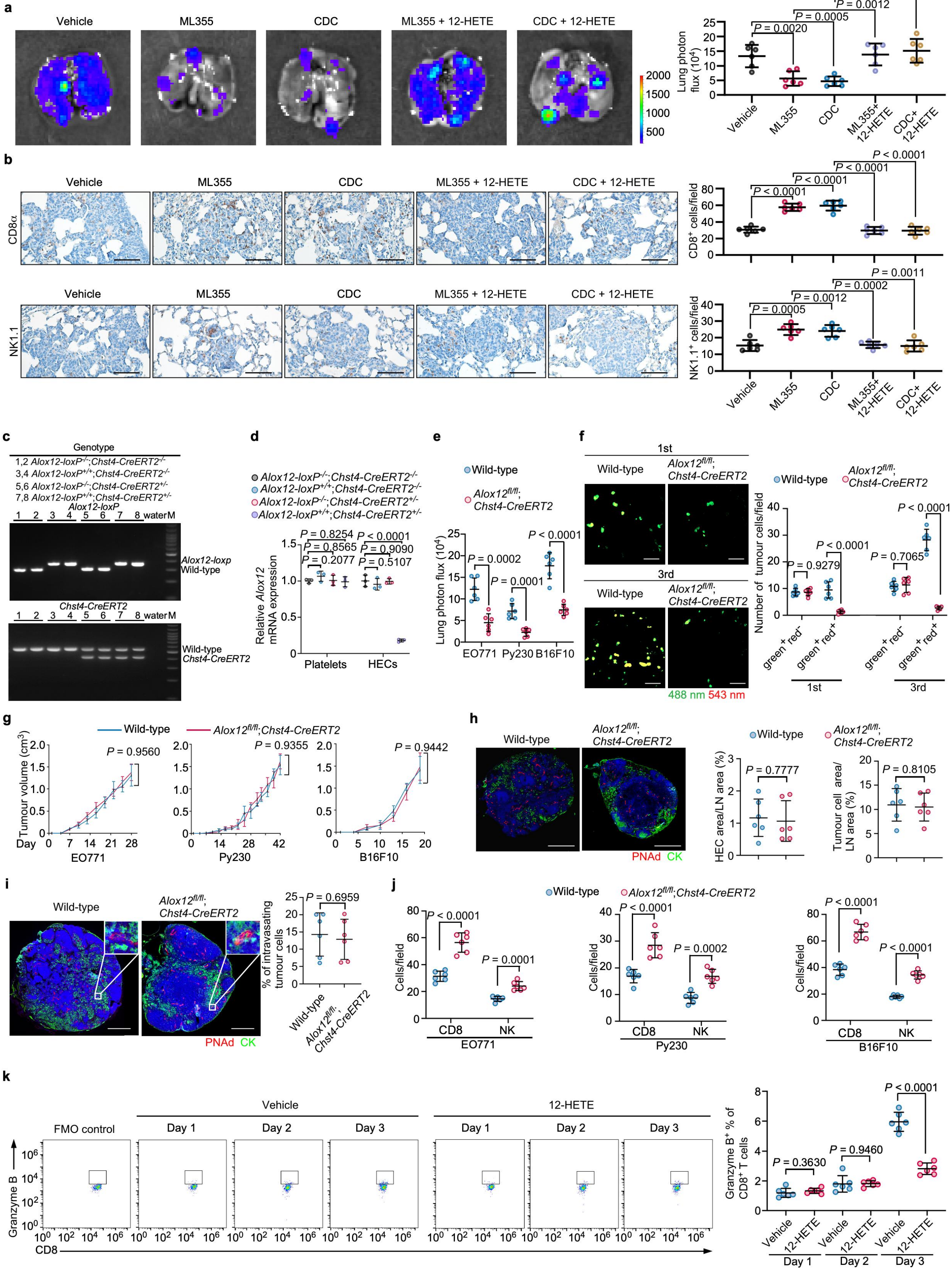

Supplementary Fig. 7 | Tumour-associated HEV-derived ALOX12 suppresses the immunogenicity of tumour cells via 12-HETE. a, Representative bio-luminescent images (left) and quantitation (right) of lungs harvested from EO771-bearing mice treated with vehicle, ML355, CDC, ML355 plus 12-HETE or CDC plus 12-HETE. **b**, Representative images (left) and the quantitation (right) of immunohistochemistry staining for CD8α and NK1.1 in the lung metastases of EO771-bearing mice treated with vehicle, ML355, CDC, ML355 plus 12-HETE or CDC plus 12-HETE. Scale bars, 100 μm. Each dot represents data pooled from 5 distinct areas from a slice and 3 slices per mouse. **c**, PCR genotyping on tail DNA of *Alox12-loxP*$^{-/-}$;*Chst4-CreERT2*$^{-/-}$ mice (1, 2), *Alox12-loxP*$^{+/+}$;*Chst4-CreERT2*$^{-/-}$ mice (3, 4), *Alox12-loxP*$^{-/-}$;*Chst4-CreERT2*$^{+/-}$ mice (5, 6) and *Alox12-loxP*$^{+/+}$;*Chst4-CreERT2*$^{+/-}$ mice (7, 8) for *Alox12-loxP* (wild-type at 302 bp and *Alox12-loxP* at 361 bp) and *Chst4-CreERT2* (wild-type at 530 bp and *Chst4-CreERT2* at 406 bp). M, marker. **d**, Levels of *Alox12* mRNA in HECs of indicated mouse models were evaluated by qRT-PCR. Mouse platelets were used as positive controls. **e**, The quantitation of lung photon flux of wild-type and *Alox12*$^{f/f}$;*Chst4-CreERT2* mice inoculated with luciferase-expressing indicated tumour cells. **f**, Representative immunofluorescence images (left) and quantitation (right) of lungs in indicated mouse bearing EO771-HEVM1 or EO771-HEVM3 cells showing photoconverted tumour cells with both green and red fluorescence. Scale bars, 50 μm. Each dot represents data pooled from 5 distinct areas from a slice and 3 slices per mouse. **g**, The tumour volume curves of wild-type and *Alox12*$^{f/f}$;*Chst4-CreERT2* mice inoculated with luciferase-expressing indicated tumour cells. **h**, Left, representative immunofluorescence staining for CK and PNAd in metastatic LNs of wild-type mice and *Alox12*$^{fl/fl}$;*Chst4-CreERT2* mice. Scale bars, 500 μm. Middle, the quantitation of relative HEC area in immunofluorescence images. Right, the quantitation of relative tumour cell area in immunofluorescence images. Each dot represents data pooled from 3 slices of one mouse. **i**, Representative immunofluorescence staining (left) for CK and PNAd in metastatic LNs of wild-type mice and *Alox12*$^{fl/fl}$;*Chst4-CreERT2* mice and quantitation of intravasating tumour cells (right). Scale bars, 500 μm. Zoomed-in images demonstrate tumour intravasation to HEVs. Each dot represents data pooled from 3 slices of one mouse. **j**, The quantitation of immunohistochemistry staining for CD8α and NK1.1 in lung metastases of mice inoculated with indicated cells. Each dot represents data pooled from 5 distinct areas from a slice and 3 slices per mouse. **k**, Representative flow cytometry plots (left) and quantitation (right) of Granzyme B^+CD8^+ T cells in mediastinal LNs of mice at the indicated time points after tail vein injection of EO771 cells pretreated with vehicle or 12-HETE.

n = 6 mice per group (a, b, e-k); n = 3 independent experiments (d). Mean ± SD (a, b, d-k). *P* values were calculated by one-way ANOVA with Tukey's multiple comparisons test (a, b, d), two-tailed unpaired *t*-test (e, f, h-k) and two-way ANOVA followed by Sidak's multiple comparisons test (g).

Supplementary Fig. 8

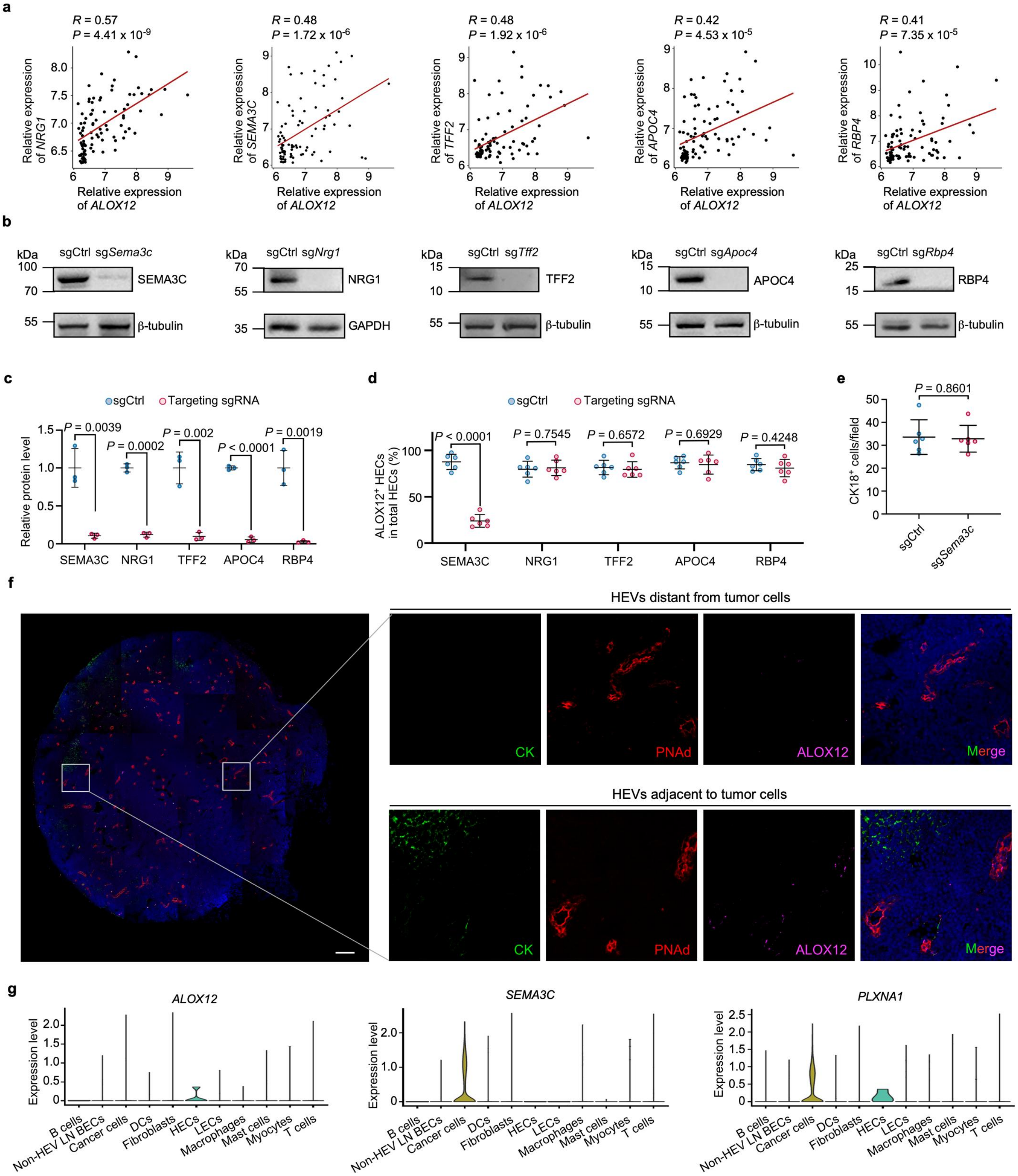

Supplementary Fig. 8 | Tumour-derived SEMA3C upregulates ALOX12 in tumour-associated HEVs. a, Correlation analysis between indicated gene expression and *ALOX12* mRNA expression in metastatic LNs from 90 breast cancer patients in the GSE32489 dataset. Spearman's correlation coefficients and *P* values are shown. **b**, **c**, Representative western blots (b) and quantitation (c) of indicated protein levels in EO771 cells transfected with indicated sgRNA plasmids. **d**, The quantitation of the proportion of ALOX12$^+$ HECs in LNs of mice injected with EO771 cells with indicated treatment. Each dot represents data pooled from 5 distinct areas from

a slice and 3 slices per mouse. **e**, The quantitation of CK^+ cells in Fig. 4a. Each dot represents data pooled from 5 distinct areas from a slice and 3 slices per mouse. **f**, Immunofluorescence analysis of ALOX12 expression in lymph nodes at an early stage of metastasis. Lymph nodes were harvested 2 weeks after tumour implantation, when only sparse metastatic tumour cells were present. Scale bar, 200 μm. **g**, Expression of *ALOX12*, *SEMA3C and PLXNA1* in different cell types identified by a publicly available scRNA-seq data set of human metastatic LNs.

n = 3 independent experiments (c); n = 6 mice per group (d, e). Mean ± SD (c-e). *P* values were calculated by two-tailed unpaired *t*-test (c-e).

Supplementary Fig. 9

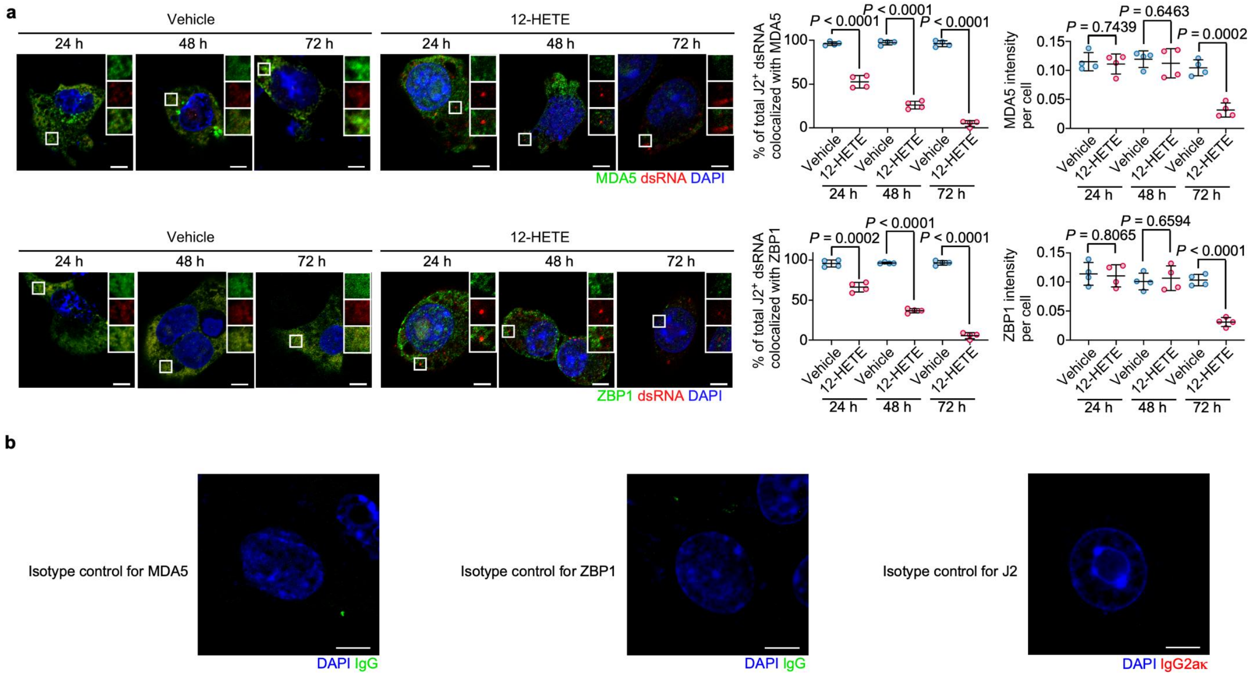

Supplementary Fig. 9 | 12-HETE-induced RNA editing appears to be the primary mechanism underlying immune suppression. **a**, Left, representative immunofluorescence staining for dsRNA (J2 antibody) and MDA5 or ZBP1 in EO771 cells with indicated treatment and at different time points. Scale bars, 5 µm. Middle, the quantification of the proportion of dsRNA signal colocalized with MDA5 or ZBP1, expressed as the percentage of total dsRNA (J2$^+$) fluorescence intensity overlapping with MDA5 or ZBP1 fluorescence in immunofluorescence images. Right, the quantitation of mean fluorescence intensity (MFI) of MDA5 or ZBP1 per cell in immunofluorescence images. **b**, Isotype control images were stained with DAPI and isotype-matched IgG antibodies and visualized using the same imaging settings. Scale bars, 5 µm.

n = 4 independent experiments (a). Mean ± SD (a). *P* values were calculated by two-tailed unpaired *t*-test (a).

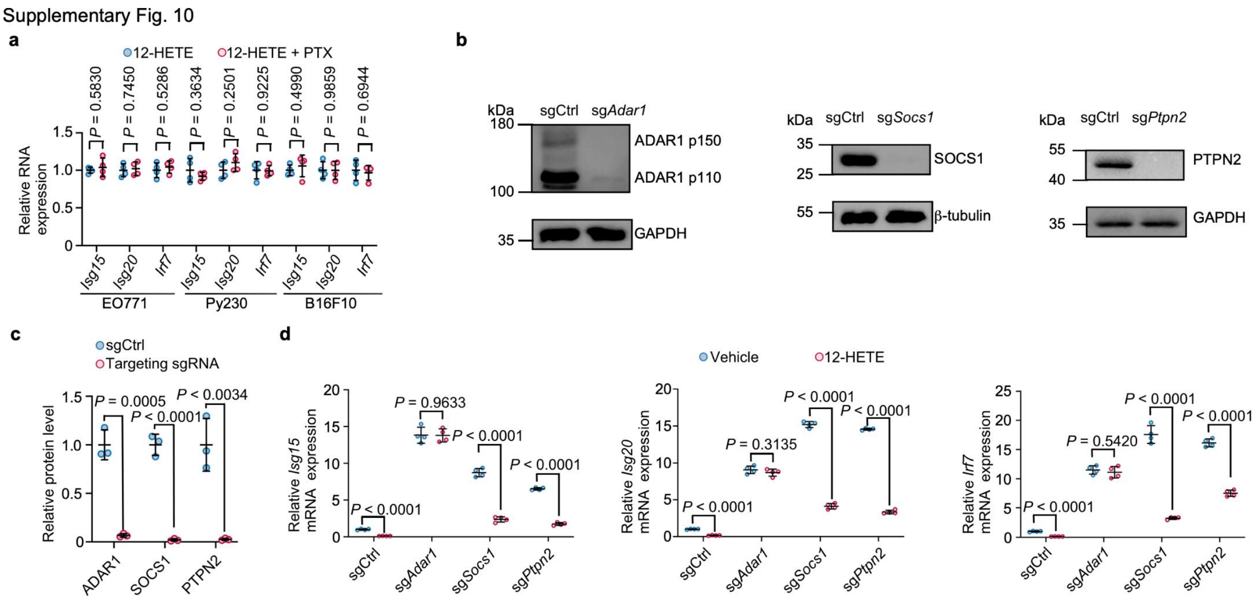

Supplementary Fig. 10 | The effect of 12-HETE on immune evasion is dependent on ADAR1. **a**, qRT–PCR analysis of indicated ISGs in indicated cells treated with 12-HETE or 12-HETE plus pertussis toxin (PTX). **b**, **c**, Representative western blots (b) and quantitation (c) of indicated protein levels in EO771 cells transfected with indicated sgRNA plasmids. **d**, qRT–PCR analysis of indicated ISGs in indicated cells treated with vehicle or 12-HETE.

n = 4 independent experiments (a, d); n = 3 independent experiments (c). Mean ± SD (a, c, d). *P* values were calculated by two-tailed unpaired *t*-test (a, c, d).

Supplementary Fig. 11

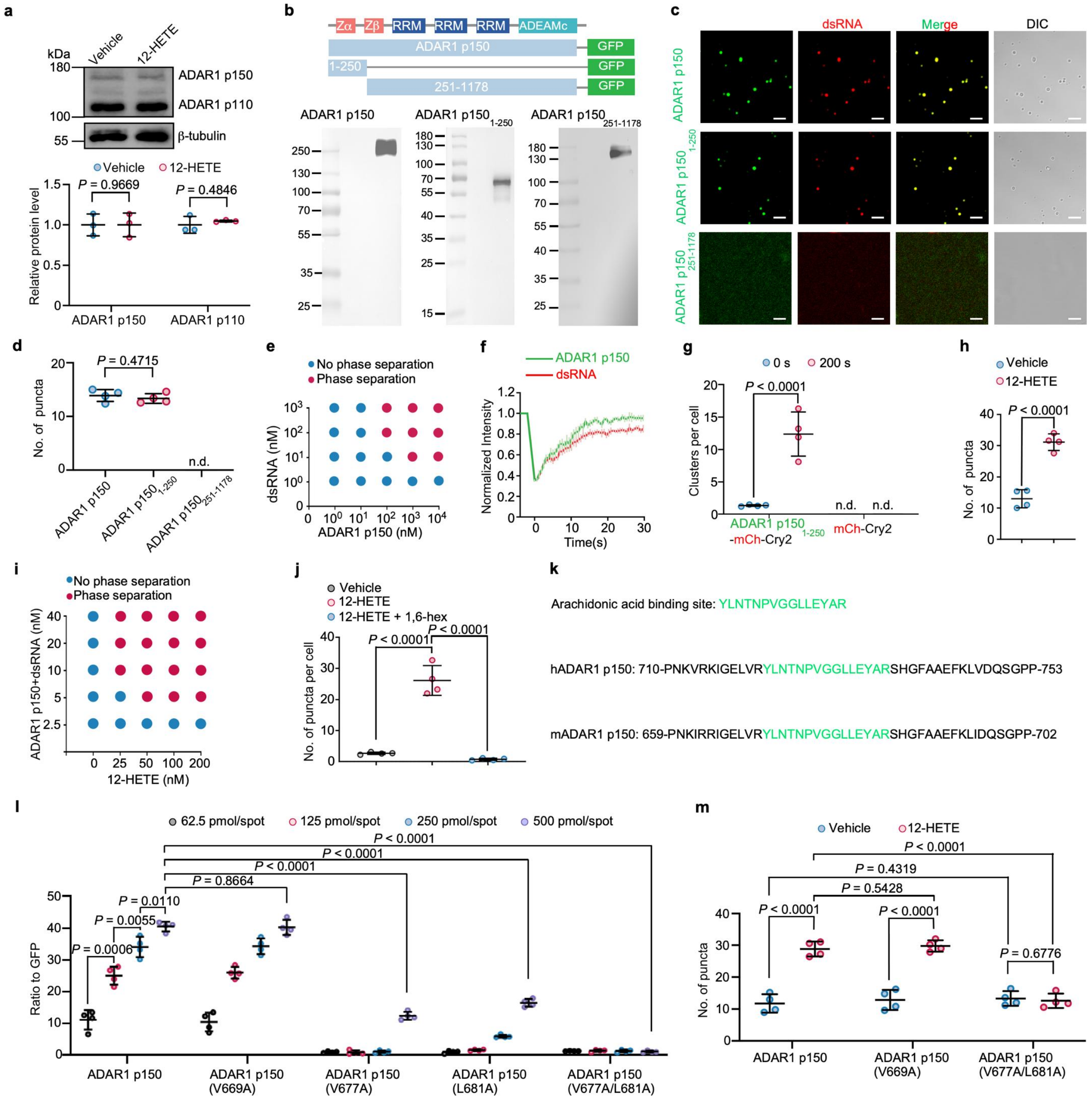

Supplementary Fig. 11 | 12-HETE enhances ADAR1 p150-dsRNA liquid-liquid phase separation. a, Representative immunoblots (top) and quantitation (bottom) of ADAR1 p150 and ADAR1 p110 levels in EO771 cells with indicated treatment. **b**, Schematic of domain architecture of His6-MBP-ADAR1 p150-GFP, His6-ADAR1 p150$_{1\text{-}250}$-GFP and His6-ADAR1 p150$_{251\text{-}1178}$-GFP constructs. The purified recombinant proteins were analyzed by western blotting. **c**, **d**, Representative fluorescence and differential interference contrast (DIC) images (c) and quantitation (d) of droplets formed by Cy5-dsRNA and ADAR1-GFP proteins from indicated constructs. Scale bars, 5 μm. Each dot represents data pooled from 5 fields of each independent experiment. **e**, Phase separation diagram of ADAR1 p150 and dsRNA at indicated concentrations. Blue dots: no phase separation; Red dots: phase separation. **f**, Changes in the fluorescence intensity of ADAR1 p150-dsRNA droplets in Fig. 6g were plotted over time after photobleaching. **g**, The quantitation of Fig. 6h. Each dot represents data pooled from 10 cells of

each independent experiment. **h**, The quantitation of Fig. 6j. Each dot represents data pooled from 5 fields of each independent experiment. **i**, Phase separation diagram of ADAR1 p150 and dsRNA with indicated 12-HETE concentrations. Blue dots: no phase separation; Red dots: phase separation. **j**, The quantitation of Fig. 6k. Each dot represents data pooled from 10 cells of each independent experiment. **k**, The previously reported arachidonic acid binding site (YLNTNPVGGLLEYAR) is shown for comparison with sequence of human ADAR1 p150-dsRBD3 (Pro710-Pro753) and mouse ADAR1 p150-dsRBD3 (Pro659-Pro702). **l**, The quantitation of Fig. 6n. **m**, The quantitation of Fig. 6o. Each dot represents data pooled from 5 fields of each independent experiment.

n = 3 independent experiments (a); n = 4 independent experiments (d, g, h, j, l, m). n = 6 independent experiments (f); Mean ± SD (a, d, f-h, j, l, m). *P* values were calculated by two-tailed unpaired t-test (a, d, g, h, l, m) and one-way ANOVA with Tukey's multiple comparisons test (j). n.d., not detected.

Supplementary Fig. 12

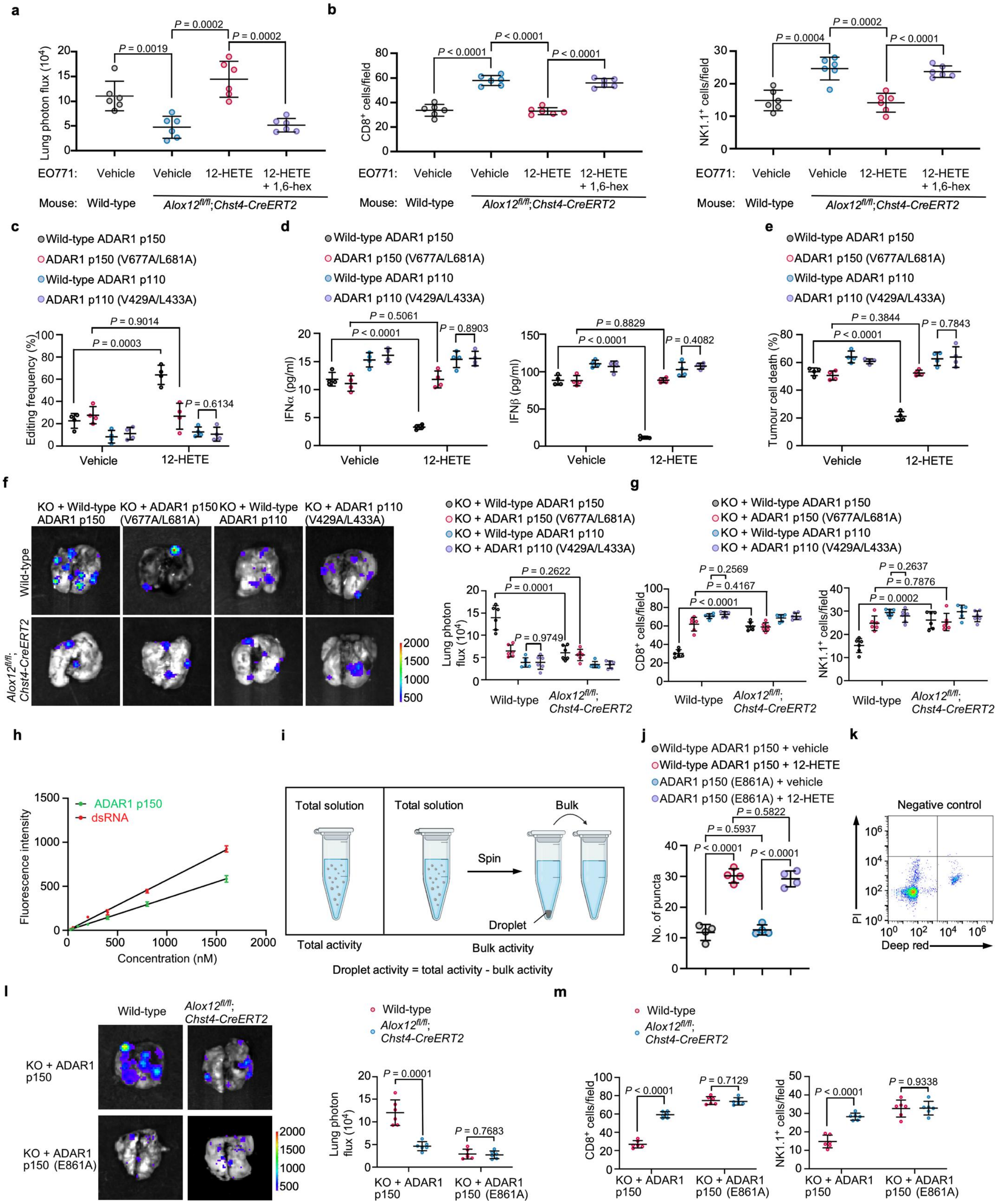

Supplementary Fig. 12 | ADAR1 p150-dsRNA liquid-liquid phase separation induced by 12-HETE promotes A-to-I RNA editing. a, The quantitation of Fig. 7d. **b**, The quantitation of Fig. 7e. Each dot represents data pooled from 5 distinct areas from a slice and 3 slices per mouse. **c**, Indicated ADAR1 protein variants were mixed with dsRNA in the presence or absence of 12-HETE for 30 minutes. The edited dsRNA (fraction of substrate dsRNA molecules that were

modified at least once) was quantified. **d**, IFNα and IFNβ levels in the media of *Adar1*-konckout EO771 cells transduced with indicated ADAR1 variants and treated with or without 12-HETE. **e**, The quantitation for $CD8^+$ T cell-mediated cytotoxicity to *Adar1*-konckout EO771 cells transduced with indicated ADAR1 variants and treated with or without 12-HETE. **f**, Representative bio-luminescent images (left) and quantitation (right) of lungs harvested from mice inoculated with indicated luciferase-expressing EO771 cells via fat pad injection. **g**, The quantitation of immunohistochemistry staining for CD8α and NK1.1 in the lung metastases of mice inoculated with indicated cells with indicated treatment. Each dot represents data pooled from 5 distinct areas from a slice and 3 slices per mouse. **h**, The standard curves generated from the fluorescence intensities and concentrations of ADAR1 p150-GFP and Cy5-dsRNA. **i**, Schematic to measure editing rates in the ADAR1 p150-dsRNA droplet phase. The total editing rate is measured by simply mixing all components. Bulk rate is measured by centrifuging the total mixture sediment droplets and transferring the supernatant to a new tube. The difference between total and bulk rate yields the droplet rate. **j**, The quantitation of Fig. 7i. **k**, Unstimulated T cells were used as a negative control of Fig. 7k. **l**, Representative bio-luminescent images (left) and quantitation (right) of lungs harvested from mice inoculated with indicated luciferase-expressing EO771 cells via fat pad injection. **m**, The quantitation of immunohistochemistry staining for CD8α and NK1.1 in the lung metastases of mice inoculated with indicated cells with indicated treatment. Each dot represents data pooled from 5 distinct areas from a slice and 3 slices per mouse.
n = 6 mice per group (a, b, f, g, l, m); n = 4 independent experiments (c-e, h, j). Mean ± SD (a-h, j, l, m). *P* values were calculated by one-way ANOVA with Tukey's multiple comparisons test (a, b, j) and two-tailed unpaired *t*-test (c-g, l, m).

Supplementary Fig. 13

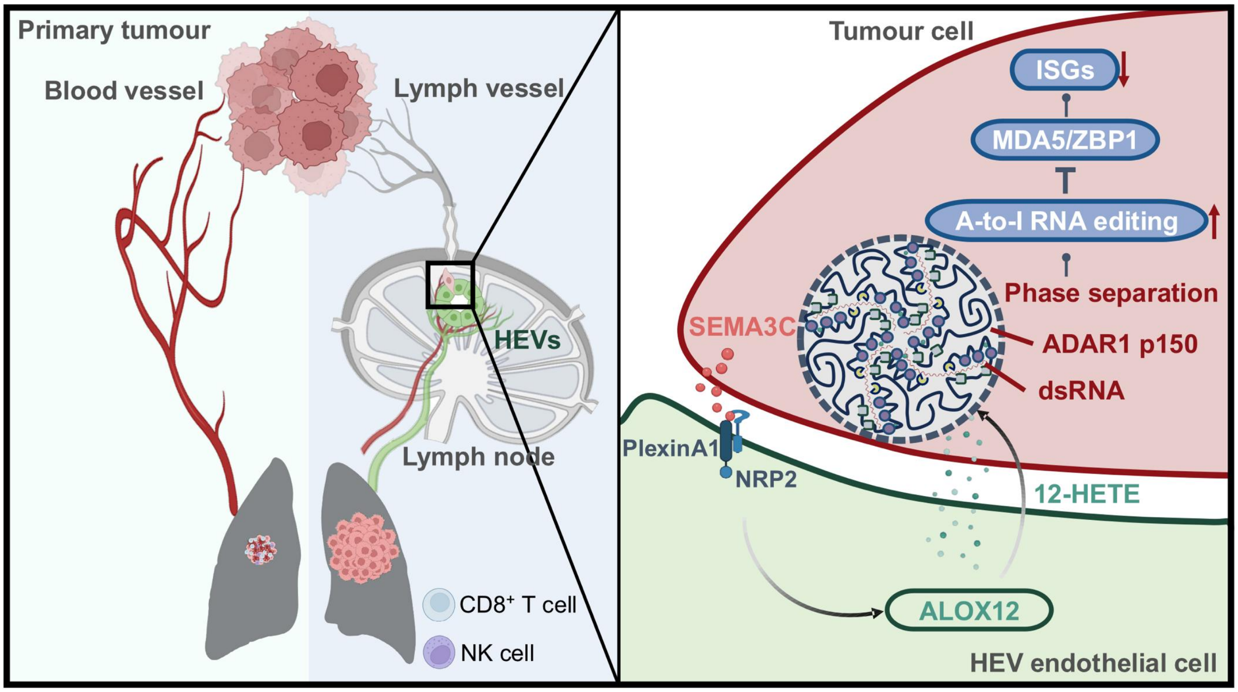

Supplementary Fig. 13 | Schematic highlighting the primary finding of this study. Tumour-derived SEMA3C induces ALOX12 expression in HEVs, leading to the production of 12-HETE. 12-HETE promotes ADAR1 p150–dsRNA phase separation in tumour cells, enhancing A-to-I RNA editing and thereby reducing dsRNA-mediated immune response, which facilitates metastatic colonization.

Supplementary Table 1. Association between HEV involvement and clinical features in the SLN-positive cohort from the Sun Yat-Sen Memorial Hospital (n = 559).

Characteristic	Total		HEVI		No HEVI		
	No.	%	No.	%	No.	%	***P***
Total	559	100	208	100	351	100	
Age group							.788
≤ 45	214	38	78	38	136	39	
> 45	345	62	130	62	215	61	
Menopausal status							.432
premenopause	289	52	103	50	186	53	
menopause	270	48	105	50	165	47	
Tumour size							.382
≤ 2 cm	291	52	103	50	188	54	
> 2 cm	268	48	105	50	163	46	
Grade							.790
1/2	327	58	120	58	207	59	
3	232	42	88	42	144	41	
Molecular subtypes							.674
HR^+HER2^-	381	68	145	70	236	67	
HR^+HER2^+	80	14	27	13	53	15	
HR^-HER2^-	66	12	22	11	44	13	
HR^-HER2^+	32	6	14	6	18	5	
No. of positive SLNs							.103
1-2	493	88	177	85	316	90	
⩾3	66	12	31	15	35	10	
Area of SLN metastases							.727
≤ 5.7 mm^2	280	50	102	49	178	51	
> 5.7 mm^2	279	50	106	51	173	49	
LVI							.541
Absent	273	49	98	47	175	50	
Present	286	51	110	53	176	50	
KI67							
Low	174	31	61	30	113	32	.509
High	385	69	147	70	238	68	

P values were calculated by two-sided Chi-square test or Fisher's exact test where appropriate. Abbreviations: HEVI, high endothelial venule involvement; SLN, sentinel lymph node; HR, hormone receptor; HER2, human epidermal growth factor receptor; LVI, lymphovascular invasion.

Supplementary Table 2. Cox regression analysis of DFS in the SLN-positive cohort from the Sun Yat-Sen Memorial Hospital (n = 559).

**Characteristic**	**DFS in SLN (+) cohort**			
	Univariate	Multivariate		
	P	HR	95% CI	*P*
Age (> 45)	0.802			
Premenopausis	0.498			
Tumour size (> 2cm)	0.002	1.543	1.139-2.089	0.005*
Grade (III)	0.002	1.281	0.915-1.795	0.149
ER (positive)	0.039	0.769	0.541-1.093	0.143
PR (positive)	0.477			
HER2 (positive)	0.525			
LVI	0.003	1.467	1.074-2.002	0.016*
KI67 (high)	0.087			
Lymph node (N3)	<0.001	2.431	1.690-3.497	<0.001*
HEV involvement	<0.001	2.008	1.486-2.713	<0.001*

Hazard ratios (HRs) and corresponding *P* values were calculated using the Wald test. Abbreviations: SLN, sentinel lymph node; ER, estrogen receptor; PR, progesterone receptor; HER2, human epidermal growth factor receptor; HEV, high endothelial venule; LVI, lymphovascular invasion; CI, confidence interval.

Supplementary Table 3. Cox regression analysis of OS in the SLN-positive cohort from the Sun Yat-Sen Memorial Hospital (n = 559).

**Characteristic**	**OS in SLN (+) cohort**			
	Univariate	Multivariate		
	P	HR	95% CI	*P*
Age (> 45)	0.440			
Premenopausis	0.441			
Tumour size (> 2cm)	<0.001	2.279	1.394-3.728	0.001*
Grade (III)	0.075			
ER (positive)	0.229			
PR (positive)	0.320			
HER2 (positive)	0.388			
LVI	0.003	1.936	1.171-3.202	0.010*
KI67 (high)	0.728			
Lymph node (N3)	<0.001	2.211	1.250-3.911	0.006*
HEV involvement	0.002	1.948	1.220-3.109	0.005*

HRs and corresponding *P* values were calculated using the Wald test. Abbreviations: SLN, sentinel lymph node; ER, estrogen receptor; PR, progesterone receptor; HER2, human epidermal growth factor receptor; HEV, high endothelial venule; LVI, lymphovascular invasion; CI, confidence interval.

Supplementary Table 4. Mutation status of EO771, Py230 and B16F10 cell lines.

**Gene**	***Trp53***	***Pten***	***Erbb2***	***Kras***	***Pik3ca***
EO771-Parental	MUT	WT	WT	MUT	WT
EO771-BVM3	MUT	WT	WT	MUT	WT
EO771-HEVM3	MUT	WT	WT	MUT	WT
Py230-Parental	WT	WT	WT	WT	WT
Py230-BVM3	WT	WT	WT	WT	WT
Py230-HEVM3	WT	WT	WT	WT	WT

**Gene**	***Trp53***	***Pten***	***Braf***	***Kit***	***Pik3ca***
B16F10-Parental	MUT	MUT	MUT	WT	WT
B16F10-BVM3	MUT	MUT	MUT	WT	WT
B16F10-HEVM3	MUT	MUT	MUT	WT	WT

Mutation was identified by whole exome sequencing of the cell lines. Gene mutation status is given by WT (wild-type) or MUT (mutant).
