# Supplementary material for: Tumour-associated high endothelial venules drive portal-specific immune evasion in lymph nodes via ALOX12
Source: Nat Commun. 2026 May 11;17:6364. doi: 10.1038/s41467-026-72412-w (PMC13376640; doi:10.1038/s41467-026-72412-w)
Supplement: Supplementary file 2 — Description of Additional Supplementary Files [file 41467_2026_72412_MOESM2_ESM.pdf]

Description of Additional Supplementary Files

Supplementary Dataset 1: Clinical features of the ALN-positive cohort from the Sun Yat-Sen Memorial Hospital (n = 457).

Supplementary Dataset 2: Genes enriched in HECs and upregulated in tumour-bearing lymph nodes.

Supplementary Dataset 3: Quantitative proteomic analysis of biotinylated proteins in HECs from metastatic lymph nodes using TurboID proximity labeling.

Supplementary Dataset 4: A list of oligonucleotides used in the study.

Supplementary Movie 1: Intravital multiphoton microscopic imaging of GFPexpressing EO771 cells in the lymph nodes of Chst4-CreERT2-tdTomato mice.
