# Supplementary material for: Tumour-associated high endothelial venules drive portal-specific immune evasion in lymph nodes via ALOX12
Source: Nat Commun. 2026 May 11;17:6364. doi: 10.1038/s41467-026-72412-w (PMC13376640; doi:10.1038/s41467-026-72412-w)
Supplement: Supplementary file 8 — Reporting Summary [file 41467_2026_72412_MOESM8_ESM.pdf]

nature portfolio

Corresponding author(s): Shicheng Su

Last updated by author(s): Feb 25, 2026

Reporting Summary

Nature Portfolio wishes to improve the reproducibility of the work that we publish. This form provides structure for consistency and transparency in reporting. For further information on Nature Portfolio policies, see our Editorial Policies and the Editorial Policy Checklist.

Statistics

For all statistical analyses, confirm that the following items are present in the figure legend, table legend, main text, or Methods section.

n/a	Confirmed	
☐	☒	The exact sample size (*n*) for each experimental group/condition, given as a discrete number and unit of measurement
☐	☒	A statement on whether measurements were taken from distinct samples or whether the same sample was measured repeatedly
☐	☒	The statistical test(s) used AND whether they are one- or two-sided<br>*Only common tests should be described solely by name; describe more complex techniques in the Methods section.*
☒	☐	A description of all covariates tested
☐	☒	A description of any assumptions or corrections, such as tests of normality and adjustment for multiple comparisons
☐	☒	A full description of the statistical parameters including central tendency (e.g. means) or other basic estimates (e.g. regression coefficient) AND variation (e.g. standard deviation) or associated estimates of uncertainty (e.g. confidence intervals)
☐	☒	For null hypothesis testing, the test statistic (e.g. *F*, *t*, *r*) with confidence intervals, effect sizes, degrees of freedom and *P* value noted<br>*Give P values as exact values whenever suitable.*
☒	☐	For Bayesian analysis, information on the choice of priors and Markov chain Monte Carlo settings
☒	☐	For hierarchical and complex designs, identification of the appropriate level for tests and full reporting of outcomes
☐	☒	Estimates of effect sizes (e.g. Cohen's *d*, Pearson's *r*), indicating how they were calculated

Our web collection on statistics for biologists contains articles on many of the points above.

Software and code

Policy information about availability of computer code

Data collection	Whole-slide images were acquired by Axio Scan.Z1 (Zeiss). Intravital multiphoton microscopy images were acquired by Olympus laser-scanning microscope (FVMPE-RS). FACS data was collected from a flow cytometer (BD Biosciences) and CytoFLEX flow cytometer (Beckman Coulter). Tumor metastases were monitored by using IVIS Spectrum Xenogen (Caliper Life Sciences). H&E and IHC data were imaged by Olympus BX63 microscopy. IF and DIC images were imaged by laser scanning confocal microscopy (LSM800/LSM880, Zeiss). RNA-seq data was collected on Illumina Novaseq 6000 platform and MGISEQ-2000 System. MS data were acquired by an Ultra Performance Liquid Chromatography (Shim-pack UFLC SHIMADZU CBM30A system, SHIMADZU) coupled to Tandem mass spectrometry (QTRAP, SCIEX).Biotinylated proteins were analyzed using a Vanquish Neo UHPLC system coupled to a Orbitrap Astral mass spectrometer (Thermo Fisher Scientific). Quantitative real-time PCR was run on LightCycler 480 (Roche). Immunoblotting images were collected using a gel imaging system (Syngene G:BOX). Fluorescence reporter signals of thermal shift assay were recorded on a 7500 Real-Time PCR System (Applied Biosystems).

Data analysis

HEV and tumor lesion locations were labeled using QuPath software (v0.2.3). Deep neural networks to detect the HEV involvement locations was developed using Pytorch platforms (v1.11.0). GraphPad Prism (v10.0.0) was used for data analysis. FACS data were analyzed with FlowJo (v10.0.7). Bioluminescence imaging data was analysed using Living Image software (v.2.50). IHC images were analyzed using QuPath (v0.2.3). IF images were analyzed using CellProfiler (v4.2.4). RNA-seq sequence processing and statistics was performed using STAR (v2.6.0c), featureCounts (v1.6.2), DESeq2 (v1.34.0) and RepeatMasker (v.4.09). Single-cell sequence data was analyzed using CellRanger (v6.0.2). RNA-seq and single-cell data visualization was performed in R (v4.1.1) with ClusterProfiler (v4.0.5), pheatmap (v1.0.12), Seurat (v4.1.1), DoubletFinder (v2.0.3) and CellChat (v1.5.0). Blot intensity was measured with ImageJ (v1.52a). Molecular dynamics simulations were performed using GROMACS-2020 suite and visualized using PyMOL (1.7.0.0). Custom code related to the deep learning models is available at https://github.com/xiaqd/HEVI-detection.

For manuscripts utilizing custom algorithms or software that are central to the research but not yet described in published literature, software must be made available to editors and reviewers. We strongly encourage code deposition in a community repository (e.g. GitHub). See the Nature Portfolio guidelines for submitting code & software for further information.

Data

Policy information about availability of data

All manuscripts must include a data availability statement. This statement should provide the following information, where applicable:

- Accession codes, unique identifiers, or web links for publicly available datasets
- A description of any restrictions on data availability
- For clinical datasets or third party data, please ensure that the statement adheres to our policy

RNA-seq and scRNA-seq datasets generated in this study have been deposited in GEO (accession GSE217454), and whole-exome sequencing data are available in SRA (accession PRJNA1024076). The mass spectrometry proteomics data have been deposited to the ProteomeXchange Consortium (https://proteomecentral.proteomexchange.org) via the iProX partner repository with the dataset identifier PXD074282. All experimental materials and protocols are detailed in the Methods, with relevant references cited. Restrictions apply to the availability of the medical training/validation data in accordance with hospital requirements governing human subject privacy protection, and so are not publicly available. Some of the data can be provided by the authors upon reasonable request, subject to approval from Sun Yat-Sen Memorial Hospital. The raw numbers for charts and graphs are available in the Source Data file. Source Data are provided with this paper.

Research involving human participants, their data, or biological material

Policy information about studies with human participants or human data. See also policy information about sex, gender (identity/presentation), and sexual orientation and race, ethnicity and racism.

Reporting on sex and gender

Only female breast cancer patients were included in this study.

Reporting on race, ethnicity, or other socially relevant groupings

From 2008 to 2015, the records of all patients with primary invasive breast cancer and at least one positive sentinel lymph node (SLN) obtained by SLN biopsy or at least one positive axillary lymph node (ALN) obtained by ALN dissection without SLN biopsy were retrospectively reviewed at the Sun Yat-Sen Memorial Hospital, Sun Yat-Sen University (Guangzhou, China). From 2010 to 2015, the record of all patients with primary invasive breast cancer and at least one positive SLN were retrospectively reviewed at the Affiliated Hospital of Guizhou Medical University (Guiyang, China) and the First Affiliated Hospital of Zhengzhou University (Zhengzhou, China). Patients with incomplete clinical information, unavailable pathological slides, another concurrent cancer, or recurrence/metastasis at diagnosis were excluded. Group characteristics are presented in Extended Data Table 1 and 2. Paraffin-embedded lymph node metastases were used for H&E and immunofluorecent staining.

Population characteristics

See above

Recruitment

All samples were collected from patients with informed consent.

Ethics oversight

All related procedures were approved by the internal review and ethics boards of Sun Yat-Sen Memorial Hospital, the Affiliated Hospital of Guizhou Medical University and the First Affiliated Hospital of Zhengzhou University.

Note that full information on the approval of the study protocol must also be provided in the manuscript.

Field-specific reporting

Please select the one below that is the best fit for your research. If you are not sure, read the appropriate sections before making your selection.

☒ Life sciences ☐ Behavioural & social sciences ☐ Ecological, evolutionary & environmental sciences

For a reference copy of the document with all sections, see nature.com/documents/nr-reporting-summary-flat.pdf

Life sciences study design

All studies must disclose on these points even when the disclosure is negative.

Sample size

No statistical methods were used to predetermine sample sizes but our sample sizes are similar to those reported in previous publications. Sample size for every experiment has been described in the manuscript and the data shown are representative of at least three independent

experiments. Animal experiments used at least six mice per group per experiments.

Data exclusions	No data were excluded (unless otherwise specified).
Replication	As reported in the figure legends, experiments were performed at least three times, the findings were reliably reproduced.
Randomization	For all in vivo experiments, animals were randomly assigned into experimental groups and age-matched. Whole-slide images of metastatic LNs were splitted randomly into the training, validation and test set at the patient level to build deep learning model.
Blinding	HEV and tumor lesion locations were labeled manually by two pathology-trained operators blinded to the patients' clinical background. Investigators were not blinded to mouse genotypes during experiments. H&E, immunohistochemistry, immunofluorescence and immunoblotting images were scored by the person blinded to the group information. Tumor measurements were performed by the person blinded to the treatment conditions.

Reporting for specific materials, systems and methods

We require information from authors about some types of materials, experimental systems and methods used in many studies. Here, indicate whether each material, system or method listed is relevant to your study. If you are not sure if a list item applies to your research, read the appropriate section before selecting a response.

Materials & experimental systems

n/a	Involved in the study
☐	☒ Antibodies
☐	☒ Eukaryotic cell lines
☒	☐ Palaeontology and archaeology
☐	☒ Animals and other organisms
☒	☐ Clinical data
☒	☐ Dual use research of concern
☒	☐ Plants

Methods

n/a	Involved in the study
☒	☐ ChIP-seq
☐	☒ Flow cytometry
☒	☐ MRI-based neuroimaging

Antibodies

Antibodies used

All the antibodies are from commercial sources and have been validated by the vendors and their validation data are available on the manufacturer's website.
For immunohistochemistry and immunofluorescence: anti-NK1.1 (1:100, Thermo Fisher Scientific, #MA1-70100), anti-CD8α (1:2000, Abcam, #ab217344),anti-pan cytokeratin (1:100, Sigma Aldrich, #c2562), anti-PNAd (1:50, Biolegend, #120802), anti-CD31 (1:2000, Abcam, #ab182981), anti-ALOX12 (1:150, Novus Biologicals, #NBP2-46512), anti-CD11c (1:100,Cell Signaling Technology,#97585), anti-CK18 (1:100, Abcam, #ab219271), anti-SEMA3C (1:100, Abcam, #ab214309), anti-CX40 (1:50, Thermo Fisher Scientific, #36-4900) anti-EMCN (1:200, Thermo Fisher Scientific, #PA5-47648), anti-MDA5 (1:100, Abcam, #ab79055), anti-ZBP1 (1:100, Thermo Fisher Scientific, #PA5-20455), anti-J2 (1:100, Sigma Aldrich, #MABE1134)
For flow cytometry: anti-mouse CD45 (1:25, Thermo Fisher Scientific, #MCD4528), anti-mouse/human CD31 (1:25, Thermo Fisher Scientific, #17-0311-82), anti-mouse/human PNAd (1:25, Thermo Fisher Scientific, #53-6036-82),anti-human CD45 (1:25, Thermo Fisher Scientific, #MHCD4528), anti-human CD31 (1:25, Thermo Fisher Scientific, #17-0319-42), anti-mouse CD45 (1:25, BD Biosciences, #563891), anti-mouse CD90.2 (1:25, BD Biosciences, #553003), anti-mouse CD19 (1:25, BD Biosciences, #557398), anti-mouse Ly6C (1:25, BD Biosciences, #560596), anti-mouse CD24 (1:25, Thermo Fisher Scientific, #25-0242-82), anti-mouse CD11b (1:25, Biolegend, #101228), anti-mouse CD11c (1:25, Biolegend, #117308), anti-mouse MHC? (1:25, Biolegend, #116418), anti-mouse CD103 (1:25, BD Biosciences, #562771), anti-mouse CD40 (1:25, BD Biosciences, #745218), anti-mouse CD80 (1:25, BD Biosciences #740698), anti-mouse CD3 (1:25, Biolegend, #100205), anti-mouse CD8a (1:25, Biolegend, #100711), anti-human/mouse Granzyme B (1:25, Biolegend, #515403), APC anti-mouse CD4 (1:25, Biolegend, #100411), Alexa Fluor 488 anti- human/mouse FOXP3 (1:25, Biolegend, #320011).
For immunoblotting: rabbit anti-human/mouse SEMA3C (1:1000, Thermo Fisher Scientific, #PA5-24997), rabbit anti-human/mouse NRG1 (1:1000, Abcam, #ab217805), rabbit anti-human/mouse TFF2 (1:1000, Thermo Fisher Scientific, #PA5-75670), rabbit anti-human/mouse APOC4 (1:1000, Thermo Fisher Scientific, #PA5-75699), rabbit anti-human/mouse RBP4 (1:1000, Abcam, #ab109193),rabbit anti-human/mouse MDA5 (1:1000, Abcam, #315242), rabbit anti-human/mouse ZBP1 (1:2000, Thermo Fisher Scientific, #PA5-20455), anti-GADPH (1:10000, Proteintech, #HRP-60004), mouse anti-human/mouse ADAR1 (1:1000, Santa Cruz Biotechnology, #sc-73408), rabbit anti-human/mouse SOCS1 (1:500, Thermo Fisher Scientific, #38-5200), rabbit anti-human/mouse PTPN2 (1:500, Thermo Fisher Scientific, #PA5-78138) and anti-β-tubulin (1:5000, Proteintech, #10068-1-AP)

Validation

anti-NK1.1 (1:100, Thermo Fisher Scientific, #MA1-70100): https://www.thermofisher.cn/cn/zh/antibody/product/NK1-1-Antibody-clone-PK136-Monoclonal/MA1-70100;
anti-CD8α (1:2000, Abcam, #ab217344): https://www.abcam.com/cd8-alpha-antibody-epr21769-ab217344.html;
anti-pan cytokeratin (1:100, Sigma Aldrich, #c2562): https://www.sigmaaldrich.com/TW/en/product/sigma/c2562;
anti-PNAd (1:50, Biolegend, #120802): https://www.biolegend.com/en-us/punchout/search-results/purified-anti-mouse-human-pnad-antibody-2975?GroupID=BLG4607;
anti-CD31 (1:2000, Abcam, #ab182981): https://www.abcam.com/cd31-antibody-epr17259-ab182981.html;
anti-ALOX12 (1:150, Novus Biologicals, #NBP2-46512): https://www.novusbio.com/products/12-lipoxygenase-antibody-oti1c3_nbp2-46512;
anti-CD11c (1:100,Cell Signaling Technology,#97585): https://www.cellsignal.com/products/primary-antibodies/cd11c-d1v9y-rabbit-

mab/97585;
anti-CK18 (1:100, Abcam, #ab219271): https://www.abcam.com/en-us/products/primary-antibodies/cytokeratin-18-antibody-ab219271?srsltid=AfmBOopkgMlPvVCGFcKP-2h5b4ST87Mf546HdVHJTCsce7nie661VzGY;
anti- SEMA3C (1:100, Abcam, #ab214309)https://www.abcam.com/en-us/products/primary-antibodies/semaphorin-3c-antibody-ab214309?srsltid=AfmBOorEPrpIOETIDi2qBq2NVQSdMv5bUEuVrssBbRCilf92cwb4yVQL;
anti-CX40 (1:50, Thermo Fisher Scientific, #36-4900):https://www.thermofisher.cn/cn/zh/antibody/product/Connexin-40-Antibody-Polyclonal/36-4900;
anti-EMCN (1:200, Thermo Fisher Scientific, #PA5-47648):https://www.thermofisher.cn/cn/zh/antibody/product/Endomucin-Antibody-Polyclonal/PA5-47648;
rabbit anti-human/mouse MDA5 (1:100, Abcam, #ab79055): https://www.abcam.com/en-us/products/primary-antibodies/mda5-antibody-ab79055?srsltid=AfmBOoqmVvwKbalRu27-x1cgo7xgPPN0YHcZs0Ln2L5TeW3xNRp7z3SP;
rabbit anti-human/mouse ZBP1 (1:100, Thermo Fisher Scientific, #PA5-20455): https://www.thermofisher.cn/cn/zh/antibody/product/ZBP1-Antibody-Polyclonal/PA5-20455;
mouse anti-J2 (1:100, Sigma Aldrich, #MABE1134): https://www.sigmaaldrich.com/HK/en/product/mm/mabe1134?srsltid=AfmBOoqQJo2u_AwnhHkA4UmzJmUbC8d3yg8ts3ePOopjMw8_9Tl9xEZJ;
anti-mouse CD45 (1:25, Thermo Fisher Scientific, #MCD4528): https://www.thermofisher.cn/cn/zh/antibody/product/CD45-Antibody-clone-30-F11-Monoclonal/MCD4528;
anti-mouse/human CD31 (1:25, Thermo Fisher Scientific, #17-0311-82): https://www.thermofisher.cn/cn/zh/antibody/product/CD31-PECAM-1-Antibody-clone-390-Monoclonal/17-0311-82;
anti-mouse/human PNAd (1:25, Thermo Fisher Scientific, #53-6036-82): https://www.thermofisher.cn/cn/zh/antibody/product/High-Endothelial-Venule-Marker-Antibody-clone-MECA-79-Monoclonal/53-6036-82;
anti-human CD45 (1:25, Thermo Fisher Scientific, #MHCD4528): https://www.thermofisher.cn/cn/zh/antibody/product/CD45-Antibody-clone-HI30-Monoclonal/MHCD4528;
anti-human CD31 (1:25, Thermo Fisher Scientific, #17-0319-42): https://www.thermofisher.cn/cn/zh/antibody/product/CD31-PECAM-1-Antibody-clone-WM-59-WM59-Monoclonal/17-0319-42;
anti-mouse CD45 (1:25, BD Biosciences, #563891): https://www.bdbiosciences.com/en-eu/products/reagents/flow-cytometry-reagents/research-reagents/single-color-antibodies-ruo/bv510-rat-anti-mouse-cd45.563891;
anti-mouse CD90.2 (1:25, BD Biosciences, #553003): https://www.bdbiosciences.com/en-eu/products/reagents/flow-cytometry-reagents/research-reagents/single-color-antibodies-ruo/fitc-rat-anti-mouse-cd90-2.553003;
anti-mouse CD19 (1:25, BD Biosciences, #557398): https://www.bdbiosciences.com/en-be/products/reagents/flow-cytometry-reagents/research-reagents/single-color-antibodies-ruo/FITC-Rat-Anti-Mouse-CD19.557398;
anti-mouse Ly6C (1:25, BD Biosciences, #560596): https://www.bdbiosciences.com/en-eu/products/reagents/flow-cytometry-reagents/research-reagents/single-color-antibodies-ruo/apc-cy-7-rat-anti-mouse-ly-6c.560596;
anti-mouse CD24 (1:25, Thermo Fisher Scientific, #25-0242-82): https://www.thermofisher.cn/cn/zh/antibody/product/CD24-Antibody-clone-M1-69-Monoclonal/25-0242-82;
anti-mouse CD11b (1:25, Biolegend, #101228): https://www.biolegend.com/en-gb/search-results/percp-cyanine5-5-anti-mouse-human-cd11b-antibody-4257?GroupID=BLG10552;
anti-mouse CD11c (1:25, Biolegend, #117308): https://www.biolegend.com/ja-jp/products/pe-anti-mouse-cd11c-antibody-1816?GroupID=BLG11937;
anti-mouse MHC? (1:25, Biolegend, #116418): https://www.biolegend.com/ja-jp/products/apc-anti-mouse-i-ab-antibody-6597;
anti-mouse CD103 (1:25, BD Biosciences, #562771): https://www.bdbiosciences.com/ja-jp/products/reagents/flow-cytometry-reagents/research-reagents/single-color-antibodies-ruo/bv421-rat-anti-mouse-cd103.562771;
anti-mouse CD40 (1:25, BD Biosciences, #745218): https://www.bdbiosciences.com/en-eu/products/reagents/flow-cytometry-reagents/research-reagents/single-color-antibodies-ruo/bv605-rat-anti-mouse-cd40.745218;
anti-mouse CD80 (1:25, BD Biosciences #740698): https://www.bdbiosciences.com/en-eu/products/reagents/flow-cytometry-reagents/research-reagents/single-color-antibodies-ruo/bv711-hamster-anti-mouse-cd80.740698;
anti-mouse CD3 (1:25, Biolegend, #100205): https://www.biolegend.com/de-at/products/pe-anti-mouse-cd3-antibody-47;
anti-mouse CD8a (1:25, Biolegend, #100711): https://www.biolegend.com/fr-lu/products/apc-anti-mouse-cd8a-antibody-150?GroupID=BLG6765;
anti-human/mouse Granzyme B (1:25, Biolegend, #515403): https://www.biolegend.com/ja-jp/products/fitc-anti-human-mouse-granzyme-b-antibody-6066?GroupID=BLG15670;
APC anti-mouse CD4 (1:25, Biolegend, #100411): https://www.biolegend.com/en-gb/lyophilized-control-cells/apc-anti-mouse-cd4-antibody-245;
Alexa Fluor 488 anti- human/mouse FOXP3 (1:25, Biolegend, #320011): https://www.biolegend.com/en-gb/products/alexa-fluor-488-anti-mouse-rat-human-foxp3-antibody-2891;
rabbit anti-human/mouse SEMA3C (1:1000, Thermo Fisher Scientific, #PA5-24997):https://www.thermofisher.cn/cn/zh/antibody/product/SEMA3C-Antibody-Polyclonal/PA5-24997?adobe_mc=MCMID%7C17548599034491578672571114937068294743%7CMCAID%3D34569058A82208A3-40000A86A126896F%7CMCORGID%3D5B135A0C5370E6B40A490D44%40AdobeOrg%7CTS=1614293705;
rabbit anti-human/mouse NRG1 (1:1000, Abcam, #ab217805):https://www.abcam.com/en-us/products/primary-antibodies/nrg1-antibody-ab217805?srsltid=AfmBOopD-8d5w2ZOxGAPjtyEyIS6HmrOajSmT6YInXs_bYZNkqc9Mt3I;
rabbit anti-human/mouse TFF2 (1:1000, Thermo Fisher Scientific, #PA5-75670):https://www.thermofisher.cn/cn/zh/antibody/product/TFF2-Antibody-Polyclonal/PA5-75670?adobe_mc=MCMID%7C17548599034491578672571114937068294743%7CMCAID%3D34569058A82208A3-40000A86A126896F%7CMCORGID%3D5B135A0C5370E6B40A490D44%40AdobeOrg%7CTS=1614293705;
rabbit anti-human/mouse APOC4 (1:1000, Thermo Fisher Scientific, #PA5-75699):https://www.thermofisher.cn/cn/zh/antibody/product/APOC4-Antibody-Polyclonal/PA5-75699?adobe_mc=MCMID%7C17548599034491578672571114937068294743%7CMCAID%3D34569058A82208A3-40000A86A126896F%7CMCORGID%3D5B135A0C5370E6B40A490D44%40AdobeOrg%7CTS=1614293705;
rabbit anti-human/mouse RBP4 (1:1000, Abcam, #ab109193):https://www.abcam.com/en-us/products/primary-antibodies/rbp4-antibody-ep3657-ab109193?srsltid=AfmBOorRlMrhNmTfpGV79nmfW0Y4Gbr12fSY2E_rbCzn4j88QonIWIx2;
anti-GADPH (1:10000, Proteintech, #HRP-60004):https://www.ptglab.com/products/GAPDH-Antibody-HRP-60004.htm?srsltid=AfmBOoqgG7cMlug3hP1UMcuwzh-qjOXRrbBr3YH34o781eBrIVynguy1;
mouse anti-human/mouse ADAR1 (1:1000, Santa Cruz Biotechnology, #sc-73408):https://www.scbt.com/p/adar1-antibody-15-8-6?srsltid=AfmBOoqTqnlNLTxmlvcENy5uBUmpP-Msr6Trk6LDbVEB40TqjF2Lvavh;
rabbit anti-human/mouse SOCS1 (1:500, Thermo Fisher Scientific, #38-5200):https://www.thermofisher.cn/cn/zh/antibody/product/SOCS1-Antibody-Polyclonal/38-5200;
rabbit anti-human/mouse PTPN2 (1:500, Thermo Fisher Scientific, #PA5-78138):https://www.thermofisher.cn/cn/zh/antibody/

product/PTPN2-Antibody-Polyclonal/PA5-78138;
anti-β-tubulin (1:5000, Proteintech, #10068-1-AP):https://www.ptglab.com/products/TUBB3-Antibody-10068-1-AP.htm?srsltid=AfmBOoq4wJHQEuLnvcwqyzOrati3jrRi7cY7NaH6QaXusVnoBCDItFho

Eukaryotic cell lines

Policy information about cell lines and Sex and Gender in Research

Cell line source(s)	The mouse breast cancer cell line EO771 was obtained from CH3 Biosystems. The mouse breast cancer cell line Py230, the murine melanoma cell line B16F10 and HEK293T was purchased from American Type Culture Collection (ATCC).
Authentication	All the cell lines were from reputable vendors and come with validation statements on the manufacturer's website. Cells were authenticated by short tandem repeat profiling, by morphology and in vivo and in vitro growth.
Mycoplasma contamination	All the cell lines were tested negative for mycoplasma contamination.
Commonly misidentified lines (See ICLAC register)	None.

Animals and other research organisms

Policy information about studies involving animals; ARRIVE guidelines recommended for reporting animal research, and Sex and Gender in Research

Laboratory animals	C57BL/6 and NOD/SCID mice were purchased from the Laboratory Animal Resource Center of Sun Yat-sen University. Chst4-CreERT2, Chst4-tdTomato, Alox12 flox/flox, R26-LSL-sLP-mCherry mice, conditional Chst4-CreERT2;R26-LSL-sLP-mCherry mice and conditional knockout Alox12fl/fl;Chst4-CreERT2 mice were constructed in Shanghai Model Organisms Center.
Wild animals	None.
Reporting on sex	Only female mice were used in all experiments.
Field-collected samples	At indicated time, mice were harvested and used for bioluminescence imaging, histology, gene expression and primary cell isolation.
Ethics oversight	All animal experiments were performed with approval of the Institutional Review Boards and Animal Care and Use Committees of Sun Yat-Sen University.

Note that full information on the approval of the study protocol must also be provided in the manuscript.

Plants

Seed stocks	*Report on the source of all seed stocks or other plant material used. If applicable, state the seed stock centre and catalogue number. If plant specimens were collected from the field, describe the collection location, date and sampling procedures.*
Novel plant genotypes	*Describe the methods by which all novel plant genotypes were produced. This includes those generated by transgenic approaches, gene editing, chemical/radiation-based mutagenesis and hybridization. For transgenic lines, describe the transformation method, the number of independent lines analyzed and the generation upon which experiments were performed. For gene-edited lines, describe the editor used, the endogenous sequence targeted for editing, the targeting guide RNA sequence (if applicable) and how the editor was applied.*
Authentication	*Describe any authentication procedures for each seed stock used or novel genotype generated. Describe any experiments used to assess the effect of a mutation and, where applicable, how potential secondary effects (e.g. second site T-DNA insertions, mosiacism, off-target gene editing) were examined.*

Flow Cytometry

Plots

Confirm that:

☒ The axis labels state the marker and fluorochrome used (e.g. CD4-FITC).

☒ The axis scales are clearly visible. Include numbers along axes only for bottom left plot of group (a 'group' is an analysis of identical markers).

☒ All plots are contour plots with outliers or pseudocolor plots.

☒ A numerical value for number of cells or percentage (with statistics) is provided.

Methodology

Sample preparation	To detect the photoconverted cells in lungs, lungs were minced into small pieces using surgical scissors and digested for 1 h at 37 °C with gentle rocking. Dissociated cell suspensions were then passed through 100 μm and 70 μm cell strainer

(BIOLOGIX, #15-1100 and #15-1070).
For cytotoxicity assays, mouse T cells were isolated from splens and CD8+ T cells were purified by CD8+ T Cell Isolation Kit (Miltenyi Biotec, #130-117-044). To generate mouse DCs, bone marrow cells from mice were isolated by flushing femurs and tibias and cultured in complete RPMI-1640 medium containing 20ng/ml GM-CSF (PeproTech, #315-03) and 10ng/ml IL-4 (PeproTech, #214-14).
To isolate blood vessel endothelial cells from mice, the inguinal fat pads, primary tumors and inguinal lymph nodes were lightly minced into small pieces and digested at 37 °C with gentle rocking. Dissociated cell suspensions were sequentially filtered through 100 and 40 µm cell strainers (BIOLOGIX, #15-1100 and #15-1040).
To isolate primary cells from patients, primary tumors and lymph nodes were lightly minced into small pieces and digested at 37 °C with gentle rocking. Dissociated cell suspensions were sequentially filtered through a 100 µm cell strainer (BIOLOGIX, #15-1100).
To analyze the activation of CD103+ DCs, CD8+ T cells, MDSCs and Treg cells in mouse mediastinal LNs, the mediastinal LNs were carefully dissected from wild-type C57BL/6 mice bearing BVM3 or HEVM3 tumor cells. Tissues were washed with Hanks Balanced Salt Solution three times and were lightly minced into small pieces. The LN tissues were digested with RPMI-1640 medium (GIBCO, #11875093) containing 0.2 mg/ml collagenase P (Sigma Aldrich, #11213857001), 0.8 mg/ml dispase ? (Sigma Aldrich, #D4693) and 0.01 mg/ml DNase I (Sigma Aldrich, #DN25) for 30 min at 37 °C under constant shaking. FBS (final concentration 30%) was added to stop the digestion. Dissociated cell suspensions were sequentially filtered through 40 µm cell strainers (BIOLOGIX #15-1040) to obtain a single-cell suspension.

Instrument	flow cytometer (BD Biosciences); CytoFLEX flow cytometer (Beckman Coulter).
Software	FlowJo (v10.0.7).
Cell population abundance	Purity of FACS-sorted samples was analysed by flow cytometry. Purity of the samples was greater than 95%.
Gating strategy	Starting cells were gated by FSC/SSC gates to select healthy, singlet populations. Standard forward scatter height versus area criteria were used to discard doublets and capture singlets. Gates indicating boundaries between 'positive' and 'negative' are according to the isotype staining. Expression of indicated proteins were checked on these populations as indicated in the figures and figure legends.

☒ Tick this box to confirm that a figure exemplifying the gating strategy is provided in the Supplementary Information.
