# Supplementary material for: Tumour-associated high endothelial venules drive portal-specific immune evasion in lymph nodes via ALOX12
Source: Nat Commun. 2026 May 11;17:6364. doi: 10.1038/s41467-026-72412-w (PMC13376640; doi:10.1038/s41467-026-72412-w)
Supplement: Supplementary file 9 — Transparent Peer Review file [file 41467_2026_72412_MOESM9_ESM.pdf]

nature portfolio

Peer Review File

Tumour-associated high endothelial venules drive portal-specific immune evasion in lymph nodes via ALOX12

Corresponding Author: Professor Shicheng Su

This file contains all reviewer reports in order by version, followed by all author rebuttals in order by version.

Attachments originally included by the reviewers as part of their assessment can be found at the end of this file.

Version 0:

Reviewer comments:

Reviewer #1

(Remarks to the Author)

Intravital microscopy conducted by different laboratories demonstrated that high endothelial venules (HEVs) in metastasis-containing lymph nodes facilitate the dissemination of tumor cells into the bloodstream. In addition, other investigators found that lymph node (LN) metastatic cells have a higher capacity to escape immunosurveillance due to chronic IFN signaling, resulting in enhanced distant metastases.
The authors confirm these observations in mouse models of breast cancer LN metastases and provide mechanistic data that HEVs enable the immunosuppressive features of tumor cells. They express the enzyme Alox 12 that produces the metabolite 12-HETE, which, upon uptake by tumor cells, binds to ADAR1 and “immunogenically silences” tumor cells through increased adenosine-to-isosine editing.
These data are very interesting, providing mechanistic insights that support the concept that tumor cells traveling through LNs are less immunogenic and have a higher capability to seed as distant metastases.
The authors utilized the elegant photoconvertible Dendra system to distinguish between tumor cells disseminating via lymph nodes (LNs) and other routes to seed in lung metastases, confirming that LN-derived tumor cells have a higher seeding capability in the lung. Although they propose that these are tumor cells that have disseminated via HEVs, they cannot exclude that these tumor cells disseminated via other blood vessels in the LN, or lymphatics. Can the authors clarify whether they performed lymphatic ligation to induce tumor cells to disseminate via the lymphatic blood vessels?
The authors provide scRNA-seq data showing that HEVs in LNs selectively express Alox12, further supporting their hypothesis that HEVs drive the immune recognition of tumor cells. The UMAP predominantly shows other cell types and includes the primary tumor, whereas the endothelial cell population appears very small and underrepresented. It would be more relevant to simply show a UMAP of the different endothelial cell populations in the LNs to increase the resolution of these cells, allowing for the observation of differences between non-HEVs, HEVs, and LECs. How many endothelial cells and HEVs were sequenced?
The authors claim that Alox 12 is specifically expressed in HEVs, but not in other endothelial cells. However, publicly available data sets from human lymph nodes (LNs) reveal that Alox 12 does not appear to be expressed in HEVs (doi: 10.1016/j.immuni.2019.06.027; includes LECs and BECs; doi: 10.1038/s41556-022-00866-3 sc-atlas of non-hematopoietic cells in human LNs). Further analysis of unpublished LN endothelial cells (about 20,000 cells) from our group revealed similar results in murine LNs, detecting Alox 12 in non-HEV blood endothelial cells, but not in HEVs (Chst4+, Fut7+) of LNs (see pdf).
Therefore, it appears that Alox 12 is predominantly expressed in blood endothelial cells in LNs. Could it be that HEVs undergo changes in metastatic LNs that induce Alox 12 expression? Still, the majority of Alox 12 would come from BECs.
The Alox 12 protein staining overlaps with HEVs; however, given that platelets express high levels of Alox 12, could this be due to platelet contamination? Given that 12-HETE is secreted, any cell that produces Alox 12 can contribute to the effects the authors observed.

Reviewer #2

(Remarks to the Author)

The manuscript by Qidong Xia and colleagues presents a highly compelling and mechanistically rich study that uncovers a novel role for high endothelial venule (HEV)-expressed ALOX12 in facilitating immune evasion of disseminating tumor cells via the production of 12-HETE. Through a combination of human patient data, sophisticated in vivo models, and deep molecular profiling, the authors convincingly demonstrate that HEV involvement correlates with poor prognosis in LN-positive breast cancer and that 12-HETE modulates ADAR1 p150-dsRNA liquid-liquid phase separation (LLPS), leading to increased A-to-I RNA editing and suppression of interferon signaling. The manuscript is thorough, methodologically sound, and of broad relevance across cancer biology and immunology. This manuscript provides a major advance in our understanding of tumor immune evasion and metastatic spread via HEVs. With the additional in vivo validation of the LLPS mechanism and contextualization in other tumor models, this paper will represent a significant contribution to the literature. I recommend acceptance after revision.
- While LLPS of ADAR1 p150 is well demonstrated in vitro, in vivo rescue experiments using ADAR1 p150 mutants (e.g., V677A/L681A) that disrupt 12-HETE-induced LLPS but not dsRNA binding would be critical to definitively establish the LLPS mechanism in metastasis and immune evasion.
- The findings are currently focused on breast cancer. The authors should evaluate whether similar ALOX12 expression and functional consequences occur in other LN-metastatic cancers (e.g., melanoma, lung cancer), either through publicly available datasets or limited experimental validation, to assess generalizability.
- The discussion raises an intriguing hypothesis that HEVs in TLSs may play a similar role. The manuscript posits HEVs in TLSs may serve similar roles in immune evasion, especially since TLSs are paradoxically associated with both better and worse prognosis in cancer. Preliminary experiments or analysis of TLSs in primary tumors for ALOX12 expression and/or 12-HETE-related signatures would significantly enhance the impact.
- It would be very relevant to include a time-course analysis or discussion on how rapidly immune suppression is established after tumor cells intravasate through HEVs.
- The experiments with the E861A editing-deficient mutant are very important. The authors should more clearly delineate whether LLPS alone (in the absence of editing) is sufficient to alter immune evasion.
- Authors should add quantification of MDA5/ZBP1 levels and localization in treated cells or tissue to confirm their suppression is downstream of RNA editing, not transcriptionally regulated.
- It is important to clearly distinguish HEVM vs. BVM terminology in the main text, and include a graphical overview of the photoconversion-based selection method.
- They should also correct minor typographical and grammatical inconsistencies throughout the text (e.g., spacing and punctuation in figure legends).

Reviewer #3

(Remarks to the Author)
In this study, Xia et. al., have investigated the involvement of high endothelial venules (HEV) in lymph nodes (LN) as an independent prognostic marker for LN-positive breast cancer. The authors report that ALOX12-derived 12-HETE, suppresses tumor immunogenicity via promoting A-to-I RNA editing by ADAR1. HEV involvement in tumor progression and roles of ALOX12/12-HETE and ADAR1 in immunosuppression have been explored separately before. However, the present study provides evidences for the molecular interactions and pathway crosstalk, linking the three distinct pathways with tumor progression. The authors have performed comprehensive investigations, including using patient samples, deep learning integration, animal models and different analytical approaches, to support their findings.

The following concerns need to be addressed.

1. Investigations are limited to sentinel lymph nodes (SLN) and axillary lymph nodes (ALN), did the authors look at tertiary lymphoid structures (TLS)? Authors briefly mention TLS but seems did not look at HEVs in TLS. Considering the depth of data they generated, authors should present data related to TLS.
2. Did the authors conduct any power calculations for the deep learning algorithm?
3. In figure legends, authors should clearly mention if the supplied image is representative and how many replicates were conducted for each experiment, where applicable.
4. Authors should clearly state if pathologist’s assessment was blinded against the algorithm predictions.
5. In showing associations of HEV involvement with survival parameters, Progression Free Survival (PFS) is perhaps more relevant than Disease Free Survival (DFS) since the authors are investigating disease progression/metastasis.
6. Serum ALOX12 levels have been previously reported at significantly higher levels in breast cancer patients with LN involvement, that was reduced following chemotherapy (Biochem Biophys Res Commun. 2011 PMID:1945939). Peripheral blood analysis on mouse models will strengthen the present study and increase potential clinical utilization.
7. In describing evasion of immune surveillance by HEV-migrant cells, did the authors look for immunosuppressive cells such as myeloid derived suppressor cells (MDSCs) and Tregs? Additional gating on CD11b+ MHCII- and Ly6Chigh/low can show the effects on immunosuppressive MDSCs and provide additional potential mechanism of immune evasion.
8. In discussion, please rectify that ALOX15 is an isoform of ALOX12 since both are distinct enzymes, encoded by different genes and not isoforms.
9. Overall, discussion section is weak. Authors must improve it to discuss additional roles of ALOX12 in tumor progression such as along the p53-ALOX12 axis (Nat. Cell Biol. 2019, PMID: 30962574), previous reports on ADAR1 as a therapeutic target, limitations of this study, effect of potential confounding factors on findings and potential further avenues for establishing ALOX12 as a therapeutic target should be discussed.
10. Gating strategies for some markers in flow cytometry is not convincing. For instance CD45, Granzyme B and PI staining. What sort of controls were used to define gates? Authors should provide data from controls such as isotype or fluorescence minus one (FMO) controls for flow cytometry.

11. What were the controls for microscopy images?
12. Authors should provide a list of 125 differentially regulated genes mentioned in Supplementary Figure 4b, along with p value (FDR) thresholds and fold change metrics.

Version 1:

Reviewer comments:

Reviewer #1

(Remarks to the Author)
The authors have incorporated several new experiments in response to the reviewers' comments and provided a thorough analysis. This may explain why some of the figures appear very small, making it difficult to clearly discern their contents. To improve clarity and visualization, some figure subsets could be moved to the Supplementary Materials.

Overall, the authors have now compiled a comprehensive body of work on HECs in lymph node (LN) metastases. However, two critical aspects still require clarification and further discussion:

HEC-Cherry Imaging Concerns (Fig. 3j):
The HEC images in Figure 3j are unusual as they lack a visible lumen and instead resemble clusters of accumulated cells. Moreover, there are only a few GFP^+ tumor cells, which should also display some Cherry positivity—even if the Cherry signal is secreted. Considering the tumor cell (TC) to HEC ratio, it seems unlikely that HECs would exhibit such strong Cherry positivity. Additionally, soluble Cherry should be taken up by other neighboring cells, particularly immune cells, which are abundant in the LN.
The authors are encouraged to include MECA79 staining to confirm HEVs and show their Cherry positivity in a separate image. Furthermore, please clarify which other cell types take up soluble Cherry and provide data on the incidence and timing of E0771 LN metastases.

Specificity of Alox12 Expression in HECs:
There remains concern regarding the specificity of Alox12 expression in HECs. Alox12 has been reported to be expressed across various capillary and arterial endothelial subclusters in LN endothelium, as also noted by the authors, but not specifically in HECs. The explanation that Alox12 is low-abundant and therefore undetected in HECs is not the case. However, it may hold true for the other EC subsets if the authors' datasets have a high gene expression dropout rate.

The hypothesis that Alox12 expression is induced or enhanced in HECs upon interaction with tumor cells is intriguing, particularly in the context of tumor-derived semaphorin 3C. It would be important to demonstrate whether such tumor cell–HEC interactions are indeed necessary for Alox12 induction, as this could explain the low frequency of $Alox12^+$ HECs observed.

However, the current data remain unconvincing. The authors report 44 LN HECs (similar to the small numbers of BECs and LECs). What percentage of these 44 HECs express Alox12? Can strong conclusions be drawn from such a limited sample size?

In response, the authors performed scRNA-seq on 1,870 BECs from metastatic LNs, an approach that is commendable and should have yielded a greater number of HECs. Yet, the UMAP visualization again reveals only a small HEC cluster. How many HECs were identified within the 1,870 BECs, and how many of them express Alox12?

This issue is critical, as robust data analysis of HECs requires a sufficient number of cells to ensure meaningful power. Without adequate representation, analyses of cell–cell interactions and other bioinformatic assessments may lack significance due to underpowered datasets.

Reviewer #2

(Remarks to the Author)
This revised manuscript by Xia et al. presents a compelling and mechanistically rich study revealing that high endothelial venules (HEVs) facilitate immune evasion of disseminating tumor cells through the ALOX12–12-HETE–ADAR1 axis. The work combines elegant imaging, deep-learning pathology, multi-omics, and genetic models to show that HEV involvement predicts poor prognosis in lymph-node–positive breast cancer and that HEV-derived 12-HETE enhances ADAR1-dependent A-to-I RNA editing to suppress interferon signaling.

The results are noteworthy for establishing a direct molecular connection between vascular biology, RNA modification, and tumor immune escape. The integration of human pathology, sophisticated in vivo labeling (notably the new sLP-mCherry system), and mechanistic validation provides a convincing demonstration that HEV-emigrant tumor cells are less immunogenic and more metastatic than those using other vascular routes.

The methodology is rigorous and well described, with appropriate use of conditional knockout mice, pharmacologic inhibition, and rescue experiments. The authors have addressed all prior concerns: they clarified the role of lymphatic ligation, provided new scRNA-seq data confirming predominant ALOX12 expression in HEVs of metastatic (but not pre-

metastatic) lymph nodes, and ruled out platelet contamination. The inclusion of full control data and quantitative validation significantly enhances reproducibility and transparency.

This study substantially advances our understanding of how lymph-node vasculature contributes to systemic metastasis and introduces a novel metabolite-driven mechanism of RNA editing–based immune suppression. The findings will be of wide interest across cancer biology, immunology, and RNA research.

Gian Gaetano Tartaglia

Reviewer #3

(Remarks to the Author)
Thank you for the revised manuscript and for addressing all comments, suggestions and concerns raised by the Reviewer. The authors have performed substantial revisions to the manuscript, improving the overall rigor and clarity of the work, while the newly added data further support their findings. However, the following concern still needs to be resolved and addressed properly.

-In response to previously raised comment 12, authors have provided a table (Supplementary Table 1) listing the 125 differentially expressed genes along with their Fisher's combined test statistics, used to rank genes upregulated in HECs relative to BECs and in tumor-bearing LN HECs compared to normal LN HECs. However, while the Fisher's combined statistic is useful in integrating significance across multiple scenarios, the underlying fold change and adjusted p-value metrics for each comparison to confirm the magnitude and direction of upregulation are still required for transparency, interpretability and reproducibility. The reported wide range of Fisher's combined test statistics in the Supplementary Table 1 also warrants verification.

Version 2:

Reviewer comments:

Reviewer #1

(Remarks to the Author)
The authors have added substantial data throughout the review period and have certainly improved their study. Although their argument that only 30 Alox 12+ HEVs could be yielded, because HEVs are a minor blood endothelial cell population in lymph nodes, is not correct; it is rather a major population, but more challenging to isolate, and there is still some concern about the Alox 12 specificity in HEVs, overall the authors have done impressive work with excellent techniques and tools, which merits publication.

Reviewer #3

(Remarks to the Author)
The Authors have addressed all comments and concerns raised by the Reviewer. The explanation regarding the sensitivity of Fisher's combined test to extreme p-values and expansion of Supplementary Table 1 to include key differential expression metrics have adequately resolved the remaining concern regarding analysis. Overall, the study provides important insights into the role of lymphatic endothelial venules in facilitating tumor immune evasive mechanisms, and presenting ALOX12 as a key mediator. The findings add mechanistic depth to our understanding of lymphatic–immune interactions in cancer.

Open Access This Peer Review File is licensed under a Creative Commons Attribution 4.0 International License, which permits use, sharing, adaptation, distribution and reproduction in any medium or format, as long as you give appropriate credit to the original author(s) and the source, provide a link to the Creative Commons license, and indicate if changes were made.
In cases where reviewers are anonymous, credit should be given to 'Anonymous Referee' and the source.
The images or other third party material in this Peer Review File are included in the article's Creative Commons license, unless indicated otherwise in a credit line to the material. If material is not included in the article's Creative Commons license and your intended use is not permitted by statutory regulation or exceeds the permitted use, you will need to obtain permission directly from the copyright holder.
To view a copy of this license, visit https://creativecommons.org/licenses/by/4.0/

A List of Major Additions in the Revised Manuscript:

1. We developed a new labelling sLP-mCherry system to distinguish HEV-emigrant tumour cells from those disseminating via LN lymphatics or other blood vessels, and confirmed their reduced immunogenicity and enhanced metastatic potential, thereby strengthening the conclusions of our study (Fig. 2h-j, Supplementary Fig. 3j, k).

2. To better visualize endothelial cells of our scRNA-seq data, we re-clustered endothelial cells into HECs, BECs and LECs using a high-resolution UMAP (Supplementary Fig. 4d) and observed the predominant expression of *Alox12* in LN HECs (Supplementary Fig. 4e).

3. We sorted BECs from mouse metastatic LNs and performed scRNA-seq, which confirmed that *Alox12* was predominantly expressed in HECs in metastatic LNs (Supplementary Fig. 5a, b).

4. A publicly available data set of human metastatic HNSCC LN metastasis confirmed the predominant expression of *ALOX12* and *PLXNA1* in HECs and *SEMA3C* in the tumour cells (Supplementary Fig. 7f).

5. Immunofluorescence pictures captured by high-resolution microscopy, flow cytometry and qRT-PCR showed that the basic expression of ALOX12 in LNs was mainly in HECs. Moderate ALOX12 expression was also detected in arterial LN ECs, albeit its level was much less than the one in HECs. ALOX12 level didn't increase in pre-metastatic LNs. Moreover, the expression of ALOX12 in HECs, rather the one in arterial LN ECs, was markedly elevated in metastatic LNs (Supplementary Fig. 5c-i).

6. Our revised data suggested that the tumour-derived SEMA3C may upregulate HEV ALOX12 expression through NRP2/PlexinA1 (Fig. 4 and Supplementary Fig. 7).

7. *In vivo* experiments using the ADAR1 p150 mutant (V677A/L681A) showed that HEV-conditional *Alox12* knockout significantly suppressed lung metastases and enhanced $CD8^+$ T and NK cells infiltration in the lung of KO + WT ADAR1 p150 EO771-bearing mice, but not in KO + ADAR1 p150 (V677A/L681A) EO771-bearing mice (Supplementary Fig. 11f, g).

8. A time-course analysis showed that both the vehicle-pretreated and 12-HETE-pretreated groups showed increased Granzyme B^+ $CD8^+$ T cell in the mediastinal LNs on day 3. However, the 12-HETE-pretreated group exhibited a markedly reduced level of Granzyme B^+ $CD8^+$ T cells compared to the vehicle-pretreated group (Supplementary Fig. 6k).

9. *In vivo* experiments using the ADAR1 p150 mutant (E861A) showed that HEV-specific *Alox12* deletion significantly suppressed lung metastasis and markedly enhanced infiltration of $CD8^+$ T cells and NK cells in the lung of mice bearing KO + WT ADAR1 p150 tumours, but not in mice bearing KO + ADAR1 p150 (E861A) tumours, despite intact LLPS capability (Supplementary Fig. 11l, m).

10. Immunofluorescence staining detected a reduction in the colocalization of MDA5

or ZBP1 with dsRNA in EO771 cells by 24 h after 12-HETE treatment and a decrease in the total protein levels of MDA5 and ZBP1 by 72 h after treatment (Supplementary Fig. 8a, b).

11. ELISA results showed that the serum level of SEMA3C in HEVM3 tumour-bearing mice was significantly higher than that in BVM3 tumour-bearing mice (Fig. 4c).

12. HEVM3 tumour-bearing mice harboured more $CD11b^{+}MHCII^{-}Ly6C^{hi/lo}$ MDSCs and $Foxp3^{+}CD4^{+}$ regulatory T (Treg) cells in mediastinal LNs compared with the mice bearing BVM3 tumours (Supplementary Fig. 3l, o-q).

13. We have provided data from isotype or FMO controls for flow cytometry plots (Fig. 2j, 3j, Supplementary Fig. 3e, 3n, 3p, 6k, 11k).

14. We have provided representative negative control images for key proteins of interest (ALOX12, MDA5, ZBP1 and J2) (Supplementary Fig. 4f, Supplementary Fig. 8b).

15. We provide a supplementary table listing all 125 differentially expressed genes along with their Fisher's combined test statistics (Supplementary Data 1).

Our Responses to Reviewers' Comments:

Reviewer #1 (Remarks to the Author):

Intravital microscopy conducted by different laboratories demonstrated that high endothelial venules (HEVs) in metastasis-containing lymph nodes facilitate the dissemination of tumor cells into the bloodstream. In addition, other investigators found that lymph node (LN) metastatic cells have a higher capacity to escape immunosurveillance due to chronic IFN signaling, resulting in enhanced distant metastases.

The authors confirm these observations in mouse models of breast cancer LN metastases and provide mechanistic data that HEVs enable the immunosuppressive features of tumor cells. They express the enzyme Alox12 that produces the metabolite 12-HETE, which, upon uptake by tumor cells, binds to ADAR1 and "immunogenically silences" tumor cells through increased adenosine-to-isosine editing.

These data are very interesting, providing mechanistic insights that support the concept that tumor cells traveling through LNs are less immunogenic and have a higher capability to seed as distant metastases.

Response: We would like to thank the reviewer for her/his enthusiastic comments.

1. The authors utilized the elegant photoconvertible Dendra system to distinguish between tumor cells disseminating via lymph nodes (LNs) and other routes to seed in lung metastases, confirming that LN-derived tumor cells have a higher seeding capability in the lung. Although they propose that these are tumor cells that have

disseminated via HEVs, they cannot exclude that these tumor cells disseminated via other blood vessels in the LN, or lymphatics. Can the authors clarify whether they performed lymphatic ligation to induce tumor cells to disseminate via the lymphatic blood vessels?

Response: We thank the reviewer for the insightful comment. In our previous manuscript, the ligation of efferent lymphatics was mentioned only in the context of enriching EO771-HEVM3 cells, as previously reported in the literature[1] to distinguish between tumour cells disseminating via LN blood vessels and those that do not. However, we did not perform lymphatic ligation in any of the other experiments. We apologize for the lack of clarity and have now explicitly stated that "ligation of efferent lymphatics was not conducted in other animal experiments" in the relevant sections of the methods (page 44, line 1235).

Moreover, to allay reviewer's concern regarding the possibility that tumour cells may disseminate via LN lymphatics or other blood vessels, we developed a new labelling system to specifically track HEV-associated tumour cells *in vivo*[2] (Fig. 2h). First, we constructed a line of *R26-LSL-sLP-mCherry* mice (C57BL/6J-Gt (ROSA)26Sor$^{em1(CAG\text{-}loxp\text{-}STOP\text{-}loxp\text{-}Kozak\text{-}SP\text{-}TATK\text{-}mCherry\text{-}NLS\text{-}WPRE\text{-}pA)}$) with expression of a secreted fluorescent mCherry protein containing a modified lipid-permeable transactivator of transcription (TATk) peptide (sLP-mCherry) under a stop codon. *Chst4-CreERT2* mice were crossed to *R26-LSL-sLP-mCherry* mice to obtain conditional *Chst4-CreERT2*;*R26-LSL-sLP-mCherry* mice. After tamoxifen treatment, the Cre recombinase translocated from the cytoplasm into the nucleus of HECs, where it excised the floxed stop cassette upstream of the sLP-mCherry gene. This conditional activation enables specific expression of the secreted fluorescent reporter sLP-mCherry exclusively in HEVs. We then engineered EO771 breast cancer cells to express the luciferase-GFP and injected luciferase-GFP-EO771 cells into the fat pad of conditional *Chst4-CreERT2*;*R26-LSL-sLP-mCherry* mice to induce LN metastases. sLP-mCherry protein secreted by HEVs was taken up by passing tumour cells (Supplementary Fig. 3j). This sLP-mCherry labelling system enabled specific discrimination of lung metastatic tumour cells disseminating via HEVs ($GFP^{+}mCherry^{+}$) compared with tumour cells disseminating via blood vessels or lymphatics ($GFP^{+}mCherry^{-}$) using fluorescenceactivated cell sorting (FACS) (Fig. 2i). The sLP-mCherry signal in $GFP^{+}mCherry^{+}$ tumour cells progressively decayed and disappeared within 5 days of *in vitro* culture. Consistent with our prior findings using the Dendra2 photoconversion method, $GFP^{+}mCherry^{+}$ tumour cells were more resistant to the T cell-mediated cytotoxicity *in vitro* compared with $GFP^{+}mCherry^{-}$ tumour cells (Fig. 2j). Moreover, $GFP^{+}mCherry^{+}$ and $GFP^{+}mCherry^{-}$ cells were injected into the mammary fat pad of mice. The results showed that $GFP^{+}mCherry^{+}$ cells exhibited greater metastatic potential than $GFP^{+}mCherry^{-}$ cells at humane end point (Supplementary Fig. 3k). Collectively, this sLP-mCherry labelling system allowed us to distinguish HEV-emigrant tumour cells from those disseminating via LN lymphatics or other blood vessels, and confirmed their reduced immunogenicity and enhanced metastatic potential, thereby strengthening the conclusions of our study.

2. The authors provide scRNA-seq data showing that HEVs in LNs selectively express Alox12, further supporting their hypothesis that HEVs drive the immune recognition of tumor cells. The UMAP predominantly shows other cell types and includes the primary tumor, whereas the endothelial cell population appears very small and underrepresented. It would be more relevant to simply show a UMAP of the different endothelial cell populations in the LNs to increase the resolution of these cells, allowing for the observation of differences between non-HEVs, HEVs, and LECs. How many endothelial cells and HEVs were sequenced?

Response: We agree with the reviewer. We excluded non-endothelial cell populations and re-clustered endothelial cells into HECs, BECs and LECs using a high-resolution UMAP[3] (Supplementary Fig. 4d). Consistently, we observed the predominant expression of *Alox12* in LN HECs (Supplementary Fig. 4e). The number of ECs in this data set was 187. The number of LN HECs, non-HEV LN BECs, LN LECs and tumour BECs were 44, 51, 38 and 54, respectively. The proportion of ECs in LN is 1.32%, which is in consistent to the previous report[4,5]. Our data are also in agreement with the previous scRNA-seq of the whole LNs, which also does not show lymphatic endothelial cells as a separate cluster in the UMAP plot when other non-endothelial cells are present (Figure 1B, the fourth panel[6]). The logic flow of this study is to use scRNA-seq to screen which cell types express *Alox12*. Therefore, we performed scRNA-seq on the cell suspension of the whole metastatic LNs, rather than the sample enriched for LN endothelial cells.

3. The authors claim that Alox12 is specifically expressed in HEVs, but not in other endothelial cells. However, publicly available data sets from human lymph nodes (LNs) reveal that Alox12 does not appear to be expressed in HEVs (doi: 10.1016/j.immuni.2019.06.027; includes LECs and BECs; doi: 10.1038/s41556-022-00866-3 sc-atlas of non-hematopoietic cells in human LNs). Further analysis of unpublished LN endothelial cells (about 20,000 cells) from our group revealed similar results in murine LNs, detecting Alox12 in non-HEV blood endothelial cells, but not in HEVs (Chst4+, Fut7+) of LNs (see pdf). Therefore, it appears that Alox12 is predominantly expressed in blood endothelial cells in LNs. Could it be that HEVs undergo changes in metastatic LNs that induce Alox12 expression? Still, the majority of Alox12 would come from BECs.

Response: Thank you very much for your careful validation of our findings. Our scRNA-seq data clearly demonstrated that *Alox12* was predominantly expressed in HECs in metastatic LNs. Moreover, we isolated 1870 $CD45^{-}CD31^{hi}$ BECs from metastatic LNs of 6 mice by FACs and performed scRNA-seq. Unsupervised analyses defined 8 BEC subsets with distinct gene expression: arterial ECs (Art); 2 venous EC subsets, high endothelial cells (HECs) and non-HEV veins (Vn); and five capillary phenotype EC subsets, including CapEC1, CapEC2, capillary resident progenitors (CRP), transitional ECs (TrECs), interferon stimulated gene-enriched CapECs (CapIfn)[7] (Supplementary Fig. 5a). Consistently, scRNA-seq showed that *Alox12* was

predominantly expressed in HECs in metastatic LNs (Supplementary Fig. 5b).

One possibility of the discrepancy is that publicly available data sets cited by the reviewer represent either non-metastatic LNs (doi: 10.1016/j.immuni.2019.06.027) or lymphoma-involved LNs (doi: 10.1038/s41556-022-00866-3), in which HEVs show of low ALOX12 expression. However, the predominant expression of *ALOX12* in HECs were corroborated by a publicly available scRNA-seq dataset of human metastatic LNs[8] (Supplementary Fig. 7f). It is also well documented that the gene expression drop-out and loss of signal in scRNA-seq often happen due to low expression of genes in rare cell types. Even the most sensitive scRNA-seq methods will struggle to detect low-abundance transcripts[9-13]. Therefore, scRNA-seq is a latest method to screen gene expression profiles, but not as reliable as qRT-PCR or immunofluorescence analysis to quantitate the levels of low-abundance transcripts.

Importantly, as the reviewer elegantly points out, our data indicate that HEVs may undergo dynamic changes in metastatic LNs and the *Alox12* expression could be modulated by tumour-derived factor(s). Indeed, we observed about 6-fold upregulation of *Alox12* in HEVs from metastatic LNs compared with homeostatic LNs (Supplementary Fig. 4h in the previous manuscript; Supplementary Fig. 4k in the revised manuscript). To evaluate the levels of ALOX12 in $CX40^{+}EMCN^{-}PNAd^{-}$ arterial LN ECs, $EMCN^{+}PNAd^{-}CX40^{-}$ capillary LN ECs and $PNAd^{+}CX40^{-}EMCN^{-}$ HECs in the whole sections of LNs by immunofluorescence, we injected EO771 cells expressed firefly luciferase *Luc2* gene into mouse fat pads. No *Luc2* signals were detected in draining LNs 11 days after inoculation as previously reported[14]. Thus, we defined these LNs as pre-metastatic LNs (Supplementary Fig. 5c). Our data showed that the basic expression of ALOX12 in LNs was mainly in HECs. Moderate ALOX12 level was also detected in arterial LN ECs. ALOX12 level didn't increase in pre-metastatic LNs. More importantly, the expression of ALOX12 in HECs, rather the one in arterial LN ECs, was markedly elevated in metastatic LNs (Supplementary Fig. 5d, e). Furthermore, these results were corroborated by flow cytometry (Supplementary Fig. 5f-h) and qRT-PCR analysis (Supplementary Fig. 5i). Therefore, we have toned down the statement from "ALOX12 is specifically expressed in LN HEVs" to "ALOX12 is predominantly expressed in HEVs of metastatic LNs".

Together, these data suggest that the expression of ALOX12 in HEVs in metastatic LNs may be regulated by tumour-derived factor(s). To identify the factor(s) that upregulate ALOX12, we analysed online mRNA profiling data of 90 metastatic LNs from breast cancer patients[15]. Among the top 5 secreted factors with the strongest correlation with *ALOX12* in metastatic LNs[16] (Supplementary Fig. 7a), deleting *Sema3c* in tumour cells rather than other factors reduced the expression of ALOX12 in HEVs of the HEVM3 tumour-bearing mice (Fig. 4a and Supplementary Fig. 7b-d). Of note, we observed no appreciable effect of SEMA3C knockout on LN metastases per se (Fig. 4a and Supplementary Fig. 7e). Enzyme-linked immunosorbent assay (ELISA) confirmed the overexpression of SEMA3C in derivatives that preferentially metastasize through HEVs from 3 different cell lines (Fig. 4b) and in the serum of corresponding tumour-bearing mice (Fig. 4c). Clinically, the proportion of $ALOX12^{+}$ HECs were significantly

correlated with the proportion of SEMA3C$^+$ tumour cells in human metastatic LNs (Fig. 4d, e).

Neuropilin 2 (NRP2) is the receptor of SEMA3C, and PlexinA1 is the functional coreceptor of SEMA3C[17]. Interestingly, we analysed cancer cell-to-HEC signals using our scRNA-seq data via CellChat[18] which allowed us to predict cellular interactions by linking ligand and receptor expression and found that *Sema3c*-(*Nrp2*+*Plxna1*) was one of the most significant ligand-receptor pairs (Fig. 4f). Correspondingly, scRNA-seq analysis revealed that *Sema3c* was almost exclusively expressed in cancer cells (Fig. 4g), whereas only HECs and macrophages expressed high levels of both *Nrp2* and *Plxna1* (Fig. 4h, i). In addition, the predominant expression of *ALOX12* and PLXNA1 in HECs and *SEMA3C* in the tumour cells were further corroborated by a publicly available scRNA-seq dataset of human metastatic LNs[8] (Supplementary Fig. 7f). Furthermore, treatment with the peptidic antagonist MTP-PlexA1 against PlexinA1 substantially suppressed ALOX12 expression in HECs (Fig. 4j) and lung metastases in the HEVM3 tumour-bearing mice (Fig. 4k). Collectively, these results suggest that tumour-derived SEMA3C may upregulate HEV ALOX12 expression through NRP2/PlexinA1.

In summary, while steady-state or lymphoma-associated LNs mainly show ALOX12 in non-HEV BECs, our integrative analyses demonstrate that ALOX12 is dynamically induced in HEVs of metastatic LNs by tumour-derived SEMA3C through NRP2/PlexinA1 signaling. This reconciles the apparent discrepancy and highlights a tumour-driven mechanism that remodels HEVs during metastasis.

4. The Alox12 protein staining overlaps with HEVs; however, given that platelets express high levels of Alox12, could this be due to platelet contamination?

Response: To exclude the potential influence of platelets on immunofluorescence staining, we perfused the mice before sampling[19-21]. Without perfusion, immunofluorescence staining detected a small number of platelets at the abluminal side of HEVs. Although platelets can express ALOX12 (1.72%), the majority of ALOX12 signal was highly co-localized with HEVs (95.64%). Importantly, following perfusion, no residual platelets were detected within HEVs, and ALOX12 staining remained confined to HEVs (96.69%) (Fig. 1 for the reviewer). These findings suggest that the ALOX12 signal observed in HEVs is not attributable to platelet contamination.

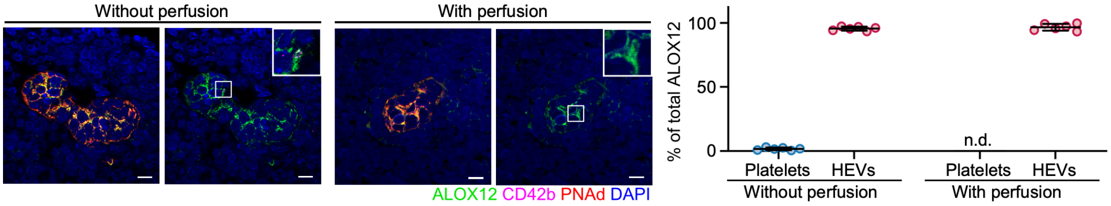

Figure 1 | The expression of ALOX12 in the LN HEVs and platelets. Representative immunofluorescence images (left) and quantitation (right) of the co-localization of ALOX12 (green) with HEVs marked by PNAd (red) and platelets marked by CD42b

(purple) in LNs without and with perfusion. Nuclei were stained with DAPI (blue). Zoomed-in images demonstrate platelet signals detected at the abluminal side of HEVs in non-perfused samples. Scale bars, 10 µm. n = 6 mice per group. Each dot represents data pooled from 5 distinct areas from a slice and 3 slices per sample. Mean ± SD.

5. Given that 12-HETE is secreted, any cell that produces Alox12 can contribute to the effects the authors observed.

Response: The reviewer raises a very intriguing issue that other sources of ALOX12 can contribute to the effects we observed. Indeed, as the reviewer elegantly points out, ALOX12 would also come from LN BECs. The rationales that we focus on HEVs are that HEVs are the major exit route by which tumour cells disseminate to circulation in LNs[1] and our evidence support that HEVs are the predominant and functionally indispensable source of ALOX12 in metastatic LNs (Fig. 2b, Fig. 3, Supplementary Fig. 4, Supplementary Fig. 5 in the previous manuscript; Fig. 2b, Fig. 3, Supplementary Fig. 4, Supplementary Fig. 5, Supplementary Fig. 6 in the revised manuscript). Most importantly, by using HEV-conditional knockout mice, our data unambiguously demonstrate the essential role of HEV ALOX12 in immune evasion of LN tumour escapees (Fig. 3h, Supplementary Fig. 5e, f in the previous manuscript; Fig. 3h, Supplementary Fig. 6e, f in the revised manuscript). Taken together, although other LN endothelial cells may contribute to ALOX12 expression at low levels, our results demonstrate that HEVs represent the predominant and functionally essential source of ALOX12 in metastatic LNs. We have provided relevant discussion in the revised manuscript (page 22, line 626-636) as below:

"In metastatic LNs, our comprehensive data demonstrates that ALOX12 is predominantly expressed in HEVs. It has been reported that blood vessel endothelial cells (BECs) in LNs can be categorized into several subsets[7]. We also observed that a small fraction of non-HEV BECs in LNs also express ALOX12, albeit its protein level is much less than the one in HEVs. However, HEVs are the major route for tumour cells to enter circulation in LNs[1] and our evidence support that HEVs are the predominant and functionally indispensable source of ALOX12 in metastatic LNs. Mechanistically, ALOX12 expression is enhanced in HEVs, rather than in non-HEV BECs, in metastatic LNs by SEMA3C. It is probably because PlexinA1, the functional co-receptor of SEMA3C, is expressed in HEVs but not non-HEV BECs, and the expression of NRP2, the receptor of SEMA3C, is higher in HECs compared with non-HEV LN BECs."

Reviewer #2 (Remarks to the Author):

The manuscript by Qidong Xia and colleagues presents a highly compelling and mechanistically rich study that uncovers a novel role for high endothelial venule

(HEV)-expressed ALOX12 in facilitating immune evasion of disseminating tumor cells via the production of 12-HETE. Through a combination of human patient data, sophisticated in vivo models, and deep molecular profiling, the authors convincingly demonstrate that HEV involvement correlates with poor prognosis in LN-positive breast cancer and that 12-HETE modulates ADAR1 p150-dsRNA liquid-liquid phase separation (LLPS), leading to increased A-to-I RNA editing and suppression of interferon signaling. The manuscript is thorough, methodologically sound, and of broad relevance across cancer biology and immunology. This manuscript provides a major advance in our understanding of tumor immune evasion and metastatic spread via HEVs. With the additional in vivo validation of the LLPS mechanism and contextualization in other tumor models, this paper will represent a significant contribution to the literature. I recommend acceptance after revision.

Response: We would like to thank the reviewer for finding our study "a highly compelling and mechanistically rich study which is supported by various techniques".

1. While LLPS of ADAR1 p150 is well demonstrated in vitro, in vivo rescue experiments using ADAR1 p150 mutants (e.g., V677A/L681A) that disrupt 12-HETE-induced LLPS but not dsRNA binding would be critical to definitively establish the LLPS mechanism in metastasis and immune evasion.

Response: We thank the reviewer for this very helpful suggestion. As suggested, we used KO + WT ADAR1 p150, KO + ADAR1 p150 (V677A/L681A), KO + WT ADAR1 p110 and KO + ADAR1 p110 (V429A/L433A) EO771 cells in orthotopic mouse models to assess their ability to evade immunity and form metastases. The results showed that HEV-conditional *Alox12* knockout significantly suppressed lung metastases (Supplementary Fig. 11f) and enhanced $CD8^+$ T and NK cells infiltration in the lung (Supplementary Fig. 11g) of KO + WT ADAR1 p150 EO771-bearing mice. Strikingly, these effects were not observed in KO + ADAR1 p150 (V677A/L681A) EO771-bearing mice (Supplementary Fig. 11f, g), indicating that disrupting 12-HETE-induced LLPS effectively abolishes its pro-metastatic and immunosuppressive effects. In contrast, there was no significant difference in metastatic burden or immune cell infiltration between the KO + WT ADAR1 p110 and KO + ADAR1 p110 (V429A/L433A) groups (Supplementary Fig. 11f, g), further supporting the isoform-specific role of ADAR1 p150 in LLPS. Taken together, these revised results demonstrate that the pro-metastatic and immunosuppressive effects of 12-HETE are critically dependent on its ability to induce LLPS of ADAR1 p150 *in vivo*, rather than p110 or other dsRNA-binding mechanisms.

2. The findings are currently focused on breast cancer. The authors should evaluate whether similar ALOX12 expression and functional consequences occur in other LN-metastatic cancers (e.g., melanoma, lung cancer), either through publicly available datasets or limited experimental validation, to assess generalizability.

Response: We thank the reviewer for this very helpful suggestion. To assess generalizability beyond breast cancer, we analyzed publicly available scRNA-seq data of human head and neck squamous cell carcinoma (HNSCC) metastatic LNs (GSE103322). Consistent with our findings, *ALOX12* was predominantly expressed in HECs (Supplementary Fig. 7f). Moreover, our revised data demonstrated that tumour-derived SEMA3C may upregulate HEV ALOX12 expression via NRP2/PlexinA1 (Fig. 4 and Supplementary Fig. 7). In line with this mechanism, the HNSCC dataset showed that *SEMA3C* was predominantly expressed in the tumour cells, while *PLXNA1*, the functional co-receptor of SEMA3C, was mainly expressed in HECs (Supplementary Fig. 7f). Moreover, by using HEV-conditional knockout mice, our data unambiguously demonstrate the essential role of HEV ALOX12 in immune evasion of LN tumour escapees in B16F10 cells, a model of melanoma (Supplementary Fig. 6e, j). Collectively, these results suggest that our findings on HEV ALOX12 are generalizable beyond breast cancer and HEV ALOX12 regulation by tumour-derived SEMA3C represents a general mechanism across LN-metastatic cancers.

3. The discussion raises an intriguing hypothesis that HEVs in TLSs may play a similar role. The manuscript posits HEVs in TLSs may serve similar roles in immune evasion, especially since TLSs are paradoxically associated with both better and worse prognosis in cancer. Preliminary experiments or analysis of TLSs in primary tumors for ALOX12 expression and/or 12-HETE-related signatures would significantly enhance the impact.

Response: Thank you for your insightful comment. Our previous study have shown that tertiary lymphoid structures (TLSs) are more frequently observed in breast cancers following chemotherapy compared with treatment-naive samples[22]. Moreover recent studies have highlighted that PNAd^{+} HEVs serve as a marker of mature TLSs[23-25], which are predominantly observed in patients achieving complete remission after chemotherapy[26]. As suggested, we stained HEVs for ALOX12 in TLSs of breast cancer primary tumours. We did not detect PNAd^{+} HEVs in TLSs of treatment-naïve or chemotherapy-resistant primary tumours (Fig. 2 for the reviewer). Moreover, ALOX12 expression in HEVs within mature TLSs of CR primary tumours was comparable to that in tumour-draining LNs, but significantly lower than in metastatic tumour-draining LNs (Fig. 2 for the reviewer). Our revised data demonstrated that tumour-derived SEMA3C upregulates HEV ALOX12 expression in metastatic LNs through NRP2/PlexinA1 (Fig. 4 and Supplementary Fig. 7). It stands to reason that although neoadjuvant chemotherapy leads to the appearance of mature TLSs in tumor beds of patients who achieve complete remission, HEVs within these TLSs exhibit minimal ALOX12 expression due to the absence of tumour-derived signals. As the reviewer elegantly points out, investigating the role of ALOX12 in TLSs, particularly its relationship with chemotherapy and the underlying mechanisms, is crucial. These are currently being investigated by us and also key areas of focus in our future research. We hope the reviewer would agree that need a future separate detailed study. We have expanded relevant discussion in the revised manuscript (page 22, line 616-625) as

below:

“Interestingly, HEVs can be found not only in LNs, but also in tertiary lymphoid structures (TLSs) in tumours. Given the unexplained association of TLSs with poor prognosis[27-29], it will be important to determine whether HEVs in TLSs play a role in tumour dissemination or modulate therapeutic responses. Previous studies indicate that mature TLSs containing PNAd$^+$ HEVs are more frequently observed in cancer patients after chemotherapy[23-25], especially in patients with complete remission after chemotherapy[26]. Thus, the potential roles of HEVs within TLSs, particularly their ALOX12 expression, in tumour progression remain unclear and warrant detailed mechanistic investigation in future studies.”

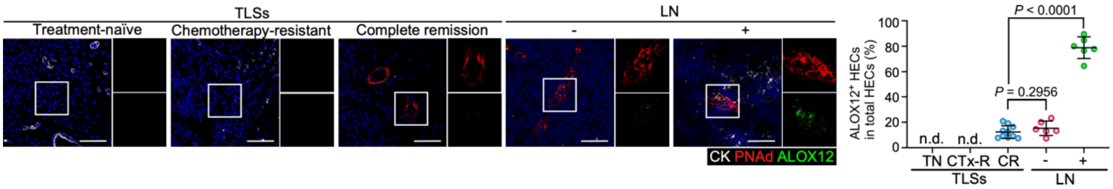

Figure 2 | The HEV ALOX12 expression in TLSs of primary tumour and LNs. Representative immunofluorescence images (left) and quantitation (right) of the proportion of ALOX12$^+$ HECs in treatment-naïve tumours (n = 3 patients), chemotherapy-resistant tumours (n = 3 patients), complete remission tumours (n = 10 patients), tumour draining LNs (n = 6 patients) and metastatic tumour draining LNs (n = 6 patients). Scale bars, 50 μm. Each dot represents data pooled from 5 distinct areas from a slice and 3 slices per patient. Mean ± SD. *P* value was calculated by one-way ANOVA with Tukey’s multiple comparisons test. TN, treatment-naïve; CTx-R, chemotherapy-resistant; CR, complete remission; n.d., not detected.

4. It would be very relevant to include a time-course analysis or discussion on how rapidly immune suppression is established after tumor cells intravasate through HEVs.

Response: We thank the reviewer for this very helpful suggestion. To mimic the scenario in which tumour cells intravasate through HEVs into the bloodstream, we pretreated tumour cells with 12-HETE *in vitro* and injected them via the tail vein. This approach models the exposure of tumour cells to HEV-derived 12-HETE prior to their entry into circulation. As suggested, we conducted a time-course analysis to assess the activation of Granzyme B$^+$ CD8$^+$ T cells in the mediastinal LNs on days 1, 2, and 3 after tumour cell injection. The results showed that on days 1 and 2, Granzyme B$^+$ CD8$^+$ T cells were not significantly activated in either group (Supplementary Fig. 6k). On day 3, both the vehicle-pretreated and 12-HETE-pretreated groups showed increased Granzyme B$^+$ CD8$^+$ T cell levels, reflecting the onset of the adaptive immune response. However, the 12-HETE-pretreated group exhibited a markedly reduced level of Granzyme B$^+$ CD8$^+$ T cells compared to the vehicle-pretreated group (Supplementary Fig. 6k), suggesting that HEV-secreted 12-HETE lowers the immunogenicity of tumour

cells and dampens subsequent T cell activation, contributing to immune suppression during the early phase of metastatic dissemination.

5. The experiments with the E861A editing-deficient mutant are very important. The authors should more clearly delineate whether LLPS alone (in the absence of editing) is sufficient to alter immune evasion.

Response: We thank the reviewer for this very helpful suggestion. As suggested, we employed KO + WT ADAR1 p150 EO771 cells and KO + ADAR1 p150 (E861A) EO771 cells in orthotopic mouse models to evaluate their capacity for immune evasion and metastatic potential. In mice bearing KO + WT ADAR1 p150 tumours, HEV-specific *Alox12* deletion significantly suppressed lung metastasis (Supplementary Fig. 11l) and markedly enhanced infiltration of $CD8^+$ T cells and NK cells in the lung (Supplementary Fig. 11m). In contrast, these effects were abrogated in mice bearing KO + ADAR1 p150 (E861A) tumours, despite intact LLPS capability (Supplementary Fig. 11l, m). These findings demonstrate that 12-HETE-induced LLPS of ADAR1 p150 is not sufficient by itself to drive immune evasion. Instead, A-to-I RNA editing mediated by the catalytic deaminase domain is indispensable, serving as a prerequisite for suppression of MDA5/ZBP1-driven interferon signaling. This result further underscores the functional importance of RNA editing downstream of LLPS in regulating tumour immune escape.

6. Authors should add quantification of MDA5/ZBP1 levels and localization in treated cells or tissue to confirm their suppression is downstream of RNA editing, not transcriptionally regulated.

Response: It is well documented that both MDA5 and ZBP1 are IFN-stimulated genes (ISGs), with their expression regulated by IFNs[30,31]. To investigate whether 12-HETE affects MDA5/ZBP1 protein levels and localization, we performed immunofluorescence staining at 24 h, 48 h, and 72 h after 12-HETE treatment. At 24 h, we observed a reduction in the colocalization of MDA5 or ZBP1 with dsRNA in EO771 cells, suggesting impaired recognition of dsRNA (Supplementary Fig. 8a, b). However, the total protein levels of MDA5 and ZBP1 showed a decrease by 72 h after treatment (Supplementary Fig. 8a, b). These findings indicate that 12-HETE initially induces RNA editing, which disrupts the integrity of dsRNA and thereby inhibits the activation of MDA5/ZBP1-mediated IFN response. Subsequently, as tumour-derived IFN levels decline, the transcription of MDA5 and ZBP1 is further downregulated. Thus, 12-HETE-induced RNA editing appears to be the primary mechanism underlying immune suppression.

7. It is important to clearly distinguish HEVM vs. BVM terminology in the main text, and include a graphical overview of the photoconversion-based selection method.

Response: We thank the reviewer for this very helpful suggestion. As suggested, we revised the text to clearly distinguish HEV-emigrant metastatic cells (HEVM) from Blood Vessel Metastatic (BVM) in the main text (page 7, line 186-189) as follows:

"To enrich tumour cells that preferentially spread via LN blood vessels, we repeated the *in vivo* selection by harvesting green^{+}red^{+} HEV metastatic (HEVM) tumour cells and green^{+}red^{-} blood vessel metastatic (BVM) tumour cells from lung metastases."

We have previously provided a graphical abstract illustrating the photoconversion-based cell tracking method in the Supplementary Fig. 2d in the previous manuscript. For clarity, we have moved the graphical abstract to the main figure (Fig. 2c) in the revised manuscript to facilitate reading.

8. They should also correct minor typographical and grammatical inconsistencies throughout the text (e.g., spacing and punctuation in figure legends).

Response: We thank the reviewer for his/her careful reading, and we have revised the grammatical errors throughout the manuscript in the revised manuscript.

Reviewer #3 (Remarks to the Author):

In this study, Xia et. al., have investigated the involvement of high endothelial venules (HEV) in lymph nodes (LN) as an independent prognostic marker for LN-positive breast cancer. The authors report that ALOX12-derived 12-HETE, suppresses tumor immunogenicity via promoting A-to-I RNA editing by ADAR1. HEV involvement in tumor progression and roles of ALOX12/12-HETE and ADAR1 in immunosuppression have been explored separately before. However, the present study provides evidences for the molecular interactions and pathway crosstalk, linking the three distinct pathways with tumor progression. The authors have performed comprehensive investigations, including using patient samples, deep learning integration, animal models and different analytical approaches, to support their findings.

Response: We would like to thank the reviewer for her/his enthusiastic comments.

The following concerns need to be addressed.

1. Investigations are limited to sentinel lymph nodes (SLN) and axillary lymph nodes (ALN), did the authors look at tertiary lymphoid structures (TLS)? Authors briefly mention TLS but seems did not look at HEVs in TLS. Considering the depth of data they generated, authors should present data related to TLS.

Response: We agree with the reviewer and please refer to the response to Reviewer 2,

point 3. Our previous study have shown that tertiary lymphoid structures (TLSs) are more frequently observed in breast cancers following chemotherapy compared with treatment-naive samples[22]. Moreover recent studies have highlighted that PNAd$^+$ HEVs serve as a marker of mature TLSs[23-25], which are predominantly observed in patients achieving complete remission after chemotherapy[26]. As suggested, we stained HEVs for ALOX12 in TLSs of breast cancer primary tumours. We did not detect PNAd$^+$ HEVs in TLSs of treatment-naïve or chemotherapy-resistant primary tumours (Fig. 2 for the reviewer). Moreover, ALOX12 expression in HEVs within mature TLSs of CR primary tumours was comparable to that in tumour-draining LNs, but significantly lower than in metastatic tumour-draining LNs (Fig. 2 for the reviewer). Our revised data demonstrated that tumour-derived SEMA3C upregulates HEV ALOX12 expression in metastatic LNs through NRP2/PlexinA1 (Fig. 4 and Supplementary Fig. 7). It stands to reason that although neoadjuvant chemotherapy leads to the appearance of mature TLSs in tumor beds of patients who achieve complete remission, HEVs within these TLSs exhibit minimal ALOX12 expression due to the absence of tumour-derived signals. As the reviewer elegantly points out, investigating the role of ALOX12 in TLSs, particularly its relationship with chemotherapy and the underlying mechanisms, is crucial. These are currently being investigated by us and also key areas of focus in our future research. We hope the reviewer would agree that need a future separate detailed study. We have expanded relevant discussion in the revised manuscript (page 22, line 616-625) as below:

"Interestingly, HEVs can be found not only in LNs, but also in tertiary lymphoid structures (TLSs) in tumours. Given the unexplained association of TLSs with poor prognosis[27-29], it will be important to determine whether HEVs in TLSs play a role in tumour dissemination or modulate therapeutic responses. Previous studies indicate that mature TLSs containing PNAd$^+$ HEVs are more frequently observed in cancer patients after chemotherapy[23-25], especially in patients with complete remission after chemotherapy[26]. Thus, the potential roles of HEVs within TLSs, particularly their ALOX12 expression, in tumour progression remain unclear and warrant detailed mechanistic investigation in future studies."

2. Did the authors conduct any power calculations for the deep learning algorithm?

Response: Thank you for your insightful comment. In deep learning studies, power calculations are not commonly applied as in traditional statistical research. Power calculations are typically used to determine sample size sufficiency, but in deep learning, the focus is on improving model performance, architecture, and application outcomes. Therefore, consistent with previous studies on deep learning model[32,33], we adopted area under the curve (AUC) score to tested the performance of the classification network using an independent validation set. Although we did not conduct a formal power analysis, our sample size is comparable to, and even larger than, those used in similar deep learning models in previous studies[34,35].

3. In figure legends, authors should clearly mention if the supplied image is representative and how many replicates were conducted for each experiment, where applicable.

Response: Thank you for your careful reading. We have revised all figure legends to specify whether images are representative and indicate the number of biological and technical replicates.

4. Authors should clearly state if pathologist's assessment was blinded against the algorithm predictions.

Response: We thank the reviewer for this very helpful suggestion and have added the corresponding information in the revised manuscript (page 43, line 1203-1205) as follows:

"All participating pathologists had at least five years of experience in breast cancer slide diagnosis, with some practicing for over 30 years, and were blinded to the patients' clinical background."

5. In showing associations of HEV involvement with survival parameters, Progression Free Survival (PFS) is perhaps more relevant than Disease Free Survival (DFS) since the authors are investigating disease progression/metastasis.

Response: We thank the reviewer for this valuable comment. We agree that progression-free survival (PFS) is more commonly used to assess disease progression or metastasis in the context of ongoing treatments, particularly in late-stage or metastatic cancer. However, all patients we collected in the three cohorts were treat-naïve and early-stage breast cancer patients who have undergone surgery. Disease-free survival (DFS) refers to the time from the completion of treatment to the recurrence of the disease (such as relapse or metastasis) or death. DFS focuses more on the 'disease-free' status after treatment and is commonly used to assess the long-term health status of patients who have undergone curative treatment, typically in early-stage cancer patients. Therefore, consistent with previous studies on prognostic markers[36,37], we analyzed the associations of HEV involvement with DFS in our study.

6. Serum ALOX12 levels have been previously reported at significantly higher levels in breast cancer patients with LN involvement, that was reduced following chemotherapy (Biochem Biophys Res Commun. 2011 PMID:1945939). Peripheral blood analysis on mouse models will strengthen the present study and increase potential clinical utilization.

Response: We thank the reviewer for highlighting the Singh, Abhay Kumar, et al.

(PMID: 21945939) paper. Although the originally provided PMID appears to be incorrect, we were able to identify the correct article based on the context. We agree with the reviewer that peripheral blood analysis on mouse models would strengthen the present study and increase its potential clinical utilization. As suggested, we measured the serum levels of ALOX12 in mouse models. However, we did not detect ALOX12 in the serum of tumour-bearing mice. It is well documented that ALOX12 is a cytoplasmic protein, which may only enter the serum only in small amounts under cell damage, lysis or via non-classical secretion mechanisms[38,39]. In the literature mentioned by the reviewer, the authors considered ALOX12 in the serum to be secreted by tumour cells, though the mechanism was not clarified. Our revised data showed that tumour cells express the secretory factor SEMA3C, which promotes the expression of ALOX12 in HEVs (Fig. 4 and Supplementary Fig. 7). To further enhance the potential clinical utilization, we evaluated the serum levels of SEMA3C in tumour-bearing mice. The results showed that the serum level of SEMA3C in HEVM3 tumour-bearing mice was significantly higher than that in BVM3 tumour-bearing mice (Fig. 4c). These findings suggest that detecting tumour-derived SEMA3C in serum could have potential clinical significance.

7. In describing evasion of immune surveillance by HEV-migrant cells, did the authors look for immunosuppressive cells such as myeloid derived suppressor cells (MDSCs) and Tregs? Additional gating on CD11b+ MHCII- and Ly6Chigh/low can show the effects on immunosuppressive MDSCs and provide additional potential mechanism of immune evasion.

Response: We thank the reviewer for this very helpful suggestion. As suggested, we additional gated on $CD11b^{+}MHCII^{-}Ly6C^{hi/lo}$ myeloid-derived suppressor cells (MDSCs) in mediastinal LNs. The results showed that the mice bearing HEVM3 tumours harboured more $CD11b^{+}MHCII^{-}Ly6C^{hi/lo}$ MDSCs in mediastinal LNs compared with the mice bearing BVM3 tumours (Supplementary Fig. 3l, o). Additionally, we performed further flow cytometry analysis to identify $Foxp3^{+}CD4^{+}$ regulatory T (Treg) cells in mediastinal LNs. The results also showed that the mice bearing HEVM3 tumours harboured more Treg cells in mediastinal LNs (Supplementary Fig. 3p, q). Collectively, these findings suggest that IFNs play multifaceted roles in anti-tumour immunity, such as promoting the activity of $CD8^{+}$ T cells, natural killer (NK) cells and DCs, while inhibiting the activity of Treg cells and MDSCs[40].

8. In discussion, please rectify that ALOX15 is an isoform of ALOX12 since both are distinct enzymes, encoded by different genes and not isoforms.

Response: We thank the reviewer for pointing this out. We have revised the discussion to clearly state that ALOX12 and ALOX15 are distinct enzymes encoded by separate genes in the revised manuscript (page 23, line 646-648) as follows:

"Of note, ALOX15, another lipoxygenase family member, was reported to be expressed by some human breast cancer cells and propagate lymphatic metastasis via 12-HETE."

9. Overall, discussion section is weak. Authors must improve it to discuss additional roles of ALOX12 in tumor progression such as along the p53-ALOX12 axis (Nat. Cell Biol. 2019, PMID: 30962574), previous reports on ADAR1 as a therapeutic target, limitations of this study, effect of potential confounding factors on findings and potential further avenues for establishing ALOX12 as a therapeutic target should be discussed.

Response: We thank the reviewer for this insightful comment and we have expanded relevant discussion in the revised manuscript as follows:

1) Additional roles of ALOX12 in tumour progression such as along the p53-ALOX12 axis (page 24, line 663-671):
"ALOX12 has also been reported to be essential for p53-mediated tumour suppression through a distinct ferroptosis pathway[41]. Our findings provide an additional level of understanding of ALOX12 biology by demonstrating that elevated ALOX12 expression in HEVs contributes to immune evasion and promotes metastatic progression. Importantly, the mechanism that we identified depends on its enzymatic product, 12-HETE. Previous studies have shown that 12-HETE is involved in broader tumour-promoting mechanisms[42-44]. Thus, our results do not contradict existing literature but instead highlight the substrate- and context-dependent roles of ALOX12."
2) Previous reports on ADAR1 as a therapeutic target (page 24, line 688-697):
"Numerous studies using independent genetic evidence from various cancer cell types have demonstrated that perturbation of ADAR1 reduces cancer cell viability *in vitro* and sensitizes tumours to immunotherapy *in vivo* by restoring the dsRNA sensing pathway[45-48], highlighting ADAR1 as a promising therapeutic target. In addition, a novel small-molecule inhibitor, ZYS-1, has been developed, demonstrating strong antitumour activity together with an acceptable safety profile. Collectively, these findings indicate that ADAR1 is druggable and that ADAR1 inhibitors may hold broad therapeutic potential across diverse cancers[49]. Our study adds mechanistic depth to this concept by elucidating the role of LLPS in modulating ADAR1 activity."
3) Limitations of this study (page 25, line 706-712; page 22, line 616-625):
"Our preliminary data suggest that 12-HETE binds to ADAR1 p150 and may promote ADAR1 p150-dsRNA phase separation through a mechanism known as polyphasic linkage[50]. This observation raises the possibility that 12-HETE facilitates LLPS of ADAR1 p150 in a ligand-dependent manner. To elucidate the molecular basis and mode of interaction between 12-HETE and ADAR1 p150, the future use of tools such as cryo-electron microscopy (cryo-EM) would be highly valuable."

"Interestingly, HEVs can be found not only in LNs, but also in tertiary lymphoid structures (TLSs) in tumours. Given the unexplained association of TLSs with poor prognosis[27-29], it will be important to determine whether HEVs in TLSs play a role in tumour dissemination or modulate therapeutic responses. Previous studies indicate that mature TLSs containing PNAd^{+} HEVs are more frequently observed in cancer patients after chemotherapy[23-25], especially in patients with complete remission after chemotherapy[26]. Thus, the potential roles of HEVs within TLSs, particularly their ALOX12 expression, in tumour progression remain unclear and warrant detailed mechanistic investigation in future studies."

4) Effect of potential confounding factors on findings (page 22, line 626-636):
   "In metastatic LNs, our comprehensive data demonstrates that ALOX12 is predominantly expressed in HEVs. It has been reported that BECs in LNs can be categorized into several subsets[7]. We also observed that a small fraction of non-HEV BECs in LNs also express ALOX12, albeit its protein level is much less than the one in HEVs. However, HEVs are the major route for tumour cells to enter circulation in LNs[1] and our evidence support that HEVs are the predominant and functionally indispensable source of ALOX12 in metastatic LNs. Mechanistically, ALOX12 expression is enhanced in HEVs, rather than in non-HEV BECs, in metastatic LNs by SEMA3C. It is probably because PlexinA1, the functional co-receptor of SEMA3C, is expressed in HEVs but not non-HEV BECs, and the expression of NRP2, the receptor of SEMA3C, is higher in HECs compared with non-HEV LN BECs."
5) Potential further avenues for establishing ALOX12 as a therapeutic target (page 23, line 638-645):
   "Despite the existence of small-molecule ALOX12 inhibitors such as Baicalein and ML355, their clinical translation has been limited[51-53]. Our study identifies SEMA3C-NRP2/PlexinA1 as an upstream regulatory axis that drives ALOX12 in HEVs within metastatic LNs. This finding suggests that targeting this ligand-receptor pair, for example through neutralizing antibodies against SEMA3C or PlexinA1, may represent a more feasible and therapeutically actionable approach to modulate ALOX12 activity in a tissue- and context-specific manner."

10. Gating strategies for some markers in flow cytometry is not convincing. For instance CD45, Granzyme B and PI staining. What sort of controls were used to define gates? Authors should provide data from controls such as isotype or fluorescence minus one (FMO) controls for flow cytometry.

Response: We thank the reviewer for this very helpful suggestion. As suggested, we provided data from isotype or FMO controls for flow cytometry plots. The PI^{+} gate was defined using the unstimulated T cell control to account for background apoptosis and non-specific cytotoxicity (Fig. 2j, 3j, Supplementary Fig. 3e, 11k). An isotype-matched IgG antibody was used as a negative control for CD45 gating (Supplementary Fig. 3n, p). A FMO control was used to define the threshold for Granzyme B staining in CD45^{+}CD3^{+}CD8^{+} T cells (Supplementary Fig. 3n, 6k) and Foxp3 staining in

$CD45^+CD3^+CD4^+$ T cells (Supplementary Fig. 3p).

11. What were the controls for microscopy images?

Response: We thank the reviewer for raising this important point. All primary antibodies used in this study have been validated by the manufacturers for immunofluorescence applications. To ensure the specificity of our immunofluorescence staining, we used irrelevant isotype-matched control antibodies as negative controls. We have provided representative negative control images for key proteins of interest (ALOX12, MDA5, ZBP1 and J2) in our revised manuscript (Supplementary Fig. 4f, Supplementary Fig. 8b). Furthermore, we have revised the Methods section to explicitly describe the inclusion of isotype controls in our immunofluorescence procedures (page 48, line 1352-1355) as follows:

"Isotype-matched control antibodies were used in parallel as negative controls, and the mean fluorescence intensity from isotype controls was used to establish background levels and set thresholds for quantitative immunofluorescence analysis."

12. Authors should provide a list of 125 differentially regulated genes mentioned in Supplementary Figure 4b, along with p value (FDR) thresholds and fold change metrics.

Response: We thank the reviewer for raising this important point. We provide a supplementary table listing all 125 differentially expressed genes along with their Fisher's combined test statistics, which were used to rank genes upregulated in HECs relative to BECs and further increased in tumour-bearing LN HECs compared with normal LN HECs (Supplementary Data 1).

1 Brown, M. *et al.* Lymph node blood vessels provide exit routes for metastatic tumor cell dissemination in mice. *Science* **359**, 1408-1411 (2018).

2 Ombrato, L. *et al.* Metastatic-niche labelling reveals parenchymal cells with stem features. *Nature* **572**, 603-608 (2019).

3 Parker, K. R. *et al.* Single-cell analyses identify brain mural cells expressing CD19 as potential off-tumor targets for CAR-T immunotherapies. *Cell* **183**, 126-142. e117 (2020).

4 Veerman, K., Tardiveau, C., Martins, F., Coudert, J. & Girard, J.-P. Single-cell analysis reveals heterogeneity of high endothelial venules and different regulation of genes controlling lymphocyte entry to lymph nodes. *Cell reports* **26**, 3116-3131. e3115 (2019).

5 Malhotra, D. *et al.* Transcriptional profiling of stroma from inflamed and resting lymph nodes defines immunological hallmarks. *Nature immunology* **13**, 499-510 (2012).

6 Xu, K. *et al.* Single-cell RNA sequencing reveals cell heterogeneity and transcriptome profile of breast cancer lymph node metastasis. *Oncogenesis* **10**,

66 (2021).
7 Brulois, K. *et al.* A molecular map of murine lymph node blood vascular endothelium at single cell resolution. *Nature communications* **11**, 3798 (2020).
8 Puram, S. V. *et al.* Single-cell transcriptomic analysis of primary and metastatic tumor ecosystems in head and neck cancer. *Cell* **171**, 1611-1624. e1624 (2017).
9 Lu, T. *et al.* Overcoming expressional drop-outs in lineage reconstruction from single-cell RNA-sequencing data. *Cell Reports* **34**, 108589 (2021).
10 Papalexi, E. & Satija, R. Single-cell RNA sequencing to explore immune cell heterogeneity. *Nature Reviews Immunology* **18**, 35-45 (2018).
11 Kolodziejczyk, A. A., Kim, J. K., Svensson, V., Marioni, J. C. & Teichmann, S. A. The technology and biology of single-cell RNA sequencing. *Molecular cell* **58**, 610-620 (2015).
12 Potter, S. S. Single-cell RNA sequencing for the study of development, physiology and disease. *Nature Reviews Nephrology* **14**, 479-492 (2018).
13 Linderman, G. C. *et al.* Zero-preserving imputation of single-cell RNA-seq data. *Nature communications* **13**, 192 (2022).
14 Riedel, A., Shorthouse, D., Haas, L., Hall, B. A. & Shields, J. Tumor-induced stromal reprogramming drives lymph node transformation. *Nature immunology* **17**, 1118-1127 (2016).
15 Waldron, L. *et al.* Expression profiling of archival tumors for long-term health studies. *Clinical cancer research* **18**, 6136-6146 (2012).
16 Berdiel-Acer, M. *et al.* Stromal NRG1 in luminal breast cancer defines pro-fibrotic and migratory cancer-associated fibroblasts. *Oncogene* **40**, 2651-2666 (2021).
17 Tamagnone, L. *et al.* Plexins are a large family of receptors for transmembrane, secreted, and GPI-anchored semaphorins in vertebrates. *Cell* **99**, 71-80 (1999).
18 Jin, S. *et al.* Inference and analysis of cell-cell communication using CellChat. *Nature communications* **12**, 1088 (2021).
19 Jafarnejad, M. *et al.* Quantification of the whole lymph node vasculature based on tomography of the vessel corrosion casts. *Scientific Reports* **9**, 13380 (2019).
20 Paavonsalo, S., Subashi, Y., Lackman, M. H. & Karaman, S. Whole-mount immunofluorescence staining of blood and lymphatic vessels in murine small intestine. *STAR protocols* **4**, 102310 (2023).
21 Whittington, N. C. & Wray, S. Suppression of red blood cell autofluorescence for immunocytochemistry on fixed embryonic mouse tissue. *Current protocols in Neuroscience* **81**, 2.28. 21-22.28. 12 (2017).
22 Lu, Y. *et al.* Complement signals determine opposite effects of B cells in chemotherapy-induced immunity. *Cell* **180**, 1081-1097. e1024 (2020).
23 Teillaud, J.-L., Houel, A., Panouillot, M., Riffard, C. & Dieu-Nosjean, M.-C. Tertiary lymphoid structures in anticancer immunity. *Nature Reviews Cancer* **24**, 629-646 (2024).
24 Meylan, M. *et al.* Tertiary lymphoid structures generate and propagate anti-tumor antibody-producing plasma cells in renal cell cancer. *Immunity* **55**, 527-541. e525 (2022).
25 Wang, Z. *et al.* Spatial and maturity heterogeneity of tertiary lymphoid structures

shapes immune microenvironment and progression in prostate cancer. *Journal of the National Cancer Center* (2025).
26 Song, I. H. *et al.* Predictive value of tertiary lymphoid structures assessed by high endothelial venule counts in the neoadjuvant setting of triple-negative breast cancer. *Cancer research and treatment: official journal of Korean Cancer Association* **49**, 399-407 (2017).
27 Finkin, S. *et al.* Ectopic lymphoid structures function as microniches for tumor progenitor cells in hepatocellular carcinoma. *Nature immunology* **16**, 1235-1244 (2015).
28 Bento, D. C. *et al.* High endothelial venules are rare in colorectal cancers but accumulate in extra-tumoral areas with disease progression. *Oncoimmunology* **4**, e974374 (2015).
29 Figenschau, S. L., Fismen, S., Fenton, K. A., Fenton, C. & Mortensen, E. S. Tertiary lymphoid structures are associated with higher tumor grade in primary operable breast cancer patients. *BMC cancer* **15**, 1-11 (2015).
30 Kang, D.-c. *et al.* mda-5: An interferon-inducible putative RNA helicase with double-stranded RNA-dependent ATPase activity and melanoma growth-suppressive properties. *Proceedings of the National Academy of Sciences* **99**, 637-642 (2002).
31 Yang, Z.-H. *et al.* ZBP1 senses splicing aberration through Z-RNA to promote cell death. *Molecular Cell* **85**, 1775-1789. e1775 (2025).
32 Zhang, Z. *et al.* Pathologist-level interpretable whole-slide cancer diagnosis with deep learning. *Nature Machine Intelligence* **1**, 236-245 (2019).
33 Campanella, G. *et al.* Clinical-grade computational pathology using weakly supervised deep learning on whole slide images. *Nature medicine* **25**, 1301-1309 (2019).
34 Wang, X. *et al.* Predicting gastric cancer outcome from resected lymph node histopathology images using deep learning. *Nature communications* **12**, 1637 (2021).
35 Bejnordi, B. E. *et al.* Diagnostic assessment of deep learning algorithms for detection of lymph node metastases in women with breast cancer. *Jama* **318**, 2199-2210 (2017).
36 Giuliano, A. E. *et al.* Axillary dissection vs no axillary dissection in women with invasive breast cancer and sentinel node metastasis: a randomized clinical trial. *Jama* **305**, 569-575 (2011).
37 Song, Y. J. *et al.* The role of lymphovascular invasion as a prognostic factor in patients with lymph node-positive operable invasive breast cancer. *Journal of breast cancer* **14**, 198-203 (2011).
38 Kristensen, S. R. Mechanisms of cell damage and enzyme release. *Danish Medical Bulletin* **41**, 423-433 (1994).
39 Chen, R., Kang, R. & Tang, D. The mechanism of HMGB1 secretion and release. *Experimental & molecular medicine* **54**, 91-102 (2022).
40 Parker, B. S., Rautela, J. & Hertzog, P. J. Antitumour actions of interferons: implications for cancer therapy. *Nature Reviews Cancer* **16**, 131-144 (2016).

41 Chu, B. *et al.* ALOX12 is required for p53-mediated tumour suppression through a distinct ferroptosis pathway. *Nature cell biology* **21**, 579-591 (2019).
42 Nieves, D. & Moreno, J. J. Enantioselective effect of 12 (S)-hydroxyeicosatetraenoic acid on 3T6 fibroblast growth through ERK 1/2 and p38 MAPK pathways and cyclin D1 activation. *Biochemical pharmacology* **76**, 654-661 (2008).
43 Borin, T. F., Angara, K., Rashid, M. H., Achyut, B. R. & Arbab, A. S. Arachidonic acid metabolite as a novel therapeutic target in breast cancer metastasis. *International journal of molecular sciences* **18**, 2661 (2017).
44 Chen, Y. Q. *et al.* Endogenous 12 (S)-HETE production by tumor cells and its role in metastasis. *Cancer research* **54**, 1574-1579 (1994).
45 Bhate, A., Sun, T. & Li, J. B. ADAR1: a new target for immuno-oncology therapy. *Molecular cell* **73**, 866-868 (2019).
46 Ishizuka, J. J. *et al.* Loss of ADAR1 in tumours overcomes resistance to immune checkpoint blockade. *Nature* **565**, 43-48 (2019).
47 Liu, H. *et al.* Tumor-derived IFN triggers chronic pathway agonism and sensitivity to ADAR loss. *Nature medicine* **25**, 95-102 (2019).
48 Gannon, H. S. *et al.* Identification of ADAR1 adenosine deaminase dependency in a subset of cancer cells. *Nature communications* **9**, 5450 (2018).
49 Wang, X. *et al.* Targeting ADAR1 with a small molecule for the treatment of prostate cancer. *Nature Cancer* **6**, 474-492 (2025).
50 Wyman, J. & Gill, S. J. Ligand-linked phase changes in a biological system: applications to sickle cell hemoglobin. *Proceedings of the National Academy of Sciences* **77**, 5239-5242 (1980).
51 Ehses, J. A. & Donath, M. Y. Targeting 12-lipoxygenase as a novel strategy to combat the effects of inflammation on beta cells in diabetes. *Diabetologia* **58**, 425-428 (2015).
52 Luci, D. *et al.* Discovery of ML355, a potent and selective inhibitor of human 12-lipoxygenase. *Probe Reports from the NIH Molecular Libraries Program [Internet]* (2014).
53 Zhang, L., Li, C., Lin, G., Krajcsi, P. & Zuo, Z. Hepatic metabolism and disposition of baicalein via the coupling of conjugation enzymes and transporters—in vitro and in vivo evidences. *The AAPS journal* **13**, 378-389 (2011).

A List of Major Additions in the Revised Manuscript:

1. MECA79 (PNAd) staining verified that mCherry$^+$ structures with visible lumens in LNs correspond to PNAd$^+$ HEVs. We also showed their mCherry positivity in a separate image, confirming that sLP-mCherry was predominantly localized to PNAd$^+$ HEVs, and that the secreted sLP-mCherry could be taken up by adjacent tumour cells (Supplementary Fig. 3n).
2. Immunofluorescence analysis on lymph nodes harvested at an early time point showed that HEVs located in close proximity to tumour cells exhibited strong ALOX12 staining, whereas those distant from tumour regions displayed minimal ALOX12 levels (Supplementary Fig. 8f).
3. TurboID-based proximity labeling approach revealed that NRP2 and PlexinA1 were the most prominently enriched biotinylated proteins in HECs from metastatic LNs of mice bearing SEMA3C-TurboID-overexpressing EO771 cells (Fig. 4j, k and Supplementary Data 2).
4. We have expanded Supplementary Data 1 to include the underlying log2 fold changes and adjusted p-values for each individual comparison with each comparison presented in a separate worksheet (Supplementary Data 1).

Our Responses to Reviewers' Comments:

Reviewer #1 (Remarks to the Author):

The authors have incorporated several new experiments in response to the reviewers' comments and provided a thorough analysis. This may explain why some of the figures appear very small, making it difficult to clearly discern their contents. To improve clarity and visualization, some figure subsets could be moved to the Supplementary Materials.

Response: We thank the reviewer for the helpful comment. In the revised version, we have optimized the figure layout to enhance readability. Specifically, the previous Supplementary Fig. 3 has been split into Supplementary Figs. 3 and 4, and several secondary panels showing validation or control experiments from Fig. 2 have been relocated to the Supplementary Figures (Supplementary Fig. 3g, o, p and Supplementary Fig. 4c). This restructuring provided additional space to enlarge the panels in Fig. 2 and Supplementary Figs. 3 and 4, substantially improving visualization. We believe this adjustment substantially improves the overall clarity of data presentation.

Overall, the authors have now compiled a comprehensive body of work on HECs in lymph node (LN) metastases. However, two critical aspects still require clarification and further discussion:

HEC-Cherry Imaging Concerns (Fig. 3j):

The HEC images in Figure 3j are unusual as they lack a visible lumen and instead resemble clusters of accumulated cells. Moreover, there are only a few GFP$^+$ tumor cells, which should also display some Cherry positivity—even if the Cherry signal is secreted. Considering the tumor cell (TC) to HEC ratio, it seems unlikely that HECs would exhibit such strong Cherry positivity. Additionally, soluble Cherry should be taken up by other neighboring cells, particularly immune cells, which are abundant in the LN.

The authors are encouraged to include MECA79 staining to confirm HEVs and show their Cherry positivity in a separate image. Furthermore, please clarify which other cell types take up soluble Cherry and provide data on the incidence and timing of E0771 LN metastases.

Response: We appreciate the reviewer's insightful comments. The structural appearance of HEVs may vary depending on sectioning angles and perivascular cell accumulation in metastatic LNs, which may obscure luminal morphology in some images. To confirm the identity of HEVs, we performed MECA79 (PNAd) staining and verified that mCherry$^+$ structures with visible lumens in LNs correspond to PNAd$^+$ HEVs (Supplementary Fig. 3n). In *Chst4-CreERT2*;*R26-LSL-sLP-mCherry* mice, tamoxifen-induced Cre activation drives HEV-specific expression of the secreted fluorescent reporter sLP-mCherry. Immunofluorescence analysis confirmed that sLP-mCherry was predominantly localized to PNAd$^+$ HEVs, and that the secreted sLP-mCherry could be taken up by adjacent tumour cells (Supplementary Fig. 3n).

As suggested, we further examined the distribution of soluble sLP-mCherry by flow cytometry in immune cells. Some sLP-mCherry signals were predominantly detected in small fractions of CD3$^+$ T cells and CD19$^+$ B cells (Figure 1 for the reviewer), likely reflecting the fact that HEVs serve as the primary entry route for circulating T and B lymphocytes. Importantly, this observation does not compromise our conclusions, as tumour cells were independently and specifically labeled with GFP, enabling unambiguous discrimination of tumour cells from lymphocyte populations (Fig. 2h).

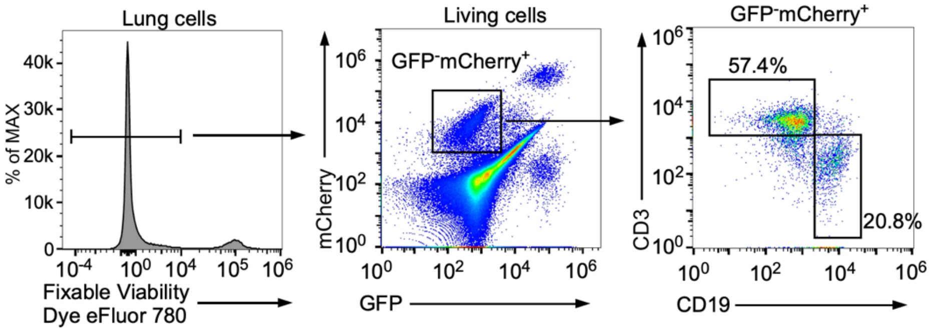

Figure 1 for the reviewer | Distribution of soluble sLP-mCherry among immune cells in the lung. Flow cytometric analysis of soluble sLP-mCherry distribution among immune cell populations (GFP$^-$mCherry$^+$) isolated from the lung.

Finally, we have provided data on the incidence and timing of EO771 LN metastases in

the previous manuscript (Supplementary Fig. 6c). EO771 cells expressing the firefly luciferase *Luc2* gene were injected into the mammary fat pads of mice. As previously reported[1], *Luc2* signals were detected in the draining LNs of all mice (100%) 14 days after inoculation, and these LNs were defined as metastatic. To ensure consistency and adequate metastatic progression, LN tissues were collected on day 20 after EO771 inoculation. The corresponding information has been provided in the Methods section in the previous manuscript (Line 1221, 1372 in the previous manuscript; Line 1260, 1411 in the revised manuscript).

Specificity of Alox12 Expression in HECs:

There remains concern regarding the specificity of Alox12 expression in HECs. Alox12 has been reported to be expressed across various capillary and arterial endothelial subclusters in LN endothelium, as also noted by the authors, but not specifically in HECs. The explanation that Alox12 is low-abundant and therefore undetected in HECs is not the case. However, it may hold true for the other EC subsets if the authors' datasets have a high gene expression dropout rate.

Response: First, we carefully verified the quality of our single-cell datasets and confirmed that they are technically sound. In our single-cell sequencing of mouse metastatic LNs and isolated BECs, we did not detect obvious *Alox12* expression in capillary or arterial endothelial cells, which differs from previously reported datasets. This discrepancy may be attributed to differences in mouse models or experimental contexts. Notably, our findings are supported by an additional publicly available dataset generated in the context of solid tumours, showing the predominant expression of *ALOX12* and *PLXNA1* in HECs and *SEMA3C* in the tumour cells in metastatic LNs[2] (Supplementary Fig. 8g).

We fully agree that *Alox12* can be expressed at variable levels across various capillary and arterial endothelial subclusters in LN endothelium. Nonetheless, our focus on HEV-derived ALOX12 is based on three considerations: 1) Previous studies[3] and our own findings have established that HEVs are the primary route through which tumour cells enter circulation from LNs (Fig. 2b). 2) Our evidence demonstrates that tumour-derived semaphorin 3C (SEMA3C) specifically promotes ALOX12 expression in tumour-associated HECs, making HEVs the predominant ALOX12 source in metastatic LNs (Fig. 4 and Supplementary Fig. 8). 3) Using HEV-conditional knockout mice, we directly showed that HEV ALOX12 is functionally indispensable for immune evasion of LN tumour escapees (Fig. 3h, Supplementary Fig. 7e, f).

Accordingly, the central conclusion of our study is that HEVs are dynamically reprogrammed by tumour-derived signals to acquire a pro-metastatic, immune-evasive function. In this context, ALOX12 expression in HEVs is induced in a tumour-associated and spatially restricted manner. To better reflect this functional and context-dependent perspective, we have revised the manuscript title from “High endothelial venule ALOX12 drives portal-specific immune evasion of tumour cells” to “Tumour-

associated high endothelial venules drive portal-specific immune evasion in lymph nodes via ALOX12". Throughout the revised manuscript, we consistently emphasize that ALOX12 expression in HEVs is tumour-associated and spatially restricted, underscoring biological specificity rather than uniform endothelial expression.

The hypothesis that Alox12 expression is induced or enhanced in HECs upon interaction with tumor cells is intriguing, particularly in the context of tumor-derived semaphorin 3C. It would be important to demonstrate whether such tumor cell–HEC interactions are indeed necessary for Alox12 induction, as this could explain the low frequency of Alox12^{+} HECs observed.

Response: Our previous data showed that ALOX12 expression in HECs, rather than in arterial LN endothelial cells, was markedly elevated in metastatic but not in pre-metastatic lymph nodes, indicating that tumour cell-HEC interactions are indeed required for ALOX12 induction. To further substantiate this notion, we performed immunofluorescence analysis on lymph nodes harvested at an early time point (2 weeks after EO771 implantation), when only sparse metastatic tumour cells were present. The results showed that HEVs located in close proximity to tumour cells exhibited strong ALOX12 staining, whereas those distant from tumour regions displayed minimal ALOX12 levels (Supplementary Fig. 8f). These findings suggest that ALOX12 upregulation in HEVs is not constitutive but induced by tumour-derived cues, which also explains the low frequency of ALOX12^{+} HEVs observed in scRNA-seq datasets, reflecting their spatial restriction rather than technical limitations.

To further substantiate the role of tumour-derived SEMA3C in regulating HEV ALOX12 expression within metastatic lymph nodes, we employed a TurboID-based proximity labeling approach coupled with liquid chromatography-tandem mass spectrometry (LC-MS/MS). EO771 cells expressing an SEMA3C-TurboID fusion protein were implanted into the mammary fat pad, and upon LN metastasis formation, intraperitoneal injection of exogenous biotin was performed to biotinylate proteins in close vicinity to SEMA3C-TurboID. Primary HECs were then isolated from metastatic LNs, and TurboID-labeled proteins interacting with tumour-derived SEMA3C were identified by LC-MS/MS (Fig. 4j). Notably, NRP2 and PlexinA1 were the most prominently enriched biotinylated proteins in HECs from metastatic LNs of mice bearing SEMA3C-TurboID-overexpressing EO771 cells, indicating that tumor-derived SEMA3C is spatially proximal to and engages NRP2/PlexinA1 signaling on HEVs *in vivo* (Fig. 4k). Importantly, treatment with the peptidic antagonist MTP-PlexA1 against PlexinA1 substantially suppressed ALOX12 expression in tumour-associated HECs (Fig. 4l). The results revealed the proximity of SEMA3C to its receptors NRP2 and PlexinA1 on HEVs, supporting a model in which tumour-derived SEMA3C engages NRP2/PlexinA1 signaling to induce ALOX12 expression in tumour-associated HEVs.

In line with the reviewer's insightful comment, our data strongly support the notion that

ALOX12 expression in HEVs requires direct interactions with nearby tumour cells and is driven by tumour-derived SEMA3C signaling. This spatially restricted induction explains why ALOX12 expression is predominantly observed in HEVs within metastatic lymph nodes and only in a subset of HEVs. ALOX12^{+} HEVs represent those in close proximity to and actively engaged by transiting tumour cells, highlighting a critical tumour-endothelial interaction that underlies further distant metastasis and constitutes a central focus of our study.

We have added relevant discussion in the revised manuscript (line 662-670) as below:

"Together, these findings reveal a spatially restricted yet functionally critical feedback loop between metastatic tumour cells and the LN HECs. However, mechanistic dissection of the downstream signaling pathways remains challenging, as primary HECs cannot be stably cultured or maintained *ex vivo*[4]. This technical limitation precludes conventional *in vitro* manipulation and signaling assays. Therefore, future studies employing conditional genetic mouse models will be essential to further elucidate the molecular cascade by which tumour-derived SEMA3C activates the NRP2/PlexinA1-ALOX12 axis in tumour-associated HEVs within metastatic lymph nodes."

However, the current data remain unconvincing. The authors report 44 LN HECs (similar to the small numbers of BECs and LECs). What percentage of these 44 HECs express Alox12? Can strong conclusions be drawn from such a limited sample size?

In response, the authors performed scRNA-seq on 1,870 BECs from metastatic LNs, an approach that is commendable and should have yielded a greater number of HECs. Yet, the UMAP visualization again reveals only a small HEC cluster. How many HECs were identified within the 1,870 BECs, and how many of them express Alox12?

This issue is critical, as robust data analysis of HECs requires a sufficient number of cells to ensure meaningful power. Without adequate representation, analyses of cell–cell interactions and other bioinformatic assessments may lack significance due to underpowered datasets.

Response: Among 44 LN HECs identified in our single-cell sequencing of mouse metastatic LNs, 68.18% (30 of 44) expressed *Alox12*. In our single-cell sequencing of isolated LN BECs, 6.46% (122 of 1,870) were annotated as HECs. Notably, 31.96% (39 of 122) of these HECs expressed *Alox12*.

Importantly, to complement the scRNA-seq analyses and avoid reliance on limited cell numbers, we quantified HEV-tumour cell associations at the tissue level. In whole-section analyses of mouse metastatic lymph nodes, 43.67 ± 7.29% of HEVs were directly associated with tumour cells among total HEVs (Fig. 2b). Consistently, in breast cancer patient metastatic lymph nodes, 34.76 ± 14.05% HEVs were involved with tumor cells among total HEVs in the whole slices (Figure 2 for reviewer). Together,

these spatial and quantitative analyses provide independent evidence supporting the functional relevance of HEV-tumour interactions for elevated ALOX12 expression in tumour-associated HEVs within metastatic lymph nodes.

We agree with the reviewer that ALOX12 is not uniformly expressed across all HEVs. Our data instead support a model in which ALOX12 is selectively induced in tumour-associated HEVs, which represent the functionally relevant subset involved in metastatic dissemination. Importantly, as demonstrated by our mechanistic data, HEV-derived ALOX12 produces 12-HETE that suppresses tumour cell immunogenicity, thereby enabling tumour cells in close proximity to these HEVs to evade immune surveillance and subsequently disseminate to distant organs. Thus, the spatial restriction of ALOX12^{+} HEVs reflects biological specificity rather than technical limitation and is central to its pro-metastatic function.

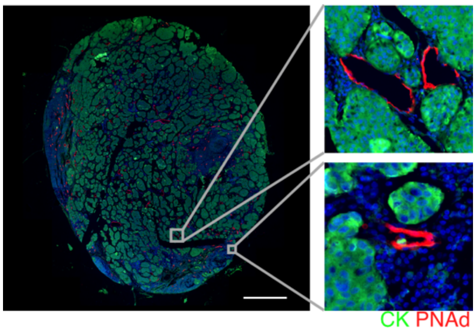

Figure 2 for the reviewer | Tumor cells intravasate via HEVs in breast cancer patient metastatic LNs. Representative immunofluorescence staining for PNAd and cytokeratin (CK) in human metastatic LNs. Scale bars, 500 μm.

We also acknowledge the reviewer's concern regarding the relatively small HEC population in scRNA-seq datasets. This is, however, a limitation in lymph node HEC studies, given that PNAd^{+} HEVs represent only a small fraction of the total LN endothelial compartment, especially in inflammatory or tumour contexts[5-9]. Indeed, a previous study that systematically characterized HEC phenotypic diversity, heterogeneity and plasticity in different conditions also analyzed a relatively small number of isolated endothelial cells (220 in total: 79 homeostatic HECs, 44 dedifferentiated HECs, 65 inflamed HECs, and 32 BECs). Despite this limited number, the authors successfully revealed substantial transcriptional and spatial heterogeneity of HECs and their dynamic remodeling in response to inflammation and antigenic stimulation[8]. Consistent with this precedent, the limited number of HECs captured in our dataset likely reflects the intrinsic rarity of PNAd^{+} endothelium within the total LN endothelial population rather than under-sampling of our data.

We have revised the relevant discussion in the revised manuscript (line 645-654) as

below:

“HEVs exhibit remarkable phenotypic diversity, heterogeneity, and plasticity, undergoing extensive remodeling in inflamed LNs and tumour-draining LNs[5-9]. However, the direct interactions between HECs and tumour cells in metastatic LNs remain poorly understood. In this study, we identified that ALOX12 expression in tumour-associated HEVs is dynamically induced by tumour-derived SEMA3C in metastatic LNs. Although HECs are intrinsically rare and thus underrepresented in scRNA-seq datasets, a common limitation in lymph node endothelial studies[8], our findings are supported by multiple complementary approaches. Notably, ALOX12 upregulation is not uniform across all HEVs but is selectively enriched in tumour-associated HEVs, which represent the functionally relevant subset.”

Reviewer #2 (Remarks to the Author):

This revised manuscript by Xia et al. presents a compelling and mechanistically rich study revealing that high endothelial venules (HEVs) facilitate immune evasion of disseminating tumor cells through the ALOX12–12-HETE–ADAR1 axis. The work combines elegant imaging, deep-learning pathology, multi-omics, and genetic models to show that HEV involvement predicts poor prognosis in lymph-node–positive breast cancer and that HEV-derived 12-HETE enhances ADAR1-dependent A-to-I RNA editing to suppress interferon signaling.

The results are noteworthy for establishing a direct molecular connection between vascular biology, RNA modification, and tumor immune escape. The integration of human pathology, sophisticated in vivo labeling (notably the new sLP-mCherry system), and mechanistic validation provides a convincing demonstration that HEV-emigrant tumor cells are less immunogenic and more metastatic than those using other vascular routes.

The methodology is rigorous and well described, with appropriate use of conditional knockout mice, pharmacologic inhibition, and rescue experiments. The authors have addressed all prior concerns: they clarified the role of lymphatic ligation, provided new scRNA-seq data confirming predominant ALOX12 expression in HEVs of metastatic (but not pre-metastatic) lymph nodes, and ruled out platelet contamination. The inclusion of full control data and quantitative validation significantly enhances reproducibility and transparency.

This study substantially advances our understanding of how lymph-node vasculature contributes to systemic metastasis and introduces a novel metabolite-driven mechanism of RNA editing–based immune suppression. The findings will be of wide interest across cancer biology, immunology, and RNA research.

Response: We are grateful to the reviewer for recognizing the conceptual advance and experimental rigor of our study. We appreciate her/his supportive comments highlighting the novelty and robustness of our findings.

Reviewer #3 (Remarks to the Author):

Thank you for the revised manuscript and for addressing all comments, suggestions and concerns raised by the Reviewer. The authors have performed substantial revisions to the manuscript, improving the overall rigor and clarity of the work, while the newly added data further support their findings. However, the following concern still needs to be resolved and addressed properly.

In response to previously raised comment 12, authors have provided a table (Supplementary Table 1) listing the 125 differentially expressed genes along with their Fisher's combined test statistics, used to rank genes upregulated in HECs relative to BECs and in tumor-bearing LN HECs compared to normal LN HECs. However, while the Fisher's combined statistic is useful in integrating significance across multiple scenarios, the underlying fold change and adjusted p-value metrics for each comparison to confirm the magnitude and direction of upregulation are still required for transparency, interpretability and reproducibility. The reported wide range of Fisher's combined test statistics in the Supplementary Table 1 also warrants verification.

Response: We thank the reviewer for pointing out the need for greater transparency and verification of the combined statistics. Upon careful re-examination of the data, we identified two technical issues that we have now corrected.

First, the initial list of 125 upregulated genes contained duplicate entries arising from multiple Ensembl gene IDs mapping to the same gene symbol. After collapsing duplicated Ensembl IDs and retaining unique gene symbols, the final list comprises 113 upregulated genes. Second, we found that the wide range of Fisher's combined test statistics is primarily attributable to our analytical strategy of focusing on genes that are consistently upregulated across multiple comparisons. We inherently enriched for genes with very small p-values in at least one comparison. As Fisher's combined statistic is highly sensitive to extreme p-values, this pre-selection naturally results in a broad dynamic range of combined statistics. Importantly, this reflects biological consistency across conditions rather than analytical artifacts. In addition, extremely small adjusted p-values (adjusted p-values of 0) further contribute to the upper range of the combined statistic. To avoid infinite values in log-transformed statistics prior to Fisher's combination, adjusted p-values of 0 were set to 1e-300, which resulted in large values after log transformation.

Importantly, this correction does not alter the overall ranking trends or biological conclusions.

In addition, we have expanded Supplementary Data 1 to include the underlying log2 fold changes and adjusted p-values for each individual comparison with each comparison presented in a separate worksheet. This allows direct inspection of both the magnitude and direction of differential expression underlying the Fisher's combined statistics.

Reference

1 Riedel, A., Shorthouse, D., Haas, L., Hall, B. A. & Shields, J. Tumor-induced stromal reprogramming drives lymph node transformation. *Nature immunology* **17**, 1118-1127 (2016).

2 Puram, S. V. *et al.* Single-cell transcriptomic analysis of primary and metastatic tumor ecosystems in head and neck cancer. *Cell* **171**, 1611-1624. e1624 (2017).

3 Brown, M. *et al.* Lymph node blood vessels provide exit routes for metastatic tumor cell dissemination in mice. *Science* **359**, 1408-1411 (2018).

4 Lacorre, D.-A. *et al.* Plasticity of endothelial cells: rapid dedifferentiation of freshly isolated high endothelial venule endothelial cells outside the lymphoid tissue microenvironment. *Blood* **103**, 4164-4172 (2004).

5 Vella, G., Hua, Y. & Bergers, G. High endothelial venules in cancer: Regulation, function, and therapeutic implication. *Cancer Cell* (2023).

6 Jones, D. *et al.* Solid stress impairs lymphocyte infiltration into lymph-node metastases. *Nature biomedical engineering* **5**, 1426-1436 (2021).

7 Milutinovic, S., Abe, J., Godkin, A., Stein, J. V. & Gallimore, A. The dual role of high endothelial venules in cancer progression versus immunity. *Trends in cancer* **7**, 214-225 (2021).

8 Veerman, K., Tardiveau, C., Martins, F., Coudert, J. & Girard, J.-P. Single-cell analysis reveals heterogeneity of high endothelial venules and different regulation of genes controlling lymphocyte entry to lymph nodes. *Cell reports* **26**, 3116-3131. e3115 (2019).

9 Blanchard, L. & Girard, J.-P. High endothelial venules (HEVs) in immunity, inflammation and cancer. *Angiogenesis* **24**, 719-753 (2021).

<u>Our Responses to Reviewers' Comments:</u>

Reviewer #1 (Remarks to the Author):

The authors have added substantial data throughout the review period and have certainly improved their study. Although their argument that only 30 Alox 12+ HEVs could be yielded, because HEVs are a minor blood endothelial cell population in lymph nodes, is not correct; it is rather a major population, but more challenging to isolate, and there is still some concern about the Alox12 specificity in HEVs, overall the authors have done impressive work with excellent techniques and tools, which merits publication.

Response: We thank the reviewer for the positive evaluation of our work and for recognizing the conceptual advance and experimental rigor of this study. Moreover, following the reviewer's comment, we have revised the wording in the Discussion to clarify that HECs are challenging to isolate rather than intrinsically rare (Page 23, Line 650).

Reviewer #3 (Remarks to the Author):

The Authors have addressed all comments and concerns raised by the Reviewer. The explanation regarding the sensitivity of Fisher's combined test to extreme p-values and expansion of Supplementary Table 1 to include key differential expression metrics have adequately resolved the remaining concern regarding analysis. Overall, the study provides important insights into the role of lymphatic endothelial venules in facilitating tumor immune evasive mechanisms, and presenting ALOX12 as a key mediator. The findings add mechanistic depth to our understanding of lymphatic–immune interactions in cancer.

Response: We thank the reviewer for the positive evaluation and for acknowledging that the concerns have been adequately addressed.

Intravital microscopy conducted by different laboratories demonstrated that high endothelial venules (HEVs) in metastasis-containing lymph nodes facilitate the dissemination of tumor cells into the bloodstream. In addition, other investigators found that lymph node (LN) metastatic cells have a higher capacity to escape immunosurveillance due to chronic IFN signaling, resulting in enhanced distant metastases.

The authors confirm these observations in mouse models of breast cancer LN metastases and provide mechanistic data that HEVs enable the immunosuppressive features of tumor cells. They express the enzyme Alox 12 that produces the metabolite 12-HETE, which, upon uptake by tumor cells, binds to ADAR1 and "immunogenically silences" tumor cells through increased adenosine-to-isosine editing.

These data are very interesting, providing mechanistic insights that support the concept that tumor cells traveling through LNs are less immunogenic and have a higher capability to seed as distant metastases.

The authors utilized the elegant photoconvertible Dendra system to distinguish between tumor cells disseminating via lymph nodes (LNs) and other routes to seed in lung metastases, confirming that LN-derived tumor cells have a higher seeding capability in the lung. Although they propose that these are tumor cells that have disseminated via HEVs, they cannot exclude that these tumor cells disseminated via other blood vessels in the LN, or lymphatics. Can the authors clarify whether they performed lymphatic ligation to induce tumor cells to disseminate via the lymphatic blood vessels?

The authors provide scRNA-seq data showing that HEVs in LNs selectively express Alox12, further supporting their hypothesis that HEVs drive the immune recognition of tumor cells. The UMAP predominantly shows other cell types and includes the primary tumor, whereas the endothelial cell population appears very small and underrepresented. It would be more relevant to simply show a UMAP of the different endothelial cell populations in the LNs to increase the resolution of these cells, allowing for the observation of differences between non-HEVs, HEVs, and LECs. How many endothelial cells and HEVs were sequenced?

The authors claim that Alox 12 is specifically expressed in HEVs, but not in other endothelial cells. However, publicly available data sets from human lymph nodes (LNs) reveal that Alox 12 does not appear to be expressed in HEVs (doi: 10.1016/j.immuni.2019.06.027; includes LECs and BECs; doi: 10.1038/s41556-022-00866-3 sc-atlas of non-hematopoietic cells in human LNs). Further analysis of unpublished LN endothelial cells (about 20,000 cells) from our group revealed similar results in murine LNs, detecting Alox 12 in non-HEV blood endothelial cells, but not in HEVs (Chst4+, Fut7+) of LNs.

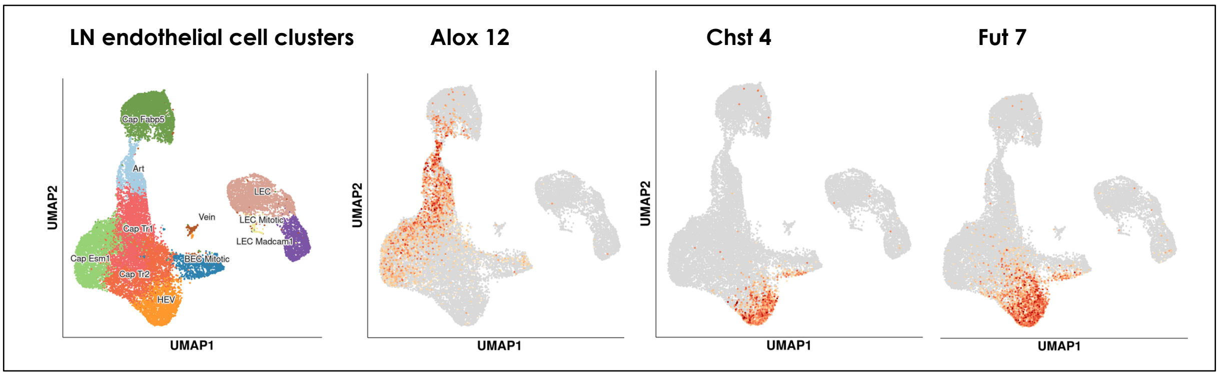

Therefore, it appears that Alox 12 is predominantly expressed in blood endothelial cells in LNs. Could it be that HEVs undergo changes in metastatic LNs that induces Alox 12 expression? Still, the majority of Alox 12 would come from BECs. The Alox 12 protein staining overlaps with HEVs; however, given that platelets express high levels of Alox 12, could this be due to platelet contamination?Given that 12-HETE is secreted, any cell that produces Alox 12 can contribute to the effects the authors observed.
